# First insights into microbial changes within an Inflammatory Bowel Disease Family Cohort study

**DOI:** 10.1080/19490976.2025.2559119

**Published:** 2025-10-05

**Authors:** Philipp Rausch, Ilka Ratjen, Lukas Tittmann, Janna Enderle, Eike Matthias Wacker, Kathrin Jaeger, Malte Christoph Rühlemann, Katrin Franzpötter, Pierre Ellul, Robert Kruse, Jonas Halfvarson, Dirk Roggenbuck, David Ellinghaus, Gunnar Jacobs, Michael Krawczak, Stefan Schreiber, Corinna Bang, Wolfgang Lieb, Andre Franke

**Affiliations:** a Institute of Clinical Molecular Biology, University Hospital Schleswig-Holstein, University of Kiel, Kiel, Germany; b Institute of Epidemiology, University Hospital Schleswig-Holstein, University of Kiel, Kiel, Germany; c Popgen biobank, University Hospital Schleswig-Holstein, University of Kiel, Kiel, Germany; d Department of Internal Medicine II, University Cancer Center Schleswig-Holstein, University Hospital Schleswig-Holstein, Kiel, Germany; e Institute of Human Nutrition and Food Science, University of Kiel, Kiel, Germany; f Division of Gastroenterology, Department of Medicine, Mater Dei Hospital, Msida, Malta; g Department of Gastroenterology, Faculty of Medicine and Health, Örebro University, Örebro, Sweden; h Institute of Biotechnology, Faculty Environment and Natural Sciences, Brandenburg University of Technology Cottbus-Senftenberg, Senftenberg, Germany; i Medipan/GA Generic Assays GmbH, Blankenfelde-Mahlow, Germany; j Institute of Medical Informatics and Statistics, University Hospital Schleswig-Holstein, University of Kiel, Kiel, Germany; k Department of Internal Medicine I, University Hospital Schleswig-Holstein, Kiel, Germany

**Keywords:** Family Cohort, microbiota, 16S, dysbiosis, IBD, inflammatory bowel disease, oralization

## Abstract

The prospective Kiel Inflammatory Bowel Disease (IBD) Family Cohort Study (KINDRED cohort) was initiated in 2013 to systematically and extensively collect data and biosamples from index IBD patients and their relatives, a population at high risk for IBD development. Regular follow-ups were conducted to collect updated health and lifestyle information, to obtain new biosamples, and to capture the incidence of IBD during development. By combining microbial data collected at successive time points with extensive anthropometric, medical, nutritional, and social information, this study aimed to characterize the factors influencing the microbiota in health and disease via detailed ecological analyses. Using a microbial dysbiosis metric based on the German KINDRED cohort, we identified strong and generalizable gradients within and across different external IBD cohorts for validation. These community gradients correspond strongly with IBD pathologies, physiological manifestations of inflammation (*e.g*. Bristol stool score, ASCA IgA, ASCA IgG), and genetic risk for IBD. Anthropometric and medical factors influencing fecal transit time strongly modify bacterial communities. Various *Enterobacteriaceae* (*e.g. Klebsiella sp*.) and opportunistic *Clostridia* pathogens (*e.g. C. XIVa clostridioforme*), characterize in combination with ectopically colonizing oral taxa (*e.g. Veillonella sp*. *Cand. Saccharibacteria sp*. *Fusobacterium nucleatum*) the distinct and chaotic IBD-specific communities. Weak community and physiological changes are further traceable in a small number of individuals, who developed IBD in the study’s runtime. Our findings demonstrate broad-scale ecological patterns which indicate drastic state transitions of communities in IBD patients. These patterns appear to be universal across cohorts and influence physiological signs of inflammation, display increased resilience, but show only limited heritability/intrafamily transmission.

## Introduction

Inflammatory bowel diseases (IBD), with the most common manifestations Crohn’s disease (CD) and ulcerative colitis (UC), are characterized by chronic, relapsing inflammation of the gastrointestinal tract,^1^ arising from a complex interplay of genetic, lifestyle, and environmental factors.^2–4^ The incidence and prevalence of IBD are rising in many parts of the world,^5,6^ and the highest incidences are currently reported in North America and Europe,^5,7^ while projections expect a disease prevalence of 1% in high-income countries by the year 2030.^8^

Familial clustering of IBD is well documented, underscoring the role of both shared genetics and environment in disease susceptibility.^4,9–12^ Genome-wide association studies have identified hundreds of loci associated with CD and UC, implicating genes involved in epithelial barrier function, immune responses, and the gut microbiota.^4,13,14^ First-degree relatives of IBD patients are at substantially elevated risk of developing the disease,^4,11,15^ and familial aggregation is associated with earlier onset and potentially more severe disease.^16,17^ While most prior microbiome studies of IBD have used cross-sectional, case – control designs, prospective family-based studies are uniquely positioned to identify early molecular and microbial changes that may precede disease onset. These community shifts can be associated to environmental and lifestyle factors, diet, hygiene, medication, or even the host’s genetic makeup.^18–20^ However, such studies are rare due to logistical and resource constraints.^10^

Thus, in 2013, the Inflammatory Bowel Disease Family Cohort (KINDRED cohort), the so-called KINDRED cohort, was initiated in Kiel (Germany) to systematically collect longitudinal clinical, genetic, lifestyle, and microbiome data from IBD patients and their relatives. The primary objectives of the KINDRED cohort are: (i) to investigate the influence of genetic-, environmental- and lifestyle factors on physiology and microbial communities; (ii) to characterize the long-term clinical course of IBD patients; (iii) to identify individuals who develop IBD during follow-up; and (iv) to characterize lifestyle factors and biomarkers that potentially predispose individuals to IBD onset. Further details of the cohort design, recruitment, and data collection are provided in the Materials and Methods section as well as in the Supplemental Methods.

In this study we investigate (i) clinical characteristics and fecal microbiome characteristics of the KINDRED cohort with respect to IBD pathology. Furthermore, (ii) we explore the influence and association of environmental-, lifestyle-, and genetic factors on the microbial communities and (iii) investigate a small subgroup of individuals who developed IBD in the observation period.

## Results

### Current status of the KINDRED cohort

As of April 2021, the Kiel KINDRED cohort included 1497 IBD patients and 1813 initially non-affected family members, belonging to a total of 1372 families. The data of all study participants whose baseline questionnaires were double entered and quality-checked were extracted from the database in a data freeze in March 2021, comprising 2393 individuals (1321 IBD patients and 1072 non-affected relatives). The discrepancy in the total number of study participants is due to the time lag between study inclusion and the return of biomaterial and questionnaire data (including quality check). The baseline characteristics of the healthy participants, stratified by age, and of the IBD patients, stratified by disease type (CD-Crohn’s disease; UC-ulcerative colitis; uIBD-unclassified inflammatory bowel disease), are summarized in Table 1 and Tables S1-S4, respectively (see also Supplemental Methods). Sampling time points were separated by an average of 2.652 ± 0.374 years between BL and F1, and 1.563 ± 0.326 years between F1 and F2.

**Table 1.** Main characteristics of the Kiel IBD Family Cohort (KINDRED). Values are mean ± standard deviation or absolute counts. Abbreviations: CD, Crohn’s disease; IBD, inflammatory bowel disease; UC, Ulcerative colitis, uIBD, unclassified inflammatory bowel disease.

	Baseline				Follow-up 1				Follow-up 2			
Characteristics	Control	CD	UC	uIBD	Control	CD	UC	uIBD	Control	CD	UC	uIBD
# Participants	791	551	438	32	362	174	105	7	295	146	92	6
Sex												
Male	316	175	166	13	140	57	38	1	103	52	34	0
Female	432	375	272	19	185	117	67	6	152	94	56	6
Age at baseline assessment, years	44.14 ± 19.44	43.99 ± 15.58	46.42 ± 14.62	41.63 ± 15.11	45.60 ± 19.03	43.95 ± 14.63	48.64 ± 14.37	49.71 ± 19.72	47.56 ± 18.06	47.03 ± 14.23	48.65 ± 14.82	55 ± 15.18
Age groups												
Children (7–11 years)	46	6	0	0	28	1	0	0	8	1	0	0
Adolescents (12–17 years)	47	24	8	2	33	11	0	0	11	3	0	0
Adults (≥18 years)	698	521	430	30	333	162	105	7	286	142	92	6
BMI (kg/m^2^) average	24.98 ± 5.33	24.05 ± 4.74	24.60 ± 4.65	24.92 ± 4.88	25.18 ± 4.98	24.51 ± 5.28	24.65 ± 4.27	26.86 ± 3.57	26.15 ± 12.33	24.45 ± 4.71	24.99 ± 4.09	24.00 ± 5.30
BMI Children (7–11 yrs)	16.50 ± 2.47	14.78 ± 1.83	–	–	17.97 ± 2.68	14.18	–	–	16.89 ± 0.24	16.66	–	–
BMI Adolescents (12–17 yrs)	20.18 ± 3.18	18.81 ± 2.54	17.11 ± 1.72	22.65 ± 3.13	20.66 ± 2.40	18.85 ± 2.64	–	–	22.37 ± 4.62	17.23 ± 1.15	–	–
BMI Adults (≥18 years)	25.81 ± 4.92	24.36 ± 4.60	24.74 ± 4.58	25.07 ± 4.98	25.70 ± 4.81	24.96 ± 5.15	24.65 ± 4.27	26.86 ± 3.57	26.36 ± 12.48	24.66 ± 4.61	24.99 ± 4.09	24.00 ± 5.30
Smoking Status												
never smoked	392	260	200	18	181	83	50	4	147	71	46	4
smoked less than 3 months	65	29	32		26	9	8	–	15	5	4	0
smoked in the past	201	185	181	12	84	62	39	2	70	55	30	1
smoking now	87	76	25	1	30	19	6	1	20	14	8	0
Age at IBD diagnosis (yrs)	–	24.42 ± 12.22	29.94 ± 13.66	23.1 ± 18.68	–	22.51 ± 10.51	26.74 ± 10.14	71	–	23.63 ± 11.17	28.06 ± 13.77	33.33 ± 33.72
Disease location for CD (multiple sites possible)												
not known	–	11	–	1	–	1	1	2	1	3	–	1
middle GI tract (proximal)	–	4	–	–	–	4	–	–	–	1	–	–
middle GI tract (distal)	–	9	1	–	–	6	–	–	–	2	–	–
upper GI tract	–	15	–	–	1	11	–	–	1	10	–	–
ileum	–	44	–	–	–	23	–	–	2	12	–	–
terminal ileum	2	145	1	2	–	67	1	–	8	51	1	1
ileum and colon	1	86	2	1	–	49	–	1	2	44	–	–
colon	2	75	16	6	–	44	6	3	2	32	–	3
perianal		30	1	1	–	15	–	–	1	8	–	–
Disease location for UC (multiple sites possible)												
not known	–	–	6	1	–	1	1	6	2	–	2	1
proctitis	–	–	43	–	1	2	12	–	3	–	16	–
left colon	1	–	83	1	–	1	33	–	5	–	23	–
pancolitis	2	–	128	3	–	3	47	–	2	1	33	–
Medication												
Yes	479	536	417	30	205	167	99	6	163	130	80	6
No	312	15	21	2	157	7	6	1	132	16	12	0
antidiarrhetics	0	57	41	2	0	29	11	0	5	34	7	0
antibiotics	133	142	99	10	67	41	27	2	28	39	18	1
antiinflammatory	7	470	364	27	2	155	84	6	14	103	59	5
Type of IBD medication (last 12 months)												
Immunosuppressives	3	96	78	4	1	47	16	1	5	32	17	1
Glucocorticoids	1	39	29	0	0	29	15	3	4	33	12	0
5ASA	3	30	109	6	0	43	50	4	7	37	46	5
Mesalazin	0	117	112	11	0	49	57	4		-	–	–
Sulfasalazin	0	24	16	0	0	12	4	0		-	–	–
AZA	0	99	42	5	0	40	13	1		-	–	–
Biologicals/‘small molecules’	1	77	45	1	0	54	16	0	4	46	12	0
IBD surgery												
Yes	2	364	78	4	0	116	83	1	0	98	19	0
No	789	187	360	28	362	58	22	6	295	48	73	6
Number of future onsets (IBD next time point)	4	–	–	–	1	–	–	–	2	–	–	–

**Figure 1.**
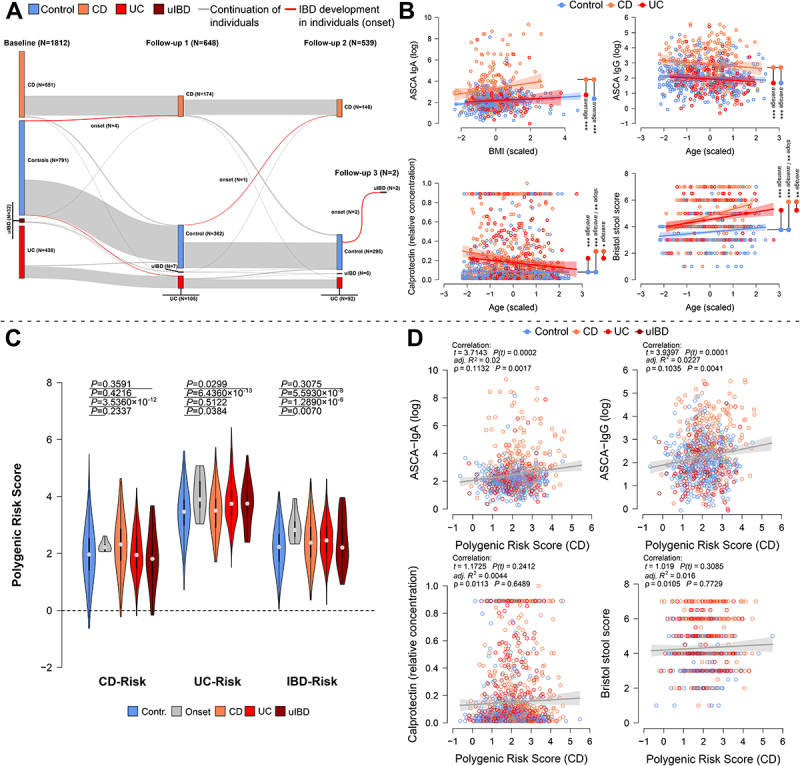
(A) the flow diagram displays the change of the cohort size and its subsets between time points. The highlighted transitions are onset cases detected during the study’s runtime (N_BL→F1_=4 (CD:2, UC:2), N_F1→F2_=1 (CD:1), N_F2→F3_=2 (uIBD:2)). (B) Analyses of selected physiological inflammation markers (ASCA IgA, ASCA IgG, calprotectin, Bristol stool score) with respect to IBD condition and relevant covariates via linear models at baseline (see Table 1, Table S5 for additional time points). Optimal model results (min. AIC) are shown and average pairwise differences with respect disease groups and/or differences in group slopes/direction of association are highlighted in the individual plots (# *p*≤0.1000, * *p*≤0.0500, ** *p*≤0.0100, *** *p*≤0.0010). (C) The violin plot displays the average differences of genetic predisposition to CD, UC, and IBD in general, as based on *LDpred2* derived polygenic risk scores (PRS). The average differences in PRS were tested via Wilcoxon rank tests contrasting healthy individuals (*N*=785), with healthy future onset cases (grey; N_BL_=4, N_F1_=1, N_F2_=2), CD patients (orange; *N*=551), UC patients (red; *N*=438), and patients with unclassified IBD (uIBD; darkred; *N*=32). In particular, compared with healthy controls, the average risk of IBD is significantly and consistently higher in patients with CD and UC, as well as in future onset cases. (D) Scatterplots show the significant relationships between CD-PRS and selected physiological inflammation markers at the baseline time point, considering anthropometric covariates (linear model on residuals) and general correlation without additional covariates (Spearman correlation). Additional analyses of IBD-PRS and UC-PRS with physiological markers can be found in Figure S1 and Table S6.

Available EDTA blood samples from the baseline collection (BL) were used to generate DNA for genome-wide genotyping (Global Screening Array (GSA), version 2.0 (Illumina), N = 2625). Baseline data were complemented by fecal 16S rRNA-based microbiome profiles (N = 1812), blood biomarker profiles (C-reactive protein, N = 316; hemoglobin, N = 434; mainly IBD patients), serum antibodies (ASCA IgA, N = 781; ASCA IgG, N = 780; GP2 IgA, N = 781; GP2 IgG, N = 781), and fecal indicators of inflammation (calprotectin, N = 1763; occult hemoglobin/haptoglobin status, N = 1760; Bristol stool score, N = 882). For a subset of the first and second follow-ups (N_F1_ = 648, N_F2_ = 539) 16S rRNA sequencing data, fecal calprotectin measurements (N_F1_ = 519, N_F2_ = 147), hemoglobin/haptoglobin measurements (N_F1_ = 516, N_F2_ = 144), Bristol stool score (N_F1_ = 316, N_F2_ = 255) and blood biomarkers of inflammation (Hb: N_F1_ = 203, N_F2_ = 162; CRP: N_F1_ = 179, N_F2_ = 127) were also available. In addition, our study set included 7 cases of IBD onset with data available before IBD diagnosis (N_BL→F1_ = 4, N_F1→F2_ = 1, N_F2→F3_ = 2; see Figure 1(A)) and four onset cases with data before and after onset (N_BL→F1_ = 3, N_F1→F2_ = 1). Follow-up 3 (F3) consisted here of only two IBD onset cases, which were diagnosed with IBD after the second follow-up to increase analytic power. See Materials and Methods section and Supplemental Methods section for additional details on sampling and data generation. Unless otherwise stated, analyses were performed on the baseline set of samples (N = 1812).

### Distribution of physiological biomarkers of inflammation in the KINDRED cohort

In baseline samples, the concentrations of the IBD biomarker and antifungal antibody anti-*Saccharomyces cerevisiae* antibody (ASCA) IgG increased with BMI, while ASCA IgA decreased with age (Table 2). ASCA IgA and IgG antibody levels were also significantly elevated in CD patients compared to healthy controls and UC patients (Figure 1(B)). The concentration of *anti-glycoprotein 2* IgA and IgG (GP2), biomarkers for PSC and CD,^21–23^ significantly increased in IBD patients, and were associated with patient age and sex. The concentration of GP2 Ig’s decreased in females and increased in males with age. Levels of GP2 Ig’s were highest in CD patients compared to UC patients and control subjects (Table 2, Figure S1). The fecal calprotectin concentration, a general measure gut inflammation, was highest in CD patients compared to followed by UC patients and lowest in healthy controls. Fecal calprotectin levels also decreased with subject age and declined more rapidly in CD patients compared to control subjects (Figure 1(B)). The concentration of C-reactive protein (CRP), measured only in IBD patients, increased with BMI and aging UC patients (Figure S1). Hemoglobin (Hb) levels, also measured only in IBD patients, increased with BMI with a stronger increase in males compared to females (Figure S1). Stool consistency strongly and significantly decreased in older subjects, as expressed by an increase of the self-reported Bristol stool score (BSS). This indicates a faster transit time/softer stool in older individuals, particularly aging CD patients (Figure 1(B); Table 2). The associations were mostly stable between the analyses of baseline samples and the follow-up time points for shared variables (Table S5).

**Table 2.** Analysis of major physiological characteristics at the baseline time point using linear models. Results depicted are models after variable selection minimizing AIC.

							Mean		Slope	
Trait	Model Factors	*DF*	*F*	*P*	*adj R2*	Comparison	*P*	*P(FDR)*	*P*	*P(FDR)*
ASCA IgA	BMI#	1,735	3.74197	0.05345	0.18346	CD – Contr.	1.731 × 10^−32^	5.192 × 10^−32^	–	–
(log)	IBD	2,735	82.53814	4.602 × 10^−33^		CD – UC	2.387 × 10^−18^	3.581 × 10^−18^	–	–
						Contr. - UC	0.35078	0.35078	–	–
ASCA IgG	Age#	1,760	5.08400	0.02443	0.16775	CD – Contr.	4.002 × 10^−28^	1.201 × 10^−27^	–	–
(log)	IBD	2,760	75.85850	9.170 × 10^−31^		CD – UC	5.558 × 10^−21^	8.337 × 10^−21^	–	–
						Contr. - UC	0.41353	0.41353	–	–
GP2 IgA	Sex	1.733	0.12325	0.72564	0.08787	CD – Contr.	4.036 × 10^−14^	1.211 × 10^−13^	–	–
(log)	Age	1.733	5.29017	0.02173		CD – UC	6.557 × 10^−7^	9.835 × 10^−7^	–	–
	IBD	2.733	31.07387	1.113 × 10^−13^		Contr. - UC	0.24487	0.24487	–	–
	Sex:Age	1.733	8.530278	0.00360		M-F	0.29527	0.29527	0.00360	0.00360
GP2 IgG	Sex	1.755	1.34058	0.24730	0.05018	CD – Contr.	2.976 × 10^−8^	8.927 × 10^−8^	–	–
(log)	Age	1.755	0.33994	0.56004		CD – UC	8.903 × 10^−5^	1.335 × 10^−4^	–	–
	IBD	2.755	16.92608	6.444 × 10^−8^		Contr. - UC	0.61424	0.61424	–	–
	Sex:Age	1.755	9.61766	0.00200		M-F	0.08763	0.08763	0.00200	0.00200
CRP (X^1/4^)	Age#	1,295	0.21140	0.64601	0.03229	CD – UC	0.96866	–	0.04518	–
(only CD & UC)	BMI#	1,295	9.71435	0.00201			-	–	–	–
	IBD	1,295	0.00497	0.94385			-	–	–	–
	Age#:IBD	1,295	4.04635	0.04517			-	–	–	–
HB (X^2^)	Sex	1,414	123.8936	2.363 × 10^−25^	0.2408		-	–	–	–
(CD & UC)	BMI#	1,414	10.0727	0.0016			-	–	–	–
Calprotectin (rel.)	Age#	1.1725	6.10170	0.01360	0.05915	CD – Contr.	7.039 × 10^−21^	2.112 × 10^−20^	0.00898	0.02693
	IBD	2.1725	50.10117	7.077 × 10^−22^		CD – UC	2.382 × 10^−2^	2.382 × 10^−2^	0.47861	0.47861
	Age#:IBD	2.1725	3.73413	0.02409		Contr. - UC	2.716 × 10^−10^	4.074 × 10^−10^	0.13797	0.20695
Bristol stool score	Age#	1.861	32.69355	1.488 × 10^−8^	0.18469	CD – Contr.	2.596 × 10^−31^	7.789 × 10^−31^	0.00133	0.00399
	IBD	2.861	79.04321	3.048 × 10^−32^		CD – UC	0.00321	0.00321	0.07856	0.11784
	Age#:IBD	2.861	5.19376	0.00573		Contr. - UC	2.688 × 10^−18^	4.032 × 10^−18^	0.23382	0.23382

#centered and scaled.

### Genetic predisposition to IBD (polygenic risk scores) is associated to physiological signs of inflammation in the KINDRED cohort

Due to the increasing number of variants associated to IBD, it has become possible to quantify an individual’s genetic risk or genetic predisposition to CD, UC, or IBD using polygenic risk scores (PRS).^24^ Based on the available genotyping data, we derived CD-PRS, UC-PRS, and a combined score for both pathologies IBD-PRS.^24^ All three PRS variants were elevated in IBD patients, compared to healthy individuals. CD- and UC-PRS were disease-specific as they only showed significantly elevated risk in the corresponding disease subset, while the general IBD-PRS was increased in CD and UC patients alike (Figure 1(C)). Interestingly, the average UC-PRS and general IBD-PRS were significantly elevated among future onset cases compared to the remaining healthy controls, while CD-PRS showed no significant increase. Healthy first-degree relatives of diseased individuals (FDRs) showed no significantly elevated PRS compared to more distant healthy relatives ( >1st degree relatives), or unrelated controls (Figure S3). These findings suggest a greater accumulation of risk variants in current and prospective patients, even in comparison to closely related family members of IBD patients.

We also detected significant positive relationships between CD- and IBD-PRS and of ASCA IgA and IgG levels at baseline. Antibody levels against GP2 also significantly correlated with higher genetic risk for IBD, linking genetic risk for IBD and immune responses to potential environmental antigens (IBD-PRS vs. GP2 IgA; Figure 1(D), Figure S4, Table S6). Other inflammatory markers, such as calprotectin, BSS, CRP, and Hb, showed only weak associations with the polygenic risk scores (Figure 1(D), Figure S4, Table S6). However, after correction for disease state, only ASCA IgG levels remained significantly associated to CD-PRS (*p* = 0.03555).

### Differential abundance of microbial taxa across IBD pathologies and time

**Figure 2.**
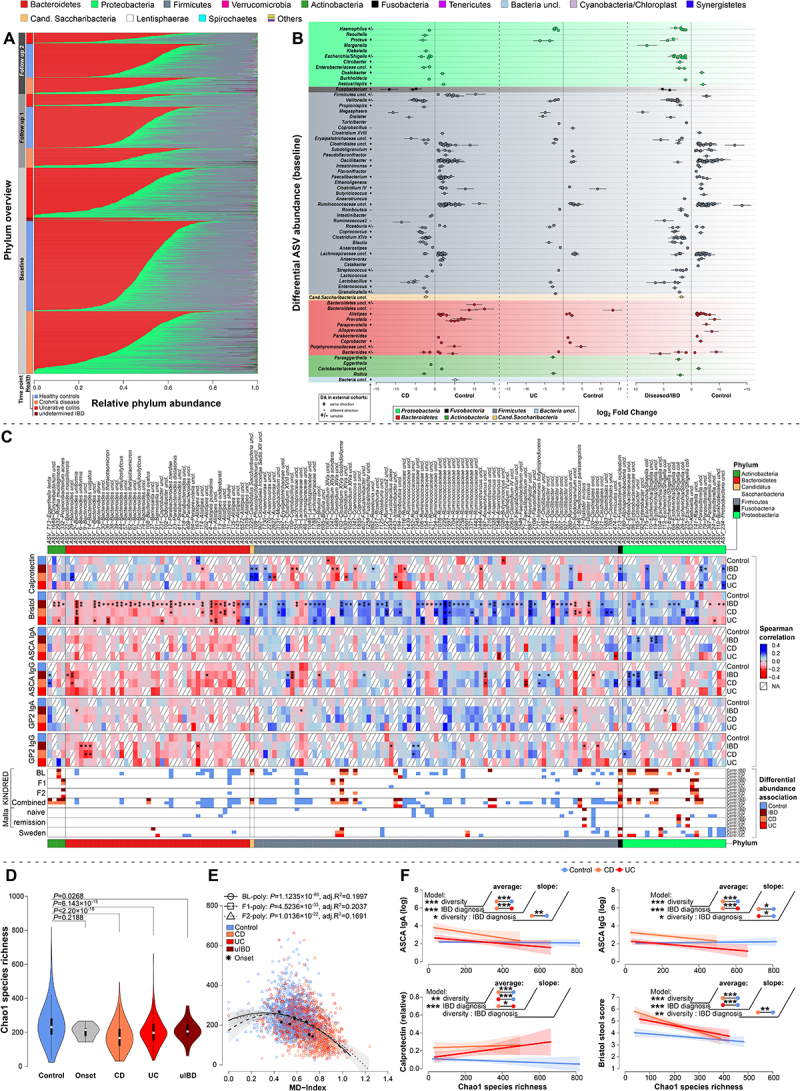
Phylum and ASV level abundances with respect to IBD status, association to clinical measures of inflammation and analyses of alpha diversity. (A) Overview of individual phylum abundances across time points and health conditions in the KINDRED cohort. (B) Differential abundance analyses of ASVs based on the baseline time point (BL). Displayed are the log Fold changes for each significantly different ASV clustered by genus classification, including standard errors. Color coding indicates the phylum membership of the ASV. The plot only displays taxa with significant differential abundance in the respective comparisons. (C) Partial correlation of CLR transformed taxon abundances with core physiological measures via *ppcor*,^25^ combining the *p* values of Spearman-, Kendall-, and Pearson correlations via Brown’s method and corrected via FDR.^26^ Correlations were adjusted for age, gender, and BMI and Spearman *ρ* is used to visualize correlation strength between taxa and clinical measures. Overlapping patterns of differential abundance for the respective taxa in the KINDRED cohort, Maltese-, and Swedish cohort is indicated in the bottom color bars. (D) Differences in Chao1 species richness between healthy individuals, future onset cases, CD, UC, and uIBD cases at baseline with additional onset cases (N_onset_ = 7, N_CD_ = 551, N_UC_ = 438, N_uIBD_ = 32, N_contr._ = 785; Wilcoxon test; see Materials and Methods). (E) Correlation of species richness with the microbial dysbiosis index (MD-index), which results in a negative, but non-linear relationship (optimal AIC based fit, Table S8) between diversity and dysbiosis (BL (polynomial): *F*_2,1809_=226.97, *p*<2.2×10^−16^, *adj.R*^*2*^ = 0.1997, *N* = 1812; F1 (polynomial): *F*_2,645_ = 83.778, *p*<2.2×10^−16^, *adj.R*^*2*^ = 0.2037, *N* = 648; F2 (polynomial): *F*_2,536_ = 55.745, *p*<2.2×10^−16^, *adj.R*^*2*^ = 0.1691, *N* = 539; linear model). (F) Relationship between Chao1 species richness measured at baseline and several IBD relevant clinical measures, which shows significant relationships between alpha diversity and host physiology, in a disease-specific manner (see Table S9, excluding uIBD). Average pairwise differences between disease groups, or differences in slope/direction of association are highlighted in the plots (# *p*≤0.1000, * *p* ≤ 0.0500, ** *p* ≤ 0.0100, *** *p* ≤ 0.0010).

Across all three sampling points, fecal communities were primarily dominated by Bacteroidetes, followed by Firmicutes, Proteobacteria, and minor groups such as Fusobacteria, Tenericutes, and Candidatus Saccharibacteria (Figure 2(A)).

At the ASV level, over 750 taxa showed significant differential abundance among major IBD pathologies (BL: 294, F1: 150, F2: 193, combined: 536; Figure 2(B)). At baseline, ASVs from Enterobacteriaceae (e.g. *Haemophilus, Escherichia/Shigella, Raoultella, Klebsiella*) were highly abundant in IBD patients, especially in CD patients, and persisted over time (Figures 2(B), S5, S6). Firmicutes such as *Veillonella*, *Enterococcus*, and *Granulicatella* were more abundant in both CD and UC patients, while *Ruminococcaceae* (e.g., *Clostridium IV*, *Oscillibacter*, *Faecalibacterium*) were enriched in healthy individuals. Most *Bacteroidetes* (*Alistipes, Prevotella, Bacteroides*) were also more abundant in healthy individuals and showed similar patterns in external cohorts (Figures 2(B), S5, S6; Tables S10–S13).

Interestingly, several IBD-associated taxa, like *Veillonella*, *Dialister*, *Granulicatella* (Firmicutes), *Haemophilus*, *Klebsiella* (Proteobacteria), and *Rothia* (Actinobacteria), are indicators of oralization in the gut microbiome. Similarly, the rarely described, disease-associated oral taxon *Cand. Saccharibacteria*^27^ was more abundant in the fecal communities of CD patients. *Fusobacteria* (e.g. *F. nucleatum*) linked to colorectal cancer and inflammation,^28^ were also more abundant in CD patients and were replicated in the external validation cohorts (Maltese treatment naive and remission patients, Swedish treatment naive patients). Most IBD-associated taxa, including *Veillonella*, *Enterobacteriaceae*, or *Clostridium XIVa clostridioforme*, were confirmed in the external cohorts (Figure 2(B), S5, S6, Table S10-S13).

Various differentially abundant taxa correlated with clinical indicators of inflammation and genetic risk scores for IBD. Indicator bacteria of oralization correlated with physiological signs of inflammation (e.g. *R. mucilaginosa-* BSS ↓, *Cand. Saccharibacter*- calprotectin ↑, *Klebsiella uncl.-* BSS ↑, *F. nucleatum*- calprotectin ↑, *Dialister invisus-* BSS↓ & GP2 IgA/IgG ↓, *Veillonella uncl.-* IBD-PRS ↑; Figure 2(C), Figure S7). Most Firmicutes correlated with softer stool (BSS) in IBD patients (*e.g. Clostridium XlVa clostridioforme Ruminococcaceae uncl*.), while Bacteroidetes correlated with harder stool (low BSS; see Figure 2(C); Table S14). IBD-associated *Enterobacteriaceae* correlated positively with ASCA IgG/IgA, calprotectin levels, as well as softer stools (higher BSS), only in IBD/CD patients but not in healthy individuals (Figure 2(C); *e.g*. ASV-99 *Escherichia/Shigella coli*).

Certain taxa were also associated to the polygenic risk scores for IBD (PRS). Bacteroidetes (e.g. *Alistipes*) were negatively correlated with genetic risk for CD, UC, and IBD, except for *Bacteroides fragillis*, which showed positive associations (Figure S7, Table S14). *Enterobacteriaceae (e.g*. ASV-42 *Enterobacteriaceae uncl.*, ASV-165 *Citrobacter uncl.)*, as well as members of the *Clostridium XlVa* group correlated with higher genetic IBD risk (IBD-PRS, CD-PRS), particularly in IBD patients and not in healthy individuals (Figure 2(C) & Figure S7; Table S14). Several SCFA producers in the Firmicutes correlated with lower levels of ASCA IgG/IgA and decreasing genetic risk for IBD risk (*e.g*. ASV-71 *Faecalibacterium uncl*.), while others (*e.g. Blautia, Ruminococcacea uncl*.) correlated with increasing genetic risk, particularly in diseased individuals. Overall, correlations between bacteria, clinical parameters, and genetic risk were generally disease-specific and rarely significant in UC patients or healthy controls.

### Alpha diversity patterns associated with IBD pathology, clinical characteristics, and microbial dysbiosis

A critical characteristic of ecosystems is their complexity, which can be informative for assessing the community state, productivity, or stability and can be based on, *e.g.*, the number of community members or their distribution. We identified an extensive loss of species diversity in CD and UC patients with respect to species richness and abundance distribution at baseline (Figure 2(D)) and the follow-up time points (Figure S8, Table 3). Individuals suffering from CD showed a significantly larger decrease of community richness and complexity than UC patients as compared to healthy controls, while future onset cases showed a slight, yet insignificant reduction of community diversity compared to healthy individuals (Figure 2(D), Figure S8; baseline samples including all available onset cases: N_onset_ = 7, N_CD_ = 551, N_UC_ = 438, N_uIBD_ = 32, N_contr._ = 785).

**Table 3.** Analysis of alpha diversity patterns across time points, correcting for age, sex, and BMI.

							Pairwise comparison	
TP	Diversity	Model	*DF*	*F*-value	*P*-value	*adj. R*^*2*^	Contrast	*P* (adjusted)
BL	Shannon	Age#	1,1696	4.1850	0.0409	0.12331	CD-Contr.	2.8584 × 10^−12^
	(effective)	BMI#	1,1696	0.0055	0.9406		CD-UC	2.7729 × 10^−6^
		Sex	1,1696	2.0336	0.1540		Contr.-UC	2.8836 × 10^−12^
		**IBD diagnosis**	2,1696	**119.0098**	**4.3082 × 10**^**−49**^			
	Chao	Age#	1,1696	4.4142	0.0358	0.1204	CD-Contr.	2.8584 × 10^−12^
		BMI#	1,1696	0.0289	0.8649		CD-UC	2.5050 × 10^−7^
		Sex	1,1696	1.6340	0.2013		Contr.-UC	2.8896 × 10^−12^
		**IBD diagnosis**	2,1696	**115.8919**	**6.6629 × 10**^**−48**^			
F1	Shannon	Age#	1,593	3.2560	0.0717	0.1398	CD-Contr.	4.8285 × 10^−10^
	(effective)	BMI#	1,593	0.2953	0.5870		CD-UC	0.0013
		Sex	1,599	3.8538	0.0501		Contr.-UC	0.0001
		**IBD diagnosis**	2,593	**47.3911**	**8.0637 × 10**^**−20**^			
	Chao	Age#	1,593	3.4752	0.0628	0.1111	CD-Contr.	4.8285 × 10^−10^
		BMI#	1,593	0.9530	0.3294		CD-UC	0.0045
		Sex	1,599	0.2596	0.6106		Contr.-UC	0.0007
		**IBD diagnosis**	2,593	**37.5122**	**4.5685 × 10**^**−16**^			
F2	Shannon	Age#	1,478	1.7441	0.1873	0.1399	CD-Contr.	4.5103 × 10^−11^
	(effective)	BMI#	1,478	0.0472	0.8281		CD-UC	0.0041
		Sex	1,478	0.0123	0.9116		Contr.-UC	0.0001
		**IBD diagnosis**	2,478	**40.8792**	**4.0844 × 10**^**−17**^			
	Chao	Age (scaled)	1,478	5.0238	0.0255	0.1294	CD-Contr.	4.5112 × 10^−11^
		BMI (scaled)	1,478	2.6772	0.1025		CD-UC	0.0005
		Sex	1,478	0.3441	0.5577		Contr.-UC	0.0108
		**IBD diagnosis**	2,478	**34.3795**	**1.1221 × 10**^**−14**^			

#scaled and centered.

Based on the differential abundance patterns we derived an index of community condition and level of dysbiosis, the Microbial Dysbiosis index (MD-index) as introduced by Gevers et al.^29^ (see Materials and Methods). The MD-index showed an overall negative, yet not linear correlation with alpha diversity, implying an accelerated loss of diversity with increasing dysbiosis (breakpoint at MD = 0.615, Figure 2(E) & Figure S8, Table S8). Across all time points we identified a trend of strongly decreasing alpha diversity in UC and CD patients with increasing dysbiosis, while healthy controls showed a far slower decline of diversity under increasing dysbiosis (Table S15). In the baseline dataset we observed strong negative correlations with physiological indicators of inflammation. Alpha diversity metrics most strongly decreased with increasing levels of ASCA IgA, ASCA IgG, calprotectin, and BSS, particularly in IBD patients. These patterns implied more severe signs of inflammation in individuals with lower community diversity or vice versa, which is particularly pronounced in CD patients (Figure 2(F), Table S9).

### Microbial community differences between IBD pathologies are associated with physiological and anthropometric characteristics, patterns of dysbiosis, and genetic risk for IBD

**Figure 3.**
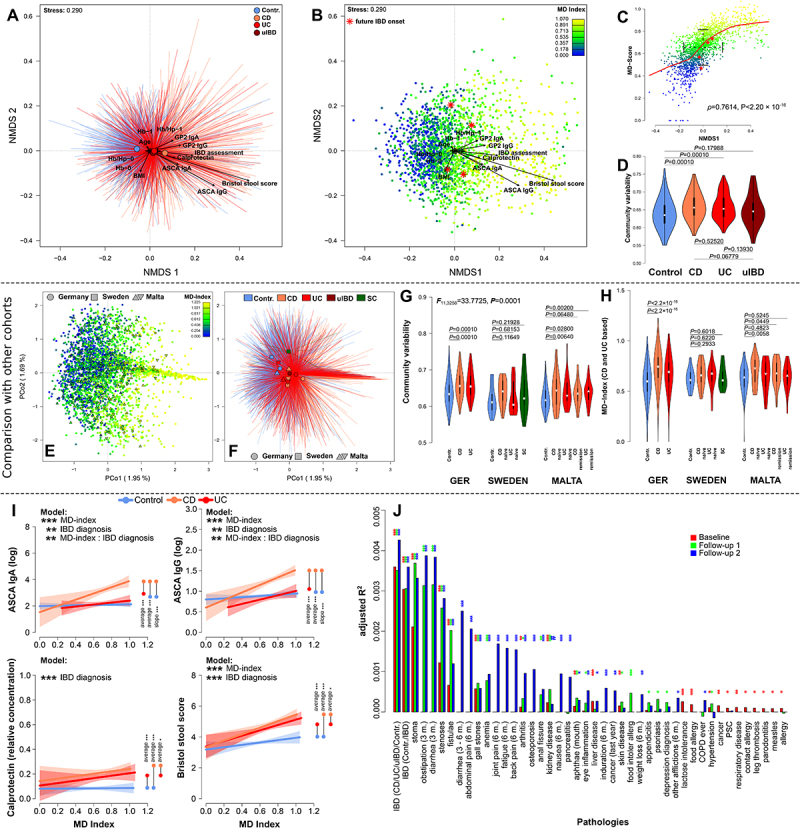
(A) Non-metric multidimensional scaling (NMDS) of Bray-Curtis distances among baseline samples, displaying the significant clustering by health conditions and significant correlations of clinical inflammation measures with community distance (BL, see Table S16 &amp; Table S17). (B) NMDS displaying the gradient of community dysbiosis as expressed by MD-index,^29^ in parallel with clinical measures of inflammation and healthy onset cases highlighted in red (*, develop IBD in F1). (C) Correlation of MD-index and the first NMDS axis showing a clear gradient of dysbiosis in the community. Onset cases are distributed within the range of standard deviation around the mean of the community distribution (NMDS1) and the MD-index. (D) Community variability between health conditions as measured by the distance to the group centroid, is overall significantly different between health conditions (*F*_3,1808_ = 46.0315, *p* = 0.00001, PERMANOVA), and significantly increased in CD and UC patients as compared to healthy controls (Figure S11; Table S18). (E) Principle coordinate analysis of German-, Swedish-, and Maltese samples, highlighting the transferability of the dysbiosis gradient across cohorts (MD-index derived from German samples), (F) as well as a common disease wise clustering of communities irrespective of sample origin (Table S20). (G) Community variability between health/IBD conditions within and between the German-, Swedish-, and Maltese cohorts showing an increased variability in IBD cases. (H) Mean differences of dysbiosis (MD-index derived from German samples) within and across cohorts, with the strongest differences between healthy and CD individuals.(I) Line plots visualize the correlation of selected physiological inflammation markers with the microbial dysbiosis index (MD-index),^29^ and show disease-specific differences as compared to healthy control individuals. Significant pairwise comparisons with respect to average differences between pathologies, or differences in slope are highlighted in the plots (see Figure S12, Table S20). (J) Visualization of the explained variation of pathologies significantly associated to community composition based on serial PERMANOVA of Bray-Curtis distances in all three time points (Table S21). Variables are displayed if they show significant clustering in at least one time point (# *P*_*FDR*_≤0.1000, * *P*_*FDR*_ ≤ 0.0500, ** *P*_*FDR*_≤0.0100, *** *P*_*FDR*_ ≤ 0.0010). for beta diversity analyses including physiological variables, pharmaceutical treatments, and nutrient intake see Table S16, Table S21–23 and Figure S9 & S10.

By investigating community dissimilarities, we revealed various patterns of health-associated, anthropometric, and lifestyle-related community differences. In particular, IBD pathology showed a strong association with community composition, with the strongest differences between microbial communities originating from healthy individuals and those originating from CD patients across time points, followed by differences between healthy controls and UC patients (Figure 3(A,J), Figure S9; Table S16 &amp; Table S21 including F1 and F2). However, slight community differences between UC- and CD patients persisted.

We also detected significant correlations of community composition and physiological indicators of inflammation. Increasing levels of ASCA (IgA/IgG) and GP2 (IgA/IgG) were correlated with IBD communities similar to increasing levels of fecal calprotectin or the presence of occult blood in the respective stool samples (BL; see Figure 3(A,B), Figure S9, S10A; Table S16 &amp; Table S17 including F1 and F2). However, the most influential physiological parameter for the community composition was decreasing gut transit time as expressed by increasing BSS particularly in IBD patients. Interestingly the concentrations of serum CRP- and Hb, measured only in IBD patients, showed no significant correlation with community composition.

Community composition was strongly associated with the MD-index (Table S16 incl. F1, F2), reflecting a dysbiosis gradient that paralleled differentiation between healthy and diseased individuals and clinical inflammation markers. The MD-index distinguished IBD from non-IBD individuals and matched disease-specific clustering across cohorts (KINDRED BL, Sweden, Malta; Figure 3(E–H)). These “general” disease patterns were also reflected in the sample clustering by IBD condition, irrespective of cohort origin (dbRDA (conditioned for cohort): *F*_4,3308_ = 4.0406, *p* < 0.0001, *adj. R*^*2*^ = 0.0037) and were supported by significantly greater community variability in IBD patients compared to healthy controls (Figure 3(D,G), Figure S11; Table S18). Interestingly, future onset patients, healthy individuals which will develop IBD within the studies’ runtime were distributed around the center of the dysbiosis gradient (BL, Figure 3(C), Figure S9 including F1 and F2).

The MD-index correlated directly with several physiological signs of inflammation. We detected a positive correlation of the MD-index with levels of ASCA IgG or IgA and GP2 IgG and IgA, particularly in CD patients (Figure 3(I,J), Figure S12; Table S20). Increased BSS (softer stool) correlated with dysbiosis in IBD patients (Figure 3(I)). Levels of calprotectin and BMI were on average higher in CD and UC patients than in healthy controls, but not linked to the MD-index (Figures 3(I) and 1(B); Table S20), nor were serum CRP- and Hb (Figure S12; Table S20). The IBD severity patients (1-no/4-severe inflammation), positively correlated with MD-index, particularly in CD patients (Figure S12; Table S20). Similarly, polygenic risk scores for CD and general IBD correlated to differences in community composition and degree of dysbiosis (CD-PRS/MD: ρ = 0.0834, *p* = 0.0007; IBD-PRS/MD: ρ = 0.0694, *p* = 0.0046; Spearman correlation; P_CD-PRS_ = 0.0002; P_IBD-PRS_ = 0.0005; PERMANOVA), while UC-PRS showed only weak associations (Figure S2B & S2C; UC-PRS/MD: ρ = 0.0107, *p* = 0.6637; P_UC-PRS_ = 0.0257).

IBD pathology was consistently the most influential factor in the microbial community analyses across time points (Figure 3(J), Figure S9; Table S16 &amp; Table S21). However, the presence of a stoma/artificial bowel outlet (colostomy, ileostomy, permanent/temporary stoma) or other pathologies that influence the gut transition time (obstipation, diarrhea, intestinal stenosis) strongly correlated with the composition of the microbial communities which is in line with the prominent effect of BSS in our cohort (Figure 3(J)). This pattern remained consistent even after excluding potential confounding effects such as age, BMI, sex, and IBD pathology across time points (see Table S21).

In addition to the impact of different pathologies, we observed a significant impact of various pharmaceutical treatments on the microbial community composition. Interestingly, antibiotic treatments (up to one year prior) had significant but no prominent effect on the community composition, as reported before.^30^ However, the most influential drugs in all three sampling time points were those influencing fecal passage time/stool consistency like antidiarrhetics (*e.g*. loperamide; Figure S10C). This finding reinforces the strong and consistent association of fecal passage time and its derivatives (*i.e*. BSS) with community composition and persisted after correcting for IBD condition, age, BMI, and sex (Figure S9 &amp; S10, Table S22). As expected individuals taking these drugs had softer stools (BL: *W* = 24756, *p* = 3.3910 × 10^−9^; Wilcoxon-test).

Diet is another important factor influencing the microbial communities. Intake of eicosatetraenoic acid (F20:4/arachidonic acid) was correlated to increasing dysbiosis at baseline and follow-up time points (Figure S9G-I, S10D; Table S23). In contrast, the uptake of vitamins, trace minerals and long-chain carbohydrates (*e.g*. ZB-Fiber) correlated with less disturbed community compositions.

### An uncharacteristic community cluster is associated with dysbiosis and clinical signs of inflammation

**Figure 4.**
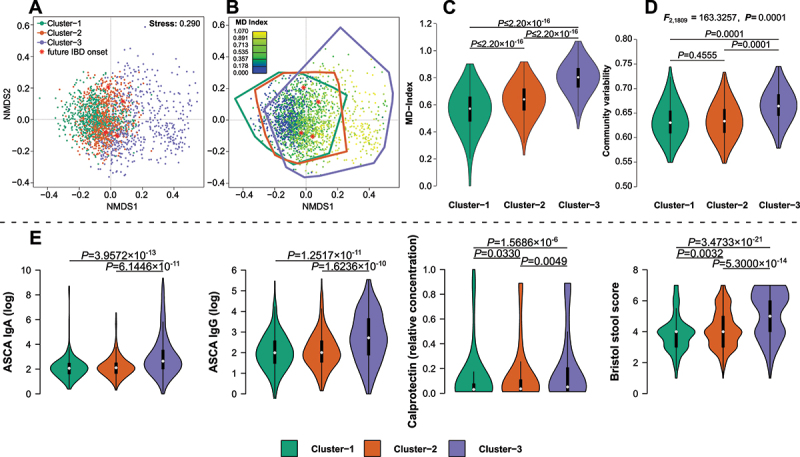
(A) Community clusters of the microbial community at the baseline time point, determined by dirichlet multinomial mixture modelling (DMM) and optimal clustering was determined via Laplace goodness of fit optimization. (B) Overlay of the microbial dysbiosis index gradient (Gevers et al.^29^) and community clusters (outlines), including healthy onset patients at baseline (indicated by *). (C) Community clusters display a significantly elevated level of dysbiosis in clusters 2 and 3 (pairwise Wilcoxon tests), (D) as well as elevated levels of community variability in cluster-3 (PERMANOVA). (E) Violinplots visualize the differences of inflammation related physiological variables between community clusters, highlighting elevated levels of inflammatory biomarkers in cluster-3 (pairwise Wilcoxon test).

Unsupervised community clustering of baseline samples revealed three community clusters, which partially overlap with previously reported clusters, sharing some typical indicators (Figure 4(A)).^31,32^ Cluster-1 is characterized by a high abundance of *Prevotella* and *Ruminococcaceae* (*Prevotella*: *P*_*FDR*_ = 1.1162 × 10^−26^; *Ruminococcus*: *P*_*FDR*_ = 2.1846 × 10^−59^; Kruskal test), while cluster-2 shows a high abundance of *Bacteroides* and *Faecalibacterium* (*Bacteroides*: *P*_*FDR*_ = 1.4604 × 10^−17^; *Faecalibacterium*: *P*_*FDR*_ = 1.5310 × 10^−84^; Kruskal test). In contrast, cluster-3 was characterized by a higher abundance of *Enterobacteriaceae* (*e.g. Escherichia/Shigella*: *P*_*FDR*_ = 4.0936 × 10^−61^; Kruskal test), which is unconventional for so-called “enterotypes” and persisted after the inclusion of follow-up samples in the clustering (two DMM clusters remain; see Figure S13). Individuals in cluster-3 showed a greater potential for inflammation as indicated by the elevated levels of dysbiosis (*p* < 2.2 × 10^−16^; Kruskal test, Figure 4(B,C)), community variability (Figure 4(D)), and a higher proportion of IBD patients (Figure S13). Furthermore, cluster-3 displayed increased levels of inflammatory markers such as ASCA IgG & IgA, or increased stool softness/BSS compared to the remaining clusters (Figure 4(E)). We also observed a significantly higher level of CD-PRS (*F*_2,1665_ = 12.5869, *p* = 3.7541 × 10^−6^; ANOVA) and general IBD-PRS (*F*_2,1665_ = 4.3156, *p* = 0.0135; ANOVA) among individuals in this unconventional community cluster (Figure S13). However, a comparable community type has been reported before and corresponded to a dysbiotic community composition, positioned outside the more frequently observed community states detected so far.^33–35^ This implied a distinct community state with potential connections to broad host genetic characteristics and proinflammatory responses.

### IBD onset drives variable physiological responses and limited microbiome changes

**Figure 5.**
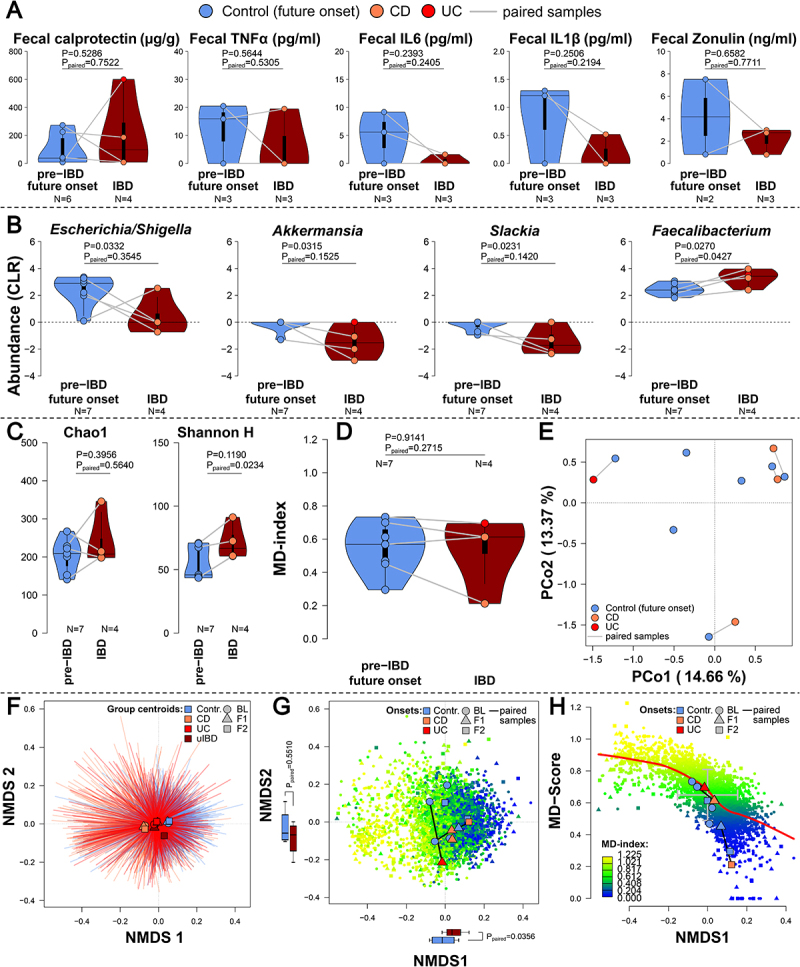
(A) Analyses of selected physiological fecal inflammation markers (fecal calprotectin, TNFα, IL6, IL1β, Zonulin) with respect to pre- and post-diagnosis in onset patients (ANOVA-P value, paired *t*-test-P_paired_ value) show no significant differences between disease states. Individuals with paired samples (pre- and post- onset/diagnosis) are connected by lines (*N*=4). (B) the violin plots display nominally significant differentially abundant genera, between pre- and post-diagnosis onset patients (ANOVA-*P* value, paired t-test-*P*_*paired*_ value), as well as (C) increasing Shannon diversity in post-diagnosis onset patients, insignificantly elevated Chao1 levels and (D) dysbiosis (MD-index^35^). (E) Principle coordinate analysis of Bray-Curtis distances between onset patients pre- and post-diagnosis (N_Contr._=7, N_CD_=3, N_UC_=1). Individuals with paired samples (pre- and post- onset/diagnosis) are connected by lines (*N*=4). (F) non-metric multidimensional scaling (NMDS) of Bray-Curtis distances between all samples across all time points (BL, F1, F2), displaying the significant clustering by health conditions (*F*_2,2951_=6.77216, *p*<0.00010, *R*^*2*^=0.00469, *adj. R*^*2*^=0.00389, PERMANOVA, excluding uIBD) and (G) the gradient of community dysbiosis as expressed by MD-index. The NMDS further highlights onset cases before and after disease manifestations and shows directly paired samples as connected. The marginal boxplots display differences between NMDS scores of these paired onset samples (paired *t*-test). (H) Correlation of the MD-index and the first NMDS axis showing a clear gradient of dysbiosis in the community composition (*ρ*=-0.7569, *p*<2.20 × 10^−16^; Spearman rank correlation) and the distribution of onset cases around the mean of the community distribution (NMDS1). P-values were not adjusted for multiple testing.

With special focus on the onset cases in our cohort, healthy individuals who developed IBD during the study, we investigated the physiological and microbial changes during the transition from the preclinical state (healthy future onset, *N* = 7) to IBD manifestation and diagnosis (*N* = 4), as well as their general characteristics. Focusing on physiological parameters available for future onset patients, we identified insignificantly lower levels of fecal calprotectin before disease manifestation than after (Figure 5(A)). In contrast future onset patients showed insignificantly elevated levels of fecal/intestinal TNFα, IL1β, IL6, and Zonulin. In the fecal microbiome, we observed a reduced abundance of *Escherichia/Shigella*, *Akkermansia*, and *Slackia* after disease manifestation, while the abundance of *Faecalibacterium* increased significantly (Figure 5(B)). Community alpha diversity in onset patients increased after IBD manifestation, as based on Shannon diversity but not species richness (Figure 5(C)). Compared to the whole cohort, the alpha diversity of healthy future onset patients did not differ from either healthy controls or IBD patients (Figure 2(D), Figure S8). An analysis of community composition revealed no clear clustering of pre- and post onset samples (Figure 5E) or significant changes of the dysbiosis level (Figure 5(D)). In the context of the whole cohort, healthy future onset patients were mostly distributed around the center of the community compositional distribution and dysbiosis gradient (Figure 5(G,H)). However, future onset patients displayed a directional shift after disease manifestations in the direction of less dysbiotic compositions along NMDS1 (*t* = −3.5258, df = 3, *p* = 0.03876; paired *t*-test; Figure 5(F–H)).

In summary, future onset cases did not yet represent community outliers or dysbiotic community states prior to disease manifestation. Disease onset led to slight changes in alpha diversity and abundances of a small number of bacterial taxa. In this limited set of onset patients, we did not observe significantly elevated markers of inflammation after IBD manifestation, but rather individualized community alterations and trajectories.

### Network analyses show systemic and transferrable patterns of community disturbance and disease dependent taxon centrality in community networks

**Figure 6.**
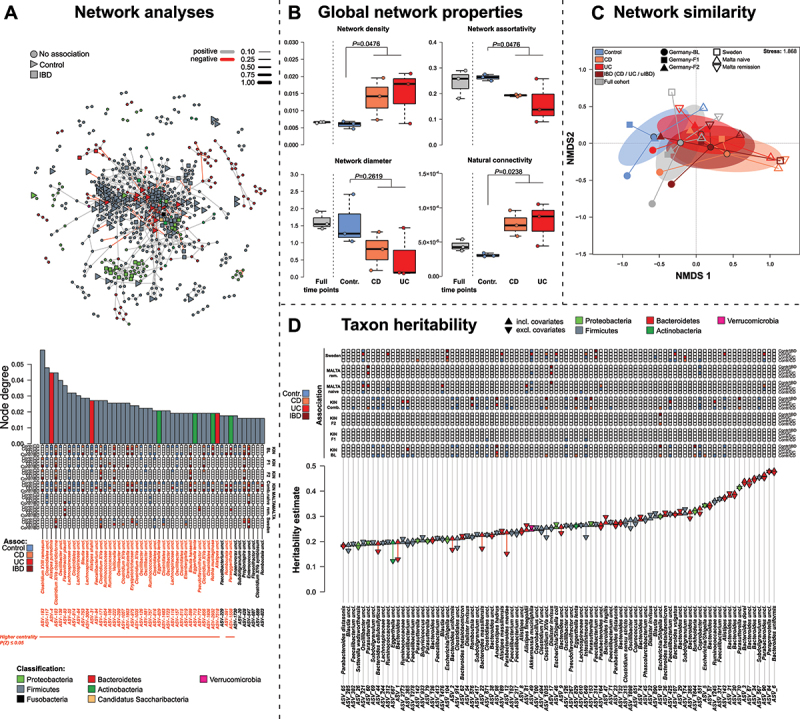
(A) Spiec-Easi networks of baseline samples (N_BL_=1812). Bacterial nodes highlight significant differentially abundant ASVs in the network.^36^ Bacteria not showing any differential abundance patterns between IBD patients and healthy controls are signified via (●), bacteria overabundant in IBD (combined CD, UC, uIBD) via (■) and bacteria more abundant in controls are signified via (►). The barplot visualizes node centrality based on the number of connections (node degree) at the baseline time point (see Figure S14-S16 and Table S24 for BL, F1, and F2). Colored boxes highlight corresponding ASV differential abundance patterns in KINDRED and the external cohorts. Significance of centralities is derived from Z-tests against a randomized networks (10’000) and nodes with significantly higher degrees than expected by chance are. (B) Global network characteristics were derived from networks constructed from the disease condition-specific networks of each sampling timepoint, which are informative for stability and structure of the respective networks (Wilcoxon-test healthy vs. CD/UC). Network metrics include centrality based assortativity,^37^ network diameter, radius, and size as well as density/clustering,^38,39^ and natural connectivity.^40^ (C) Network similarity of disease and time point-specific subnetworks, as well as networks derived from the external cohorts (Malta, Sweden) based on graphlet distance^41^ and displayed via NMDS (see Materials and Methods section). Networks display compositional differences between healthy and diseased networks (Control *vs*. IBD (incl. IBD networks): *F*_1,18_=2.4144, *p*=0.0412, *R*^*2*^=0.1183, *adj. R*^*2*^=0.0693; PERMANOVA). (D) Heritability estimates derived from the likelihood based method *lme4qtl*^42^ using either only kinship information with or without additional environmental and anthropometric covariates (▲-incl. environmental covariates (*h*^*2*^_*full*_), ▼-no covariates (*h*^*2*^_*null*_)). Only the upper quartile of taxa are highlighted (based on *h*^*2*^_*full*_ estimate including environmental covariates). Additional information like differential abundance in IBD accross cohorts are depicted for each taxon (Figure S19, Table S25).

To analyze changes of interactions among microbial community members in IBD, we constructed co-abundance networks to identify important taxa and patterns in the fecal microbial communities that may be central for community and host homeostasis (Figure 6(A), Figure S14-S17). Across networks (split by time point and pathology), we observed differences in the composition and topography of networks constructed from the healthy communities and networks constructed from the different IBD subgroups of the KINDRED cohort (control *vs*. IBD: *F*_1,7_ = 2.4873, *p* = 0.0947, *R*^*2*^ = 0.2622, *adj. R*^*2*^ = 0.1568, PERMANOVA Graphlet distance; see Figure 5(C)). This pattern became more clear when networks derived from the external cohorts (Malta untreated, Malta remission, Sweden-SIC) cohorts were included (Control *vs*. IBD (incl. general IBD networks): *F*_1,18_ = 2.4144, *p* = 0.0412, *R*^*2*^ = 0.1183, *adj. R*^*2*^ = 0.0693, PERMANOVA Graphlet distance; Figure S18A). Furthermore, we observed signs of network/community contraction (increasing network density and radius) and increasing resistance to change, as implied by increased “natural connectivity” (Figure 6(B) & S18B).^40,43^

At the single taxon level/node level (Figure 5(A), Figure S14-S17), we observed various IBD associated groups consistently occupying important network positions across time points (*Clostridium XVIII ramosum* (ASV-193), *Clostridium XlVa clostridioforme/spec*. (*e.g*. ASV-103, ASV-171)). Also potential oral bacteria (*e.g. Veillonella, Rothia, Cand. Saccharibacteria, Fusobacterium)* and members of the *Enterobacteriaceae* (*e.g. Klebsiella*), showed elevated network importance, particularly in IBD derived networks. Similarly, *Flavonifractor plautii* (ASV-93) or the Actinobacterium *Eggerthella lenta* (ASV-713) are IBD associated^44,45^ and located in central positions in IBD associated networks (Figure S14-S17, Table S24). In contrast, ASVs belonging to the *Alistipes* are highly central and consistently abundant in control individuals across time points and external cohorts, like other SCFA producing bacteria (*e.g. Barnesiella, Lachnospiracea: Clostridium XlVb uncl*., *Clostridial cluster IV*, *Ruminococcus;* Figure 5(A), Figure S14-S17, Table S24). However, due to the statistical nature of network construction, centralities and key-stone status may vary.^46,47^

### Heritability of microbial taxa

Using a mixed model framework, we investigated the heritability of single ASVs using pedigree relationships in the cohort, based on the large baseline cohort at a prevalence cutoff of 10%. Most of these associations were driven not only by host kinship or family relationship (*h*^*2*^_*null*_), but also by environmental/anthropometric/lifestyle characteristics (age, sex, BMI, and IBD status; *h*^*2*^_*full*_; Figure 5(D), Figure S19; Table S25). At the ASV level members of the *Bacteroidales*, particularly *Bacteroides uniformis* (ASV-6, *h*^*2*^_*null*_ = 0.4723) and *Paraprevotella uncl*. (ASV 84, *h*^*2*^_*full*_ = 0.4783) displayed the highest heritability estimates (Table S25). The majority of taxa with high heritability (top 25%) were more abundant in healthy individuals, or not associated to IBD (*e.g. Feacalibacteria uncl., A. muciniphila, B. thetaiotaomicron, Alistipes uncl*.) (Table S25). Only a small number of disease-associated taxa displayed high heritability such as *Escherichia/Shigella* (ASV-5, ASV-10; Proteobacteria) or the oral bacterium *Dialister invisus* (ASV-17; Firmicutes). The community wide dysbiosis indices (MD-index), showed moderate heritability, which implied moderate genetic influence on, or intra-family transmission, of dysbiosis. The heritability of the MD-index was relatively low (*h*^*2*^_*full*_ = 0.0813) as compared to the average ASV heritability (*h*^*2*^_*average*_ = 0.1064, *h*^*2*^_*max.*_ = 0.4783, *h*^*2*^_*min.*_ = 0.0000). However, we did not observe a direct influence of relatedness on the level of dysbiosis among FDRs of IBD patients. We only detected elevated levels of the MD-index in affected individuals compared to healthy family members, while healthy FDRs showed similar levels of MD-index to more distantly related or unrelated individuals.

## Discussion

The prospective KINDRED cohort study provides new insights into the evolution and state of IBD by integrating longitudinal multiomic data – including microbial, genetic, and clinical parameters – collected from patients and their family members.^14,18,48,49^

### Oralization and enterobacteriaceae overabundance as consistent IBD features

A strong characteristic microbial pattern in IBD, particularly for CD, arises from the highly consistent association of oral bacteria in IBD, as seen in our analyses. Various taxa across different phyla with an potentially oral origin show clear associations with IBD across studies and cohorts (*e.g. Fusobacteria, Klebsiella*).^50–52^ Oral *Fusobacteria (e.g. F. nucleatum*) and their cell components have been proposed as causal triggers of inflammation,^53,54^ and colorectal cancer.^28^ *Cand. Saccharibacteria* (formerly TM7) are a rarely described group of obligate oral epibionts, which are correlated to the IBD-related microbial dysbiosis and oralization in this cohort and have been detected before in the context of IBD.^27,55^ Interestingly, the only oral taxa with beneficial associations in the intestine belonged to the genus *Dialister*, probably due to its regular and not disease associated translocation to the intestine which occurs also under normal physiological conditions.^51^ Furthermore, these taxa also became more central in disease-associated community networks which speaks for a stable colonization and ecological impact on the community. These observations point to detrimental physiological alterations and a potential loss of colonization resistance of the intestinal community in IBD,^56^ as oral colonization is seemingly absent in healthy individuals.^51^

Various *Enterobacteriaceae* are known to elicit and exploit inflammatory processes and are associated with the development of IBD^50^ at the ecological,^57^ metabolic,^58^ or immunological level.^18,59^ *Enterobacteriaceae*, including *Klebsiella* and *Escherichia/Shigella*, were strongly associated with IBD in our cohort and across external validation cohorts.^50,52,56^ Their abundance correlated with clinical and genetic markers of disease activity (*i.e*. ASCA, calprotectin, BSS, IBD-PRS).^18,59^ *Clostridium clostridioforme* (*Clostridium XIVa*) was also specifically linked to Crohn’s disease.^48,60,61^

The compositional differences between IBD pathologies and healthy controls resemble previously observed patterns of IBD associated microbial communities.^29,50,52^ These compositional changes, including the emergence of an *Enterobacteriaceae*-dominated cluster, underscore the profound community turnover and dysbiosis in IBD.^35^ Although this *Enterobacteriaceae* community cluster was rarely observed before, it has been linked to dysbiotic community states and showed associations to different pathologies.^33–35,50^

We observed increased inter-individual variability in IBD-associated microbiomes. This phenomenon has been called “Anna Karenina principle”^62^ in which diseased communities are more dissimilar from each other than are healthy communities. This increased “community dispersion” may reflect greater stochasticity and instability in IBD but its significance has rarely been discussed.^18,55,62–64^

Notably, individuals who developed IBD during follow-up (onset cases) did not initially display outlier or highly dysbiotic communities. However, the communities show a surprising shift toward a less dysbiotic community composition after disease development, which might be the result of elevated community variability through external and internal drivers, *i.e*. “Anna Karenina principle.”^62^ We also observed increased abundances of *Faecalibacteria* after IBD manifestation. This pattern was observed in previous studies and is potentially driven by *F. prausnitzii* phylotypes able to temporarily multiply in the inflamed gut.^55,65,66^ Interestingly a previous study observed increased abundances *Faecalibacteria* already in pre-IBD individuals, which was important predictive feature for IBD.^67^ However, the small sample size of onset cases (*N* = 7), particularly with paired samples pre- and post-diagnosis (*N* = 4), should be taken into account.

These findings suggest that IBD is characterized by persistent, large-scale changes in community structure and diversity, increased susceptibility to colonization by oral and pro-inflammatory taxa, and altered community dynamics.^43,64^ Furthermore, the IBD-associated communities appear to be more susceptibile to colonization by oral and proinflammatory taxa, which implies a reduced colonization resistance of the resident communities and the existence of adequate aerobe niches^56^ which eventually negatively influence the population of beneficial anaerobe taxa.^18,29^

### Physiological markers of inflammation are strongly disease specific and correlate with the the microbiome

Decreased stool consistency, an indicator of reduced intestinal transit time and even diarrhea, are hallmarks of IBD pathology.^68^ Stool consistency and gut transit time emerged as major determinants of microbial composition, with softer stools and reduced transit time associated with increased dysbiosis and loss of diversity – especially in IBD patients. Changes in transit time can change the taxonomic-, functional-, and metabolite composition by transitioning from saccharolytic to proteolytic metabolism and can even influence bacterial growth rates and load.^69–71^ These effects may even surpass dietary and disease-related signals^69,72^ and are repeated at taxon level (Bacteroidetes- lower BSS). These patterns were significantly exaggerated in IBD patients, which implies a greater impact of passage time/stool consistency on bacteria in IBD patients or vice versa. Furthermore, among the most influential pharmaceutical treatments and medical conditions on the microbial communities in our cohort also affected fecal flow (*e.g*. stenoses, antidiarrhetics). Antidiarrhetics can have growth limiting effects on intestinal microbes, besides their effects on fecal flow and the host.^30,73^

The two main IBD pathologies, CD and UC, are physiologically and anatomically quite distinct. We identified strong disease-specific patterns in ASCA antibodies, GP2 antibodies, and fecal calprotectin, as expected.^74^ Furthermore, they strongly correlated with microbiome composition, diversity, level of dysbiosis, and single bacterial taxa, particularly in IBD patients. However, bacterial taxa that associated to ASCA Ig levels overlapped only in parts with recent IBD studies (*e.g. Ruminococcaceae*).^75^ The interaction between the microbiome and the immune system may directly or indirectly drive the antibody responses, yet it is still unclear whether they represent cause or consequence of IBD.^74–76^

### Interaction between genetic risk and microbial characteristics of dysbiosis in IBD

Polygenic risk scores integrate the abundance and penetrance of known risk variants for diseases such as IBD into a single genome-wide risk score. Although PRS effectively capture established IBD variants, these scores have their limitations particularly with cohorts of non-European ancestries,^77^ a combination of host and microbiome derived risk scores may have great potential in chronic diseases.^78^

In particular, ASCA IgG and IgA and GP2 IgA have strong positive associations to CD-PRS and general IBD-PRS and were described as strong biomarkers for IBD.^21,23,74^ In contrast to recent studies we showed a significant positive association of ASCA Ig levels with polygenic risk scores for IBD, particularly for CD.^76,79^ ASCA Ig levels have not been shown to be associated to high risk genetic markers of CD (*CARD15*/*NOD2*), but rather their responsiveness to environmental signals, including bacterial antigens, might be genetically determined.^76^

Naturally, PRS for IBDs are higher in IBD cases than in controls, however the significantly elevated risk level among IBD onset patients^80^ further emphasized the potential of these scores to stratify individuals at risk. However, the relatively weak association of UC-PRS with microbial community characteristics, compared to CD-PRS and IBD-PRS points to a reduced importance of host-microbe interactions and/or lower penetrance of associated risk variants in UC.^81^ In alignment with this, previous studies found comparatively weaker and fewer associations with microbiome characteristics like taxon abundances or diversity in UC^75,82^ compared to CD.^55^

### Mainly commensal bacteria show considerable heritability

Broad genetic patterns, as determined by kinship patterns, influenced the taxonomic composition of the bacterial communities as previously shown^83^ and may add to the transfer of inflammation and dysbiosis within families.^84–87^ However, based on our heritability estimates of dysbiosis this transfer of potentially pathological microbial patterns was only moderate (*h*^*2*^ = 0.08) and distinctly lower than estimates previously reported by Turpin *et al*. 2016 for the MD-index (*h*^*2*^ = 0.2728).^88^ In contrast, mainly commensal bacteria showed noteworthy heritability in our cohort, comparable to a recent multi cohort study.^89^ In particular, the ubiquitous human gut commensal *Bacteroides uniformis*,^90^ among other *Bacteroides* species, showed the strongest association with host relatedness/kinship as previously shown^89,91,92^ in contrast to earlier twin studies.^83^

## Conclusions

The central aim of the KINDRED cohort is to provide a resource for identifying lifestyle factors and biomarkers associated with the disease course and onset in individuals with IBD on a multiomic level, integrating, for example, genetics, and microbial information. In summary, our longitudinal analysis of the KINDRED cohort reveals that IBD is associated with reproducible gradients of microbial dysbiosis, loss of diversity, overabundance of oral and pro-inflammatory taxa, and increased inter-individual variability. These changes are linked to both host genetics and disease-specific physiological markers, but heritability analyses suggest a dominant role for environmental factors in shaping disease-associated microbiome alterations.

## Limitations

Certain limitations are present in this study, such as healthy relatives were not examined in an examination center but rather did they self-report. Also, IBD status was not established by one center, but was validated based on self-reports and practitioner questionnaires, and on provided medical records. Another potential weakness of the KINDRED cohort is its open and long-term design, with only a slow increase of onset cases, which limits the analysis of biomarkers for IBD onset. Our presented analysis of the onset patients is underpowered; however, it provides limited ,yet valuable, insights into the microbial changes during disease manifestation as a proof-of-concept. Moreover, the reliance on 16S rRNA-based community analyses in this study, rather than on metagenomic, metatranscriptomic, or culturomic data, limits our perspective, but ongoing data collection and multiomic analyses will address these limitations in future studies.

## Materials and methods

### Basic study design and cohort

The KINDRED cohort is a German-wide, prospective cohort study that collected both, questionnaire data and biomaterials from IBD patients and their (affected and unaffected) family members. Despite great efforts to include as many family members as possible, a focus has always been on unaffected individuals with a family history of IBD. The recruitment of study participants started in October 2013 and is still ongoing (state 2025). Follow-up information and new biomaterial samples were collected prospectively at intervals of approximately two years (Figure S21-S22). As of April 2021, the IBD Family Cohort has thus enrolled 1497 IBD patients together with 1813 (initially) non-affected first- or second-degree relatives from Germany (minimum age at inclusion: 7 years). Participants, including the IBD patients, were asked to provide questionnaire data and biomaterials at baseline and after every 2 years of follow-up (Table S1 &amp; S2). The resulting patient counts are shown in Table 1 and Figure 1(A). In addition, physician-administered questionnaires and medical records were collected from IBD patients to obtain physician-validated information, such as diagnosis, disease pattern/location, activity, and medication and validated the diagnoses, if possible (see Supplemental Methods and Table S1). Using these data, the Kiel IBD Family Cohort aims to facilitate the comprehensive molecular, clinical, lifestyle, nutritional, and sociodemographic characterization of patients and high-risk individuals, and to identify preclinical signs for the onset of IBD. Additional information on eligibility criteria, enrollment, data and biomaterial collection, as well as data management, and privacy protection is given in the Supplement of this article.

A standard battery of questionnaires was used to assess self-reported dietary behavior (12-month recall questionnaire) and metrics of well-being and quality of life (quality of life index, Fatigue Severity Scale index), were also employed (see Supplemental Methods).^93–95^ Nutritional data were adjusted by total energy consumption to decouple caloric consumption from diet composition. Individuals with an undefined or unclassified IBD diagnosis, but with intestinal inflammation (e.g. suspected CD/UC, colitis) are summarized under the category unclassified IBD (uIBD, Table S3). See Supplemental Methods for additional information on cohort design, recruitment, and cohort maintenance.

### Ethics and human samples

The KINDRED cohort study protocol was approved by the ethics committee of the Medical Faculty of Kiel University (AZ A117/13). Every study participant provided written informed consent on forms that were age-adapted. For participants under the age of 18 years, informed consent must also be signed by their parents. When the participants reached the ages of 12 and 18 years a new informed consent (reconsent) form was signed by the participants themselves and, in the case of 12-year-old adolescents, by their parents.

A cohort of treatment naïve and newly diagnosed IBD patients in an active disease state from Malta (naïve: N_CD_ = 31, N_UC_ = 25), including a healthy control cohort (N_Contr._ = 96), was recruited as described elsewhere.^55^ In addition we included a Maltese patient cohort currently in disease remission and treatment (remission: N_CD_ = 32, N_UC_ = 66), as described recently.^64^

Individuals from the Swedish Inception Cohort in IBD (SIC-IBD)^96^ were included as treatment-naïve patients, between 20–77 years of age. Symptoms, such as diarrhea, abdominal pain, bloody or mucous stools for > 2 weeks, were inclusion criteria. The final diagnosis of IBD was established according to internationally accepted criteria, including clinical, microbiological, endoscopic, histological, and radiological evaluation (N_CD_ = 17, N_UC_ = 16). Patients with gastrointestinal symptoms but without endoscopic and histological signs of IBD-associated inflammation were considered symptomatic controls (SC, N_SC_ = 16). In addition, 17 healthy individuals were included as healthy controls (N_contr._ = 16, one failed sequencing).

### Physiological measurements

Calprotectin (indicator of intestinal inflammation) was measured in fecal samples using a Bühlmann fCAL™ ELISA kit under low-range conditions (BÜHLMANN LABORATORIES AG; range 10–600 μg/g) and analyzed using SoftMax Pro Software (Molecular Devices). ASCA IgA/IgG and GP2 IgA/IgG serum levels were measured externally using ELISA (Medipan GmbH) including batchwise calibration samples. Occult fecal blood was determined by PreventID® Haemo/HaptOccult (Preventis GmbH, Bensheim, Germany). Other immunological measures (CRP, Hb) were obtained during the initial examination using standard clinical tests. Stool levels of Zonulin – were measured as to manufacturer’s specifications (IDK® Zonulin, Stool ELISA Kit) as were fecal IL1b (Calex-Tube, Invitrogen IL-1b Human ELISA Kit), IL6 (Calex-Tube, Invitrogen IL-6 Human ELISA Kit), and TNF-alpha (Calex-Tube, Invitrogen TNF alpha Human ELISA Kit).

### Polygenic risk scores

GSA data were quality controlled via gwas-qc (https://github.com/ikmb/gwas-qc) not correcting for closely related individuals in the cohort using hg19 (genome build 37) of the human genome and 1000 Genome reference set. Potential sample mix-ups and unclear relatedness patterns were manually checked and corrected if needed. Imputation was performed after chromosome-wise transformation into bgzip VCF files via plink2.^97^ Single VCF files were uploaded to hybridcomputing.ikmb.uni-kiel.de/webservice/sites/., imputed and phased via EagleImp (Genome build: GRCh37/hg19, Reference: 1000 Genomes Phase 3, r^2^ filter: 0.1, allowed reference swaps, and strand flips).^98^

To examine the genetic susceptibility of individuals in the KINDRED cohort for IBD and its subtypes CD and UC we calculated Polygenic Risk Scores (PRS). GWAS summary statistics were taken from the meta-analysis of IBD from Liu et al .^99^ The summary statistics are based on a total of 5956 CD and 6968 UC patients with an additional 21,770 population of controls with European ancestry. PRS were calculated with *LDpred2* within the *bigsnpr* (v. 1.12.2) R package.^24,100^ After quality control of the summary statistics^101^ the method calculates a posterior mean effect size based on linkage disequilibrium information and base effect size for all remaining available markers in both data sets. We used the *auto*-method of *LDpred2*, which automatically estimated the parameter sparsity *p* and the SNP heritability *h*^*2*^ and did not require to tune hyper-parameters in the validation set.

### Stool sample processing

DNA extraction, sequencing and bioinformatics processing of 16S rRNA gene libraries from stool samples were performed as described previously in detail.^102^

### Data processing

Data processing of 16S sequences was performed using DADA2 1.10^103^ via the workflow for big datasets (https://benjjneb.github.io/dada2/bigdata.html., https://github.com/mruehlemann/ikmb_amplicon_processing/blob/master/dada2_16S_workow_with_AR.R) resulting in abundance tables of Amplicon Sequence Variants (ASVs). All sequencing runs were handled separately for error correction, read merging, and combined chimera detection. ASVs underwent taxonomic annotation using the naïve Bayesian classifier implemented in DADA2 using the Ribosomal Database Project 16 release.^104,105^

### Statistical methods

#### Alpha diversity

Species richness (Chao1), Shannon diversity (numbers equivalent) were calculated and analyzed in R 3.5.3.17–19. The relationships of physiological- and microbial inflammation markers/indices with alpha diversity were analyzed using linear models after correcting for relevant covariates (LM: residuals(variable~gender+scaled BMI + scaled Age) ~ IBD (Contr., CD, UC) * physiological variable). Model fits were visualized via *base* R and *jTools*.^106^ General correlation of MD-index with alpha diversity was tested via simple linear models, segmented linear models (*segmented* v2.1–3),^107^ or polynomial linear models and assessed via the AIC.

#### Beta diversity

Analyses were conducted via distance based (conditional) Redundancy analyses and permutative ANOVA, as well as with a multivariate test for homogeneity of variances (10000 permutations)^108,109^ using Bray-Curtis dissimilarity (differential abundance). Global and pairwise differences in community variability were assessed via permutation test of multivariate homogeneity of group dispersions (10’000 permutations, via the *betadisper* function). Community clustering according to anthropometric, community distances of naïve and rarefied communities were highly correlated and thus higher coverage naïve samples were used for analysis (Bray-Curtis: Mantel: *r* = 0.9588, *p* < 0.001; Procrustes: m^2^12 = 0.06012, *r* = 0.9695, *p* < 0.001; Jaccard: Mantel: *r* = 0.9941, *p* < 0.001; Procrustes: m^2^12 = 0.006143, *r* = 0.9969, *p* < 0.001).

#### DMM community clustering

Community clustering was performed via Dirichlet Multinomial Mixture modeling as implemented in the R packages microbiome (*microbiome_1.20.0*)^110^ and DirichletMultinomial (*DirichletMultinomial_1.40.0*)^111^ after centered log ratio transformation (CLR) implemented in *compositions*.^112^ The optimal number of clusters was determined via Laplace goodness of fit optimization in a range of 1–15 clusters.

#### Differential abundance and correlation analysis

Taxon abundances were filtered by having normalized counts of at least 5 among 1% of samples (*DESeq2* median of ratios transformation). Negative binomial GLMs with Wald tests as implemented in *DESeq2* (including automated effect filtering) were employed to detect differentially abundant taxa for each time point separately as well as for all time points combined.^113^ To reduce the effect of potential confounding effects we included subject age, BMI and sex as covariates and in addition the time point itself, for the combined dataset. P-values were adjusted via FDR and only comparisons excluding the small subset of uIBD patients were investigated further (Contr./CD, Contr./UC, CD/UC, Contr./IBD).

Models:

$$\mathrm{Abundance}∼\;\mathrm{sex}+\;\mathrm{BMI}\;\left(\mathrm{centered}-\mathrm{scaled}\right)\;+\;\mathrm{age}\;\left(\mathrm{centered}-\mathrm{scaled}\right)\;\left[+\;\mathrm{time}\;\mathrm{point}\;\left(\mathrm{BL}/F1/F2\right)\right]\;+\;\mathrm{IBD}\;\mathrm{pathology}\;\left(\mathrm{Control},\;\mathrm{CD},\;\mathrm{UC},\;\mathrm{uIBD}\right)$$

$$\mathrm{Abundance}∼\;\mathrm{sex}+\;\mathrm{BMI}\;\left(\mathrm{centered}-\mathrm{scaled}\right)\;+\;\mathrm{age}\;\left(\mathrm{centered}-\mathrm{scaled}\right)\;[+\;\mathrm{time}\;\mathrm{point}\;\left(\mathrm{BL}/F1/F2\right)\;\text{\textbackslash{}break}\;\;\;\;\;\;\;\;\;\;\;\;\;\;\;\;\;\;\;\;\;\;\;\;\;\;\;\;\;\;\;\;\;\;\;\;\;\;\;\;\;\;\;\;\;\;\;\;\;\;\;\;\;\;\;\;\;\;\;\;\;\;\;\;\;\;\;\;\;\;\;\;\;\;\;\;\;\;\;\;\;\;\;\;\;\;\;\;\;\;\;\;\;\;\;\;\;\;\;\;\;\;\;\;\;\;\;\;\;\;+\;\mathrm{Health}\;\mathrm{status}\;(\mathrm{Control}/\mathrm{IBD}\;[\mathrm{CD},\;\mathrm{UC},\;\mathrm{uIBD}])$$

Taxon abundances were correlated with a reduced set of clinical variables (ASCA IgA, ASCA IgG, GP2 IgA, GP2 IgG, fecal calprotrectin, BSS) as well as polygenic risk scores for IBD in general and for CD or UC in particular. CLR transformed species abundances were correlated with core physiological measures via *ppcor*,^25^ combining the P-values of Spearman-, Kendall-, and Pearson correlations via Brown’s method to detect and incorporate the most consistent associations across different association measures.^26^ The resulting P-values were FDR corrected and Spearman *ρ* was used for visualization via *ComplexHeatmap* (v2.14.0).^114^

#### Dysbiosis score

Dysbiosis scores based on the distribution of bacterial community members were calculated following Gevers *et al*. (2014) (Microbial Dysbiosis index/MD-index).^29^ For the MD-index we combined significant taxa abundant in either CD or UC patients (uIBD not included) as compared to healthy/non-IBD cohort members at the baseline time point of the KINDRED cohort and modified the metric to be a positive variable [log1p(sums(abundance CD, abundance UC))/log1p(sums(non-IBD))]. Physiological and microbial inflammation markers/indices were analyzed using linear models including relevant covariates (variable~gender+scaled BMI + scaled Age + IBD (Contr., CD, UC, uIBD)), followed by stepwise model selection to minimize AIC without significant loss of fit, separately for BL, F1, and F2. The correlation of the MD with alpha diversity was assessed with linear and quadratic fits and the best fit was selected via minimal AIC for each time point separately. Model fits were visualized via *base* R and *jTools*.^106^

#### Heritability analyses

To estimate the heritability of single communities in a rather heterogeneous and patchy pedigree with many confounding variables we applied a recently published approach employing linear mixed models (*relatLmer*), as implemented in *lme4qtl* v0.2.2.^42^ Thus, we were able to incorporate fixed effects in our heritability estimates using a kinship matrix based on reported pedigree information (*kinship2* v1.9.6.1)^115^ and CLR transformed taxon abundances derived solely from the baseline cohort at a prevalence cutoff of 10% (351 ASVs). In total, we analyzed *N* = 1389 individuals who provided pedigree information (*N* = 1812) and have no incomplete variables (age, BMI, sex, IBD pathology), including individuals who were the only members of their family in the data set. Evaluating the models with and without covariates (AIC, based on the maximum likelihood fit) allows us to further evaluate whether taxon heritability/family association is influenced significantly by environmental factors or family relations alone. To assess patterns among close relatives we categorized relatedness patterns for comparison (related ( > 1st degree), first degree (sibling, parent), unrelated individuals) to investigate average differences between the respective relatedness categories.

#### Network analyses

Networks were generated individually for healthy and IBD (UC, CD) cohort subsets, as well as for the complete time point subsets. The node-based values (degree, betweenness, PageRank-index, eigenvalue-centrality, k-nearest neighbor degree) were calculated in *igraph* v1.2.4.1.^116–118^ Network-wide measures include centrality based assortativity,^37^ network diameter, radius, and size, density/clustering,^38,39^ and natural connectivity.^40^ To assess whether bacteria were more important than expected by chance, observed centralities (mean of control subsamples) were compared against a permuted set of networks (10,000 times) via one-sided Z-tests. Graphlet (4-node) frequency correlation (*orca* v1.1–1, Spearman)^41,119^ and an edge sharing distance (frequency of shared pairwise network interactions) to assess network similarity between the different subgroup networks (KINDRED BL: Controls (*N* = 791), CD (*N* = 551), UC (*N* = 438), IBD (CD, UC, uIBD; *N* = 1021), full time point (*N* = 1812); KINDRED F1: Controls (*N* = 362), CD (*N* = 174), UC (*N* = 105), IBD (CD, UC, uIBD; *N* = 286), full time point (*N* = 648); KINDRED F2: Controls (*N* = 295), CD (*N* = 146), UC (*N* = 92), IBD (CD, UC, uIBD; *N* = 244), full time point (*N* = 539); Malta: Controls (*N* = 96), CD-naive (*N* = 31), CD-remission (*N* = 32), UC-naive (*N* = 31), UC-remission (*N* = 66), IBD-naive (CD, UC; *N* = 56), IBD-remission (CD, UC; *N* = 98), full cohort remission (*N* = 194), full cohort naive (*N* = 152); Sweden-SIC: Controls (*N* = 17), CD-naive (*N* = 17), UC-naive (*N* = 16), IBD-naive (CD, UC; *N* = 33), full cohort (*N* = 66)).

## Supplementary Material

Tables_Supplement_250612.xlsx

250711_Kindred_Cohort_16S_Suppl.docx

## Data Availability

All KINDRED cohort data are stored and managed by the PopGen biobank at the Institute of Epidemiology at Kiel University, Germany. Researchers can apply for data access to the KINDRED data by submitting a research proposal (application ID “2020–012”), including the scientific background, research question, success prospects, study design, potential conclusions, and scientific collaborators of their study, at the local biobank P2N via the following form: http://www.uksh.de/p2n/Information+for+Researchers.html. Due to the informed consent obtained from the participants, phenotypes, as well as genotyping and all 16S rRNA gene-sequencing data, can not be deposited publicly. Raw sequence data and relevant meta-data can be accessed online under the accession number PRJEB44440 (Malta IBD cases in remission), PRJEB47161 (Malta treatment naive IBD cases), PRJEB47162 (Malta controls);^55,64^ and data of the Swedish SIC-IBD inception cohort are available under the accession number PRJEB77933 at the European Nucleotide Archive (https://www.ebi.ac.uk/ena/).

## Abbreviations

CD: Crohn’s disease
IBD: inflammatory bowel disease
IC: informed consent
UC: ulcerative colitis
Uibd: unclassified inflammatory bowel disease
MD: index-Microbiome dysbiosis index
PRS: Polygenic Risk Scores
FDRs: first degree relatives
