# Supplementary material for: First insights into microbial changes within an Inflammatory Bowel Disease Family Cohort study
Source: Gut Microbes. 2025 Oct 5;17(1):2559119. doi: 10.1080/19490976.2025.2559119 (PMC12502818; doi:10.1080/19490976.2025.2559119)
Supplement: 250711_Kindred_Cohort_16S_Suppl.docx [file KGMI_A_2559119_SM0393.docx]

## First Insights into microbial changes within an Inflammatory Bowel Disease Family Cohort study

Philipp Rausch^1^*, Ilka Ratjen^2,3,4^*, Lukas Tittmann^2,3^, Janna Enderle^2,5^, Eike Matthias Wacker^1^, Kathrin Jaeger^2,3^, Malte Christoph Rühlemann^1^, Katrin Franzpötter^2^, Pierre Ellul^6^, Robert Kruse^7^, Jonas Halfvarson^7^, Dirk Roggenbuck^8,9^, David Ellinghaus^1^, Gunnar Jacobs^2,3^, Michael Krawczak^10^, Stefan Schreiber^1,11^, Corinna Bang^1^, Wolfgang Lieb^2,3^*, Andre Franke^1^*

^1^ Institute of Clinical Molecular Biology, University Hospital Schleswig-Holstein, University of Kiel, Germany

^2^ Institute of Epidemiology, University Hospital Schleswig-Holstein, University of Kiel, Germany

^3^ popgen biobank, University Hospital Schleswig-Holstein, University of Kiel, Kiel, Germany

^4^ Department of Internal Medicine II, University Cancer Center Schleswig-Holstein, University Hospital Schleswig-Holstein, Germany

^5^ Institute of Human Nutrition and Food Science, University of Kiel, Germany

^6^ Division of Gastroenterology, Department of Medicine, Mater Dei Hospital, Malta

^7^ Department of Gastroenterology, Faculty of Medicine and Health, Örebro University, Sweden

^8^ Institute of Biotechnology, Faculty Environment and Natural Sciences, Brandenburg University of Technology Cottbus-Senftenberg, Germany

^9^ Medipan/GA Generic Assays GmbH, Ludwig-Erhard-Ring 3, 15827 Dahlewitz, Germany

^10^ Institute of Medical Informatics and Statistics, University Hospital Schleswig-Holstein, University of Kiel, Germany

^11^ Department of Internal Medicine I, University Hospital Schleswig-Holstein, Germany

*denotes equal contribution

**Correspondence: Prof. Dr. Andre Franke**, Christian-Albrechts-University of Kiel, University Hospital Schleswig-Holstein, Institute of Clinical Molecular Biology, Rosalind- Franklin-Str. 12, 24105 Kiel, Germany; [a.franke@ikmb.uni-kiel.de](mailto:a.franke@ikmb.uni-kiel.de)

**Abbreviations used:** CD-Crohn’s disease; IBD-inflammatory bowel disease; IC-informed consent; UC-ulcerative colitis; uIBD-undetermined inflammatory bowel disease, MD-index- Microbiome dysbiosis index; PRS-Polygenic Risk Scores, FDRs-first degree relatives

## Supplemental Methods Study cohort

***Eligibility criteria and enrollment***

The KINDRED includes IBD patients and their first- or second-degree relatives, with a minimum age of 7 years, from all over Germany. Families are deemed eligible if at least one IBD patient and one relative agree to participate. In a few exceptional cases, singleton IBD patients are also included provided that future participation of family members is likely. Most IBD patients are informed and invited into the study by their treating physicians or by IBD patient organizations in Germany, such as the German Crohn’s disease / ulcerative colitis association (DCCV e.V.). The study is also advertised and promoted through a website ([https://www.epi.uni-kiel.de/forschung/ced-studien/familienstudie).](http://www.epi.uni-kiel.de/forschung/ced-studien/familienstudie))

Recruitment into the KINDRED follows a standardized protocol (**Fig. S20**). If patients declare their interest in participating, they are sent a study set and a family documentation sheet. Due to data protection concerns, the study center is not allowed to contact healthy relatives directly without their prior expression of interest in participating. Therefore, every IBD patient is asked to relay details about the study to their family members and to inform the study center through the family sheet about potentially interested candidates. The latter are then formally invited into the study and receive a study set. The study set contains detailed information about the study, an informed consent form, a comprehensive participant questionnaire, and biomaterial tubes for blood, stool, and hair samples. IBD-affected study participants receive an additional questionnaire to be completed by their treating physician. If study sets are not returned within reasonable time, up to three reminders are sent in five-week intervals.

***Follow-up***

Study participants are re-contacted approximately every two years (**Fig. S21**) and asked to fill in a questionnaire with health-related information and to provide new biomaterial samples (blood, stool, hair) at each study follow-up. IBD patients additionally receive a new physician questionnaire. At each follow-up, healthy study participants are asked whether they have been diagnosed with IBD in the meantime. Notably, in general, all initially non-affected participating family members are encouraged to contact the study center immediately after being newly diagnosed with IBD during the course of the study. In this case, the newly diagnosed IBD patient immediately receives a study set, including biomaterial collection tubes and participant and physician questionnaires so as to facilitate the collection of data from very early stages of the disease course (**Fig. S22**).

The first follow-up (2-year follow-up) of the study participants that were recruited in 2013 started in 2015, the second follow-up (4-year follow-up) for this group in 2017, the third follow-up in 2019 (6-year follow-up), and the fourth follow-up of the participants recruited in 2013 started in 2021 (8-year follow-up; 5^th^ data assessment). For participants that were recruited after 2013, the respective follow-up assessments are conducted after the appropriate time intervals.

## Data and biomaterial collection

The KINDRED study prospectively collects biomaterial (blood, stool, hair) as well as comprehensive sociodemographic, socioeconomic, clinical, and lifestyle data from participant questionnaires (adapted to the individual status as IBD patient, healthy relative, and participating child/adolescent; please see below), plus data from physician questionnaires in the case of IBD patients. The study aims to collect complete questionnaire data and biomaterial from each participant but, if a participant refuses to provide some of the data or biomaterial, their incomplete contribution is also accepted.

***Questionnaires***

Separate questionnaires have been developed for healthy family members and IBD patients (for details, see **Table S1**). Both questionnaires have a common backbone of questions related to sociodemographic and socioeconomic characteristics, general health status, lifestyle factors, and quality of life ^1,2^ (**Table S1**). The wording is slightly modified for children and adolescents. In addition, a validated and standardized web-based food frequency questionnaire (FFQ) ^3^ and a set of physical activity questions ^4^ are administered to assess the study participants’ diet and their usual physical activities during the preceding 12 months.

The questionnaire for IBD patients also includes questions about their IBD (**Table S1**), thereby complementing the physician questionnaire that patients are asked to have filled out by their treating physician during the next visit. The physician questionnaire (**Table S1**) inquires detailed disease-related information and includes established and validated questions and scores, such as the Harvey-Bradshaw-Index (HBI), the Mayo Score, and the Crohn’s Disease Activity Index (CDAI). All self-reported IBD diagnoses were validated against the physician questionnaires and additional medical records. Diagnoses were classified into the categories Crohn’s disease (CD) and Ulcerative colitis (UC) and unclassified Inflammatory Bowel Disease, which combines Indeterminate colitis, not defined IBD, suspicion of CD, suspicion of UC, suspicion of IBD, and microscopic colitis (i.e. collagenous colitis, lymphocytic colitis).

***Collection, work-up, and storage of biomaterial***

At the initial (baseline) assessment, blood, stool, and hair samples are collected from all participants (**Table S2**), including approximately 35 ml blood from adult participants and adolescents (12 to 18 years) and 15 ml from children between 7 and 11 years of age. Study

participants also receive sets for the self-collection of stool and hair, accompanied by written instructions on how to collect the sample. Upon receipt at the study center, the biomaterial samples are pseudonymized and stored in the local popgen biobank at Kiel University (**Table S2**).

## Data management, and privacy protection

All clinical data are pseudonymized and stored separately from the identifying data in a central study database at the Institute of Epidemiology and the popgen biobank at Kiel University. The data management and the privacy protection concept of popgen has been reviewed and approved by the independent data protection authorities of Schleswig-Holstein (ULD) and the ethics committee of the Medical Faculty of Kiel University.

In this work, we focused on keeping our study data in accordance with the FAIR principles ^5^. That means that data must be findable, accessible, interoperable and reusable. Also, as we used data that contains a fingerprint of the donor, data protection is of utmost importance. For this purpose, we established andata management tool called iRods (Rule-Oriented Data management systems) for internal use {iRODS Consortium. https://irods.org}. Each user has to log in to the system with an account via API and has access only to content intended for them. Data is shared between the users via subproject groups to only individuals who are in the authorized group. Users outside this group have no access to the data stored there. This guarantees secure use of sensitive data. In addition, the rights system allows a distinction to be made as to whether a user only has viewing rights or can modify the data. The system allows metadata to be stored for the project as json-files. This not only allows the metadata to be closely located to the research data, but also to be searchable directly within the system. Likewise, it also allows the data to be reused at a later date. The metadata input mask was

created for this purpose in compliance with the STORMS checklist intended for microbiome studies ^6^.

## Supplemental Results:


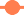
 CD
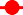
 UC
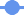
 Control

10 6


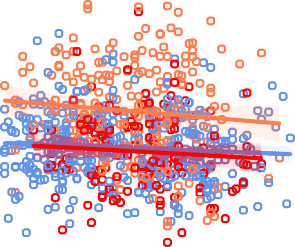
5


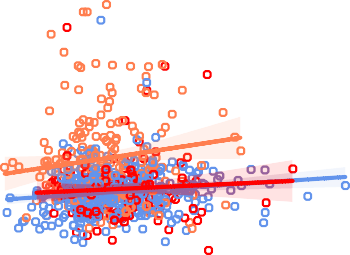


8

ASCA IgA (log)

ASCA IgG (log)

4

6 3

4 2

1

2

0

0 −1

−2 0 2 4

BMI (scaled)

−3 −2 −1 0 1 2 3

Age (scaled)


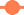
 CD
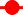
 UC
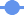
 Control Male Female

6 6


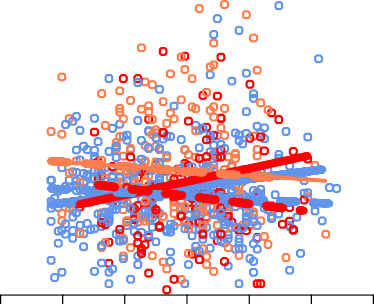


5


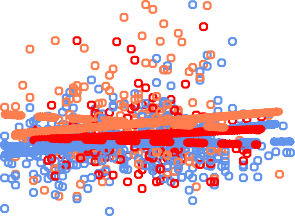


4

GP2 IgA (log)

GP2 IgG (log)

4

2 3

2

0

1

−2

−3 −2 −1 0 1 2 3

Age (scaled)

0

−3 −2 −1 0 1 2 3

Age (scaled)

1.0

0.8

Calprotectin (relative)

0.6

0.4

0.2


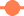
 CD
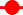
 UC
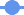
 Control


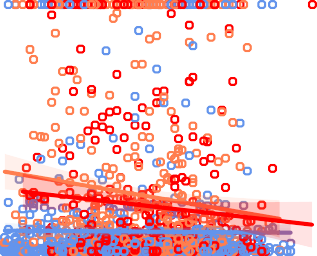
5

4

CRP (4 X)


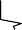
 3


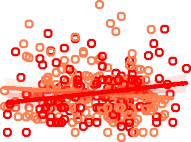
2

1

0.0

−3 −2 −1 0 1 2 3

Age (scaled)

0

−2 0 2 4

Age (scaled)


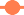
 CD
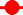
 UC
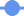
 Control Male Female


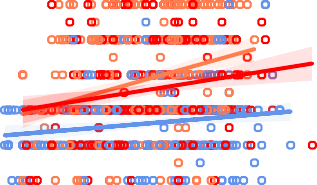
400 8

300 6


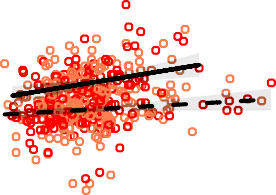


Hb (X²)

Bristol stool score

200 4

100 2

0

−2 0 2 4

BMI (scaled)

0

−3 −2 −1 0 1 2 3

Age (scaled)

**Figure S1:** Scatterplots show the association of immune/physiological parameters (anti ASCA-IgA/IgG, anti GP2-IgA/IgG, relative calprotectin levels, CRP, Hb, Bristol stool score) with the main anthropometric variables (IBD condition (excluding uIBD), gender, BMI, age) . Plots display the modelling results after model selection minimizing AIC. Model statistics are in Table 2 & Table S5. The polygons highlight the 95% CI.

**A** 8

Contr. CD UC uIBD


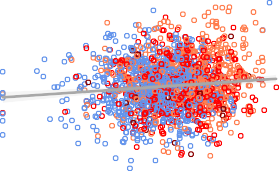

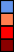


*t* = 4.3092 *P(t)* = 1.7385×10-5

*adj. R* 2 = 0.0113

ρ = 0.0834 *P* = 0.0007

8 *t* = 1.388 *P(t)* = 0.1653

8 *t* = 3.5412 *P(t)* = 0.0004


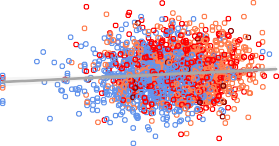


*adj. R* 2 = 0.0125

ρ = 0.0694 *P* = 0.0046

6 6 6

**Polygenic Risk Score (CD)**

**Polygenic Risk Score (UC)**

**Polygenic Risk Score (IBD)**

4 4 4

2 2 2

0 0 0

−2 −2 −2

0.0 0.2 0.4 0.6 0.8 1.0 1.2 0.0 0.2 0.4 0.6 0.8 1.0 1.2 0.0 0.2 0.4 0.6 0.8 1.0 1.2


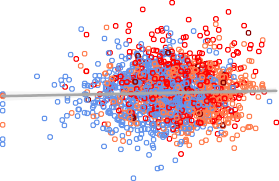


*adj. R* 2 = 0.0013

ρ = 0.0107 *P* = 0.6637

**MD−Index MD−Index MD−Index**

**B** 0.6 0.6 0.6


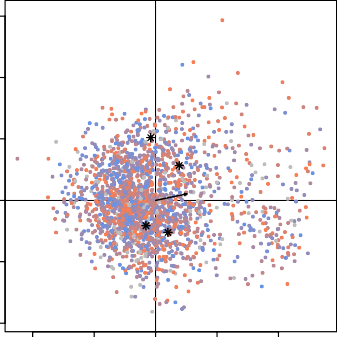

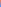


**PRS−CD PRS-CD**

5.486 *F*1,1666=1.3254, *P*=0.0002, *adj.R*2=0.0002

4.468 **PRS-CD + covariates**

3.449 *F*1,1592=1.3381, *P*=0.0003, *adj.R*2=0.0002

2.430

1.411

0.393

−0.626

NA

**PRS−CD**


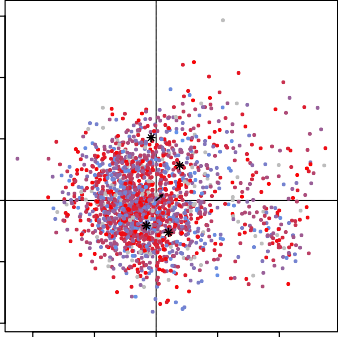

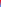


**PRS−UC PRS-UC**

6.428 *F*1,1666=1.1314, *P*=0.0257, *adj.R*2<0.0001

5.477 **PRS-UC + covariates**

4.526 *F*1,1592=1.1584, *P*=0.0120, *adj.R*2<0.0001

3.575

2.623

1.672

0.721

NA

**PRS−UC**


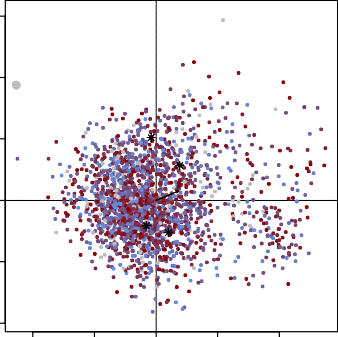

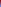


**PRS−IBD PRS-IBD**

4.627 *F*1,1666=1.3297, *P*=0.0005, *adj.R*2=0.0002

3.863 **PRS-IBD + covariates**

3.100 *F*1,1592=1.3257, *P*=0.0005, *adj.R*2=0.0002

2.336

1.572

0.808

0.044

NA

**PRS−IBD**

0.4

0.4

0.4

0.2

**NMDS2**

0.2

0.2

0.0

**NMDS2**

**NMDS2**

0.0

0.0

−0.2

−0.2

−0.2

−0.4

−0.4

−0.4

−0.4 −0.2 0.0 0.2 0.4

**NMDS1**

−0.4 −0.2 0.0 0.2 0.4

**NMDS1**

−0.4 −0.2 0.0 0.2 0.4

**NMDS1**

**Figure S2: (A)** Correlation of PRS with the microbial dysbiosis index (MD-index^7^) show signifcant positive relationships between microbial dysbiosis and genetic predisposition for CD and IBD, as based on *LDpred2* prediction ^8^. **(B)** Genetic predisposition for either CD UC or general IBD show significant correlation to taxonomic community distance as based on PERMANOVA on Bray-Curtis distances derived from ASV abundances at the baseline time point.

**PRS-CD**

P FDR=0.0020

P FDR=0.0019

8

6

4

2

0

−2

PFDR=0.0029

8

27

6

4

2

0

−2

P FDR=0.0028 P FDR=0.0712 P FDR=0.0028

8

P FDR=0.0019 P FDR=0.0019

6

4

2

0

−2

PFDR=0.01

27

PFDR=0.01

8

**PRS−UC**

**PRS−IBD**

P FDR=0.0170

8

P FDR=0.0110

P FDR=0.0080 P FDR=0.0210 P FDR=0.0210

P FDR=0.00003 P FDR=0.02000 P FDR=0.00004

6 6

4 4

**CD**

2 2

0 0

−2 −2

8 8

6 6

4 4

**UC**

2 2

0 0

−2 −2

8 8

6 6

4 4

**IBD**

2 2

0 0

−2 −2

**IBD−affected**

**Contr. FDR**

**Contr. >FDR**

**Contr. unrel.**

**Contr. (random)**

**IBD−affected**

**Contr. FDR**

**Contr. >FDR**

**Contr. unrel.**

**Contr. (random)**

**IBD−affected**

**Contr. FDR**

**Contr. >FDR**

**Contr. unrel.**

**Contr. (random)**

**Figure S3: (A)** Differences of *LDpred2* 7 based polygenic risk scores for CD, UC or general IBD affected individuals (CD/UC/IBD) and their healthy first degree relatives, distant relatives, unrelated controls, or all available healthy controls. All pairwise comparisons were made via Wilcoxon rank test and corrected for multiple testing via FDR. Affected individuals have on average a higher PRS than even their healthy family members, hinting towards a larger accumulation of risk variants in these individuals even in comparison to closely related family members. Further more, CD-PRS and UC-PRS are specific to the respective patient population, while the combined IBD-PRS is more general.

10 *t* = 1.7572 *P(t)* = 0.0793

8

**PRS-IBD**

ASCA−IgA (log)

6

4

2

0


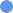
 Control

6 *t* = 2.0435 *P(t)* = 0.0414


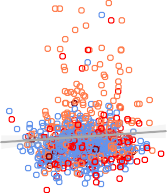


*adj. R* ^2^ = 0.0058

ρ = 0.0706 *P* = 0.0505


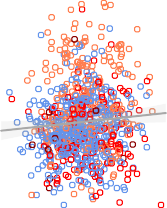


*adj. R*^2^ = 0.0077

ρ = 0.0685 *P* = 0.058

5

ASCA−IgG (log)

4

3

2

1

0


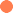
 CD
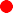
 UC

1.0


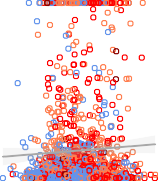


Calprotectin (relative concentration)

0.8

0.6

0.4

0.2

0.0


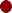
 uIBD

*t* = 1.5262 *P(t)* = 0.1272

*adj. R* ^2^ = 0.005

ρ = 0.02 *P* = 0.4206

8 *t* = 0.2736 *P(t)* = 0.7845

6


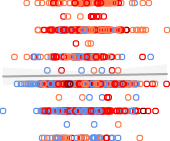


*adj. R* ^2^ = 0.0148

ρ = 0.0156 *P* = 0.6661

Bristol stool score

4

2

0

0 1 2 3 4 5 6

Polygenic Risk Score (IBD)

0 1 2 3 4 5 6

Polygenic Risk Score (IBD)

0 1 2 3 4 5 6

Polygenic Risk Score (IBD)

0 1 2 3 4 5 6

Polygenic Risk Score (IBD)

10

8

**PRS-UC**

ASCA−IgA (log)

6

4

2

0

*t* = −1.9241 *P(t)* = 0.0547

0 2 4 6 8


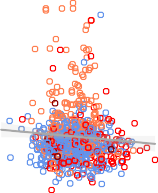


*adj. R* ^2^ = 0.0066

ρ = −0.0432 *P* = 0.2316

ASCA−IgG (log)

Polygenic Risk Score (UC)

6

5

4

3

2

1

0

*t* = −1.9613 *P(t)* = 0.0502

0 2 4 6 8


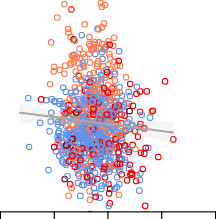


*adj. R*^2^ = 0.0073

ρ = −0.0387 *P* = 0.2847

Polygenic Risk Score (UC)

1.0

0.8

Calprotectin (relative concentration)

0.6

0.4

0.2

0.0

*t* = 1.2749 *P(t)* = 0.2025

*adj. R* ^2^ = 0.0045

ρ = −2e−04 *P* = 0.9921


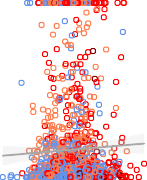


Bristol stool score

0 2 4 6 8

Polygenic Risk Score (UC)

8

6

4

2

0

*t* = 0.6233 *P(t)* = 0.5332


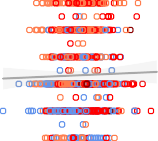


ρ = 0.0413 *P* = 0.2537

0 2 4 6 8

*adj. R*^2^ = 0.0152

Polygenic Risk Score (UC)

**Figure S4:** Scatterplots show the association of immune / physiological parameters (anti ASCA-IgA/IgG, relative calprotectin levels, Bristol stool score) with the *LDpred2* ^7^ based polygenic risk scores for UC and IBD using LMs correcting for relevant covariates (age, BMI, gender), as well as Spearman rank correlation results. Grey polygons highlight the 95% CI.

***Acinetobacter Halomonas Raoultella Proteus Klebsiella***

***Escherichia/Shigella***

***Enterobacter Enterobacteriaceae uncl.***

***Shewanella***

***Desulfovibrio Sutterella Parasutterella Comamonas Fusobacterium Firmicutes uncl.***

***Veillonella***

***Propionispira***

***Dialister Phascolarctobacterium Acidaminococcus Clostridium XVIII Catenibacterium Clostridiales uncl.***

***Subdoligranulum Ruminococcus Oscillibacter Intestinimonas Faecalibacterium Clostridium IV Ruminococcaceae uncl.***

***Roseburia Clostridium XlVb Clostridium XlVa Butyrivibrio***

***Blautia***

***Anaerostipes Lachnospiraceae uncl. Clostridium sensu stricto***

***Streptococcus Lactococcus Lactobacillus Granulicatella Bacteroidales uncl.***

***Alistipes Prevotella Paraprevotella Prevotellaceae uncl. Parabacteroides Odoribacter Coprobacter Butyricimonas Barnesiella Bacteroides Senegalimassilia***

***Collinsella Propionibacterium Bacteria uncl.***

−30 −20 −10 0 10 20 30

−40 −20 0 20 40

30 20 10

0 −10

−20

−30

−30 −20 −10 0 10 20 30

**CD Control**

**UC Control**

**Diseased / IBD**

**Control**

**UC CD**

**
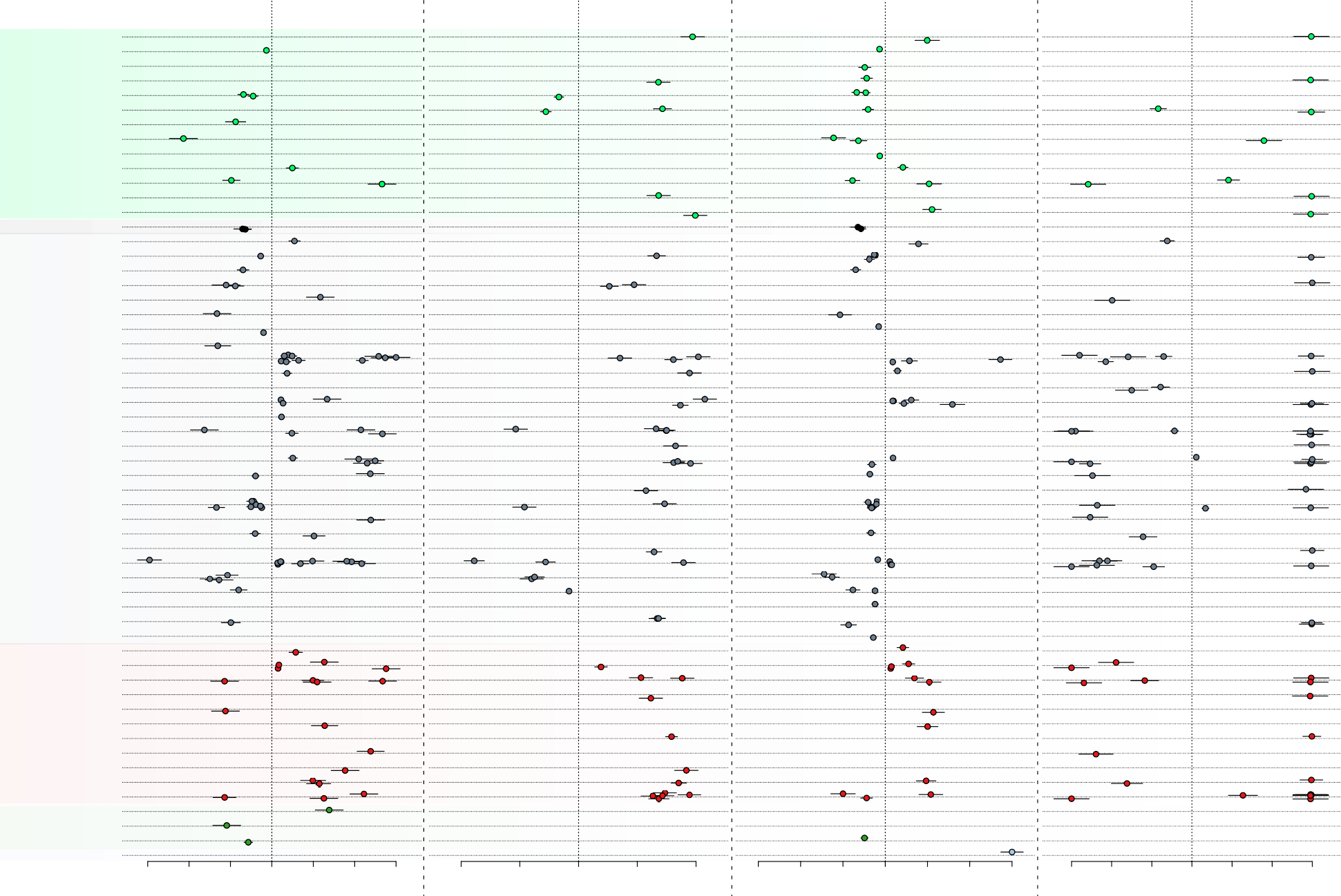
log_2_ Fold Change**


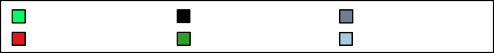


***Proteobacteria Bacteroidetes***

***Fusobacteria Actinobacteria***

***Firmicutes Bacteria uncl.***

**Figure S5:** Differential abundance analyses of ASVs ^8^ based on the first follow-up time point (F1, Table S11). Displayed are the log fold changes for each ASV clustered by genus classification, including standard errors of the fold changes as indicated by individual doted lines. Color bars indicate the phylum membership. DA only displays signifcant differential abundance for the respective comparison/contrast (P_FDR_ ≤ 0.05).

***Akkermansia Proteobacteria uncl.***

***Haemophilus Raoultella Proteus Pluralibacter Morganella Klebsiella***

***Escherichia/Shigella***

***Enterobacteriaceae uncl.***

***Desulfovibrio Burkholderiales uncl.***

***Sutterella Parasutterella Fusobacterium Firmicutes uncl.***

***Veillonella Propionispira Megasphaera***

***Megamonas Dialister***

***Acidaminococcus Turicibacter***

***Clostridium XVIII Catenibacterium***

***Erysipelotrichaceae uncl. Clostridiales uncl. Subdoligranulum***

***Ruminococcus Oscillibacter Intestinimonas Faecalibacterium Butyricicoccus Ruminococcaceae uncl.***

***Romboutsia Intestinibacter Ruminococcus2***

***Roseburia Coprococcus Clostridium XlVb Clostridium XlVa Cellulosilyticum***

***Blautia Anaerostipes***

***Lachnospiraceae uncl. Clostridium sensu stricto***

***Streptococcus Enterococcus Streptophyta uncl. Bacteroidales uncl.***

***Alistipes Prevotella Parabacteroides Odoribacter Coprobacter Butyricimonas Barnesiella Bacteroides Senegalimassilia Propionibacterium Bacteria uncl.***

−30 −20 −10 0 10 20 30

−40 −20 0 20 40

30 20 10

−10

−20

−30

−30 −20 −10 0 10 20 30

**CD Control**

**UC Control**

**Diseased / IBD**

**Control**

**UC CD**

**log2 Fold Change**


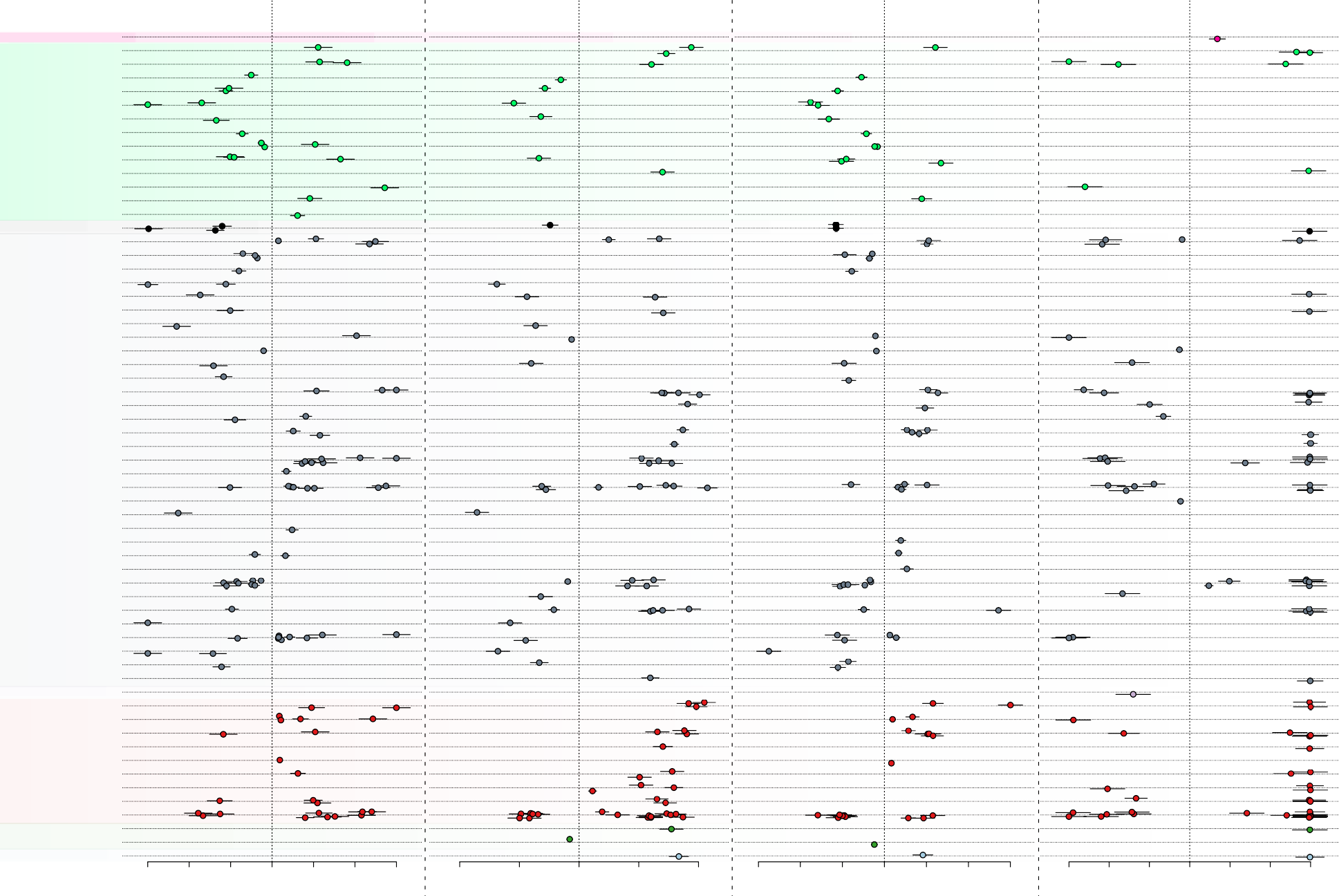

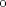

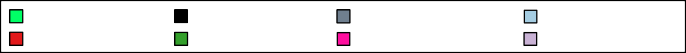


***Proteobacteria Bacteroidetes***

***Fusobacteria Firmicutes Actinobacteria Verrucomicrobia***

***Bacteria uncl. Cyanobacteria***

**Figure S6:** Differential abundance analyses of ASVs ^8^ based on the second follow-up time point (F2, Table S12). Displayed are the log fold changes for each ASV clustered by genus classification, including standard errors of the fold changes as indicated by individual doted lines. Color bars indicate the phylum membership. DA only displays signifcant differential abundance for the respective comparison/contrast (P_FDR_ ≤ 0.05).


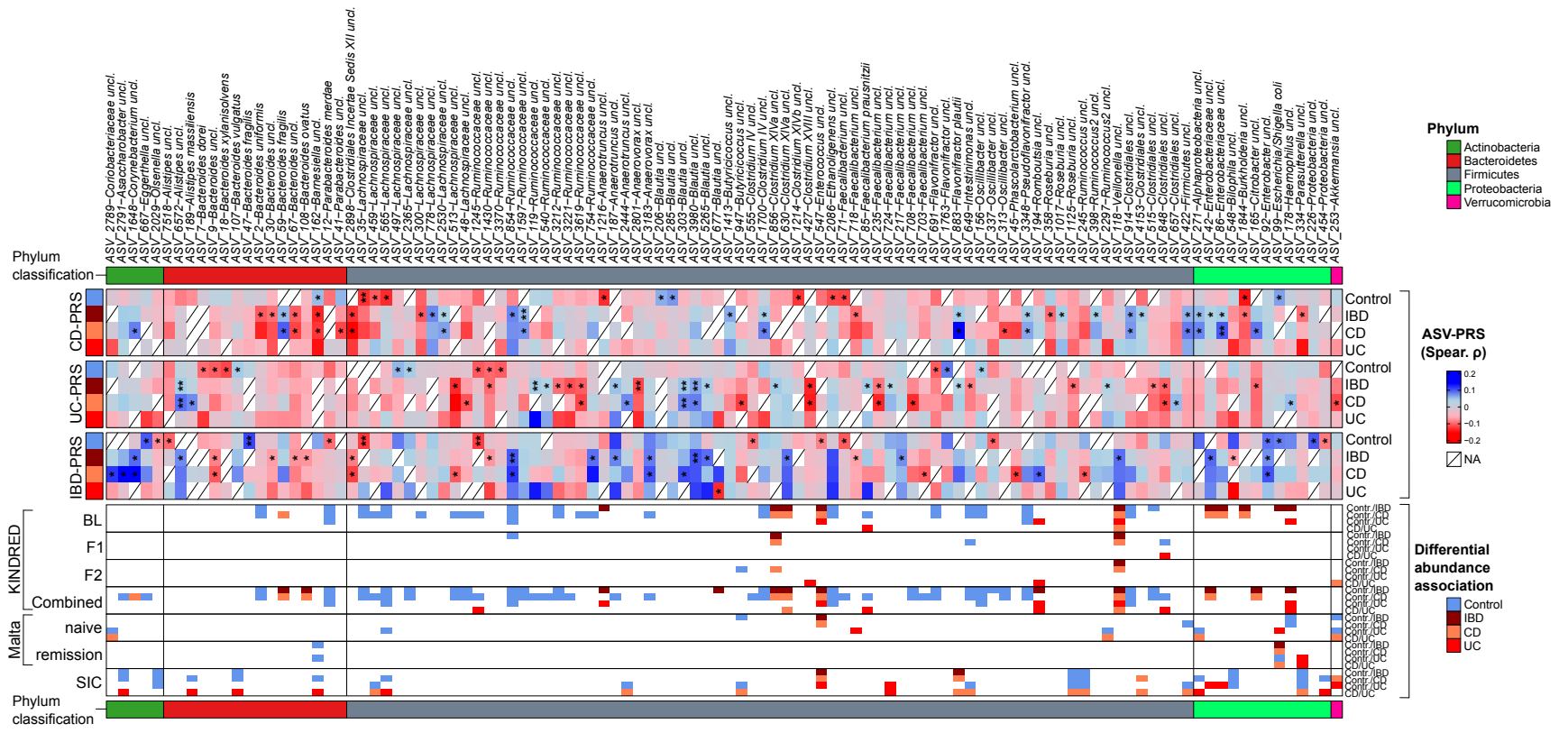


**Figure S7:** Partial correlation of CLR transformed taxon abundances with *LDpred2* 7 derived polygenic risk scores for CD, UC, and general IBD (CD, UC, uIBD) via ppcor^9^, combining the *P* values of Spearman-, Kendall-, and Pearson correlations via Brown’s method and corrected via FDR ^10^. Correlations were adjusted for age, gender, and BMI. Spearman *ρ* is used to visualize correlation strength between taxa and clinical measures (# *P_FDR_*≤0.1000, * *P_FDR_*≤0.0500, ** *P_FDR_*≤0.0100, *** *P_FDR_*≤0.0010). Overlapping, significant patterns of differential abundance for the respective taxa in the KINDRED cohort, Maltese- and Swedish SIC cohort are indicated in the bottom color bars (see Table S14).

# Average differences Correlation with MD-index

1000

800


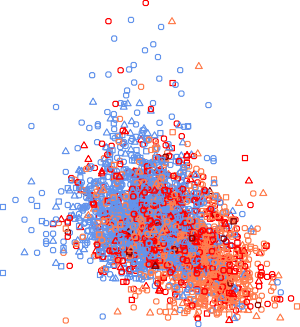

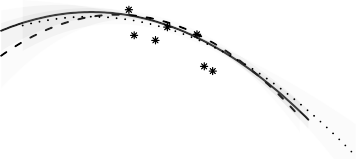


Control CD

UC uIBD

BL F1 F2

furture Onset

BL-poly: F2,1809=226.9725, P=1.1235x10-88, adj.R2=0.1997

F1-poly: F2,645=83.7775, P=4.5236x10 ^-33^, adj.R^2^=0.2037

F2-poly: F2,536=55.7446, P=1.0136x10 -22, adj.R2=0.1691

*P*=0.0267 *P*=5.678×10^15^ *P*<2.20×10^16^

*P*=0.2187

800

**Chao1 species richness**

600

**Chao1 species richness**

600

Chao1

400

400

200

200

0

250

**Control Onset CD UC uIBD**

*P*=0.0905

*P*<2.20×10^16^

0

0.0 0.2 0.4 0.6 0.8 1.0 1.2 1.4

MD−Index

200


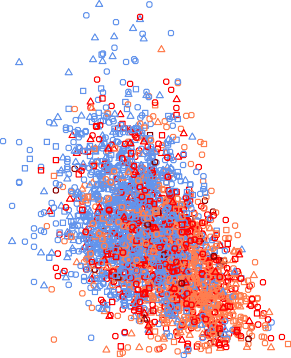

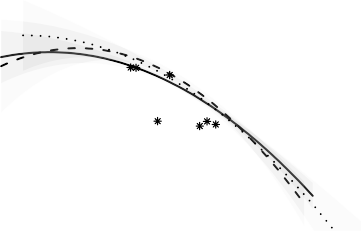


200

BL-poly: F2,1809=243.4262, P=2.3953x10 -94, adj.R2=0.2112

F1-poly: F2,645=101.7966, P=3.7791x10 ^-39^, adj.R ^2^=0.2376

F2-poly: F2,536=89.4681, P=2.9686x10 -34, adj.R2=0.2475

150

Control CD

UC uIBD

BL

F1

F2

furture Onset

100

50

0

*P*<2.20×10^16^

*P*=0.1984

**Shannon H (effective)**

150

**Shannon H (effective)**

Shannon (effective)

100

50

0

**Control Onset CD UC uIBD**

0.0 0.2 0.4 0.6 0.8 1.0 1.2 1.4

MD−Index

**Figure S8:** Alpha diversity analyses focussing on differences of onset cases, CD patients, UC patients, and uIBD patients to healthy control individuals, based on species richness (Chao1) and general complexity (Shannon H). Differences were assessed via pairwise Wilcoxon tests. Correlation of the MD dysbiosis index ^11^ with the different alpha diversity measures in the three available time points (Chao1: BL (poly): F_2,1809_=226.97, P<2.2×10^-16^, adj.R^2^=0.1997; F1 (poly): F_2,645_=83.778, P<2.2×10^-16^, adj.R^2^=0.2037; F2 (poly): F_2,536_=55.745, P<2.2×10^-16^, adj.R^2^=0.1691; Shannon:BL (poly): F_2,1809_=243.43, P<2.2×10^-16^, adj.R^2^=0.2112; F1 (poly): F_2,645_=101.80, P<2.2×10^-16^, adj.R^2^=0.2376; F2 (poly): F_2,536_=89.468, P<2.2×10^-16^,

adj.R^2^=0.2475; linear models) ^12,13^. Grey polygons highlight the 95% CI and “*****“ highlight the

still healthy, future onset patients. Poly indicates a second order polynomial fit, instead of a linear model fit. See Table S8.

**Baseline Follow-up 1 Follow-up 2**


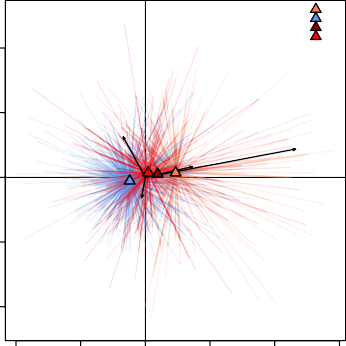


**Stress:** 0.285

**B**

CD

Contr. uIBD UC

**BMI**

**QOL-index**

**Calprotectin**

**Hb/Hp−0**

+ +**Hb/Hp−1**

**Age**


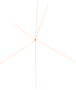

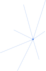

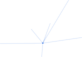

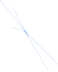

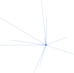

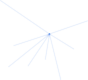

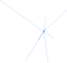

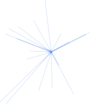

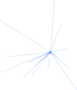

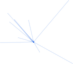

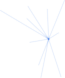

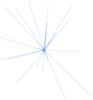

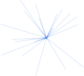

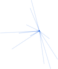

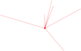


**Stress:** 0.283

**C**

CD

Contr. uIBD UC

**Bristol stool score**

**QOL index FSS index**

**CRP**

**Age**

0.6


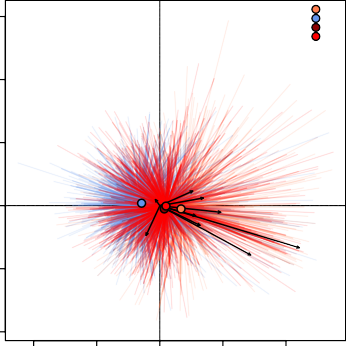


**Stress:** 0.290

**A**

CD

Contr. uIBD UC

**Hb/Hp−0**++

**Age**

**Hb−0**

**GP2 IgA**

**GP2 IgG**

**IBD assessment**

**Hb−1 Calprotectin Hb/Hp−1**

**ASCA IgA**

**BMI**

**Bristol stool score**

**ASCA IgG**

0.4

0.4

0.4

0.2

0.2

NMDS 2

NMDS 2

0.2

**IBD**

NMDS 2

0.0

0.0

0.0

−0.2 −0.2

−0.2

−0.4 −0.4

−0.4

−0.4 −0.2 0.0 0.2 0.4

NMDS 1

−0.4 −0.2 0.0 0.2 0.4 0.6

NMDS 1


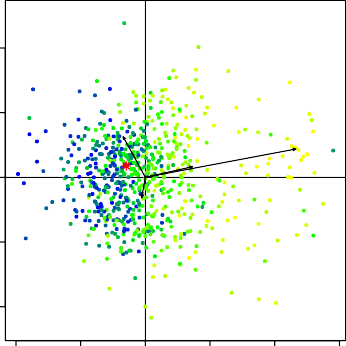

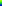


**Stress:** 0.285

**future IBD onset**

**E**

**MD Index**

1.037

0.864

0.691

0.518

0.346

0.173

0.000

**BMI**

**QOL-index**

**Calprotectin**

**Hb/Hp−0**+ +**Hb/Hp−1 Age**

−0.4 −0.2 0.0 0.2 0.4

NMDS 1


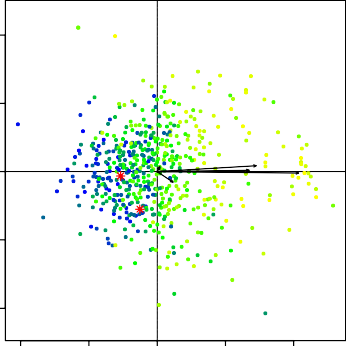

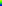


**Stress:** 0.283

**F**

**MD Index**

1.225

1.021

0.817

0.612

0.408

0.204

0.000

**Bristol stool score**

**QOL index FSS index**

**CRP**

**Age**

0.6


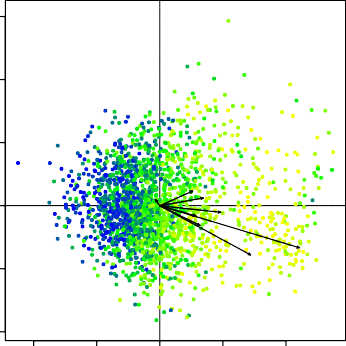

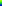


**Stress:** 0.290

**future IBD onset**

**D**

**MD Index**

1.070

0.891

0.713

0.535

0.357

0.178

0.000

**Age Hb−0**

**GP2 IgA**

**GP2 IgG**

**Hb/Hp−0 Hb/Hp−1**

+++

**Hb−1 Calprotectin**

**ASCA IgA**

**IBD assessment**

**BMI**

**Bristol stool score**

**ASCA IgG**

0.4

0.4

0.4

**Physiological measures**

NMDS2

0.2

0.2

0.2

0.0

0.0

0.0

NMDS2

NMDS2

−0.2 −0.2

−0.2

−0.4 −0.4

−0.4

−0.4 −0.2 0.0 0.2 0.4

NMDS1

−0.4 −0.2 0.0 0.2 0.4 0.6

NMDS1


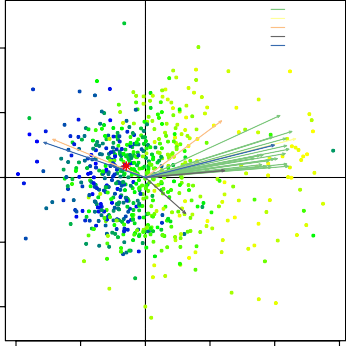


**Stress:** 0.285

**H**

AA FA

carbon vitamin

makro nutrients

**KPG**

**ECYS**

**KPOR ZA**

**EGLY ENA EALA**

**ZE F204**

**EARG**

**ETRP EPHE EASP**

**ESER EHIS**

**ELEETUYR ELYS**

**EVALETHR EEA**

**VB12**

**EMET**

**EILE**

**VAR**

−0.4 −0.2 0.0 0.2 0.4

NMDS1


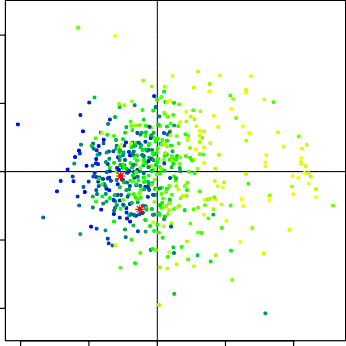


**Stress:** 0.283

**I**

0.6


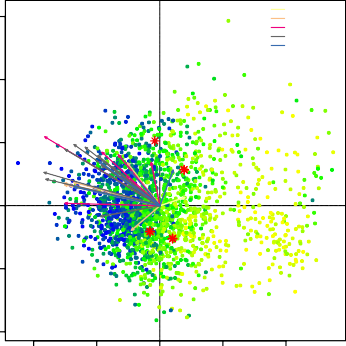


**Stress:** 0.290

**G**

FA

carbon mineral vitamin

makro nutrients

**MK**

**VBV9 B9F VB9G**

**VC MJ**

**MCU**

**ZA**

**VB3 VAC**

**VB5 VB7**

**KBN**

**KBL MF**

**ZM ZW**

**MMG**

**KBU**

**KBC ZB F204**

**MP**

**VB2**

**KDL**

0.4

0.4

0.4

0.2

**Nutrient intake**

NMDS2

0.2

0.2

NMDS2

NMDS2

0.0

0.0

0.0

−0.2 −0.2

−0.2

−0.4 −0.4

−0.4

−0.4 −0.2 0.0 0.2 0.4

NMDS1


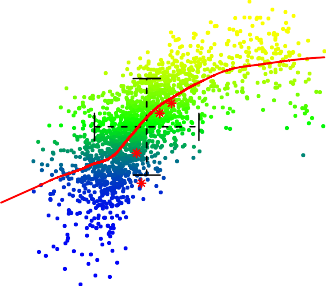


**J**

*ρ*=0.7614, *P*<2.20 × 10^-16^

−0.4 −0.2 0.0 0.2 0.4 0.6

NMDS1

−0.4 −0.2 0.0 0.2 0.4

NMDS1

1.0

1.0 **K**

1.2

**L**


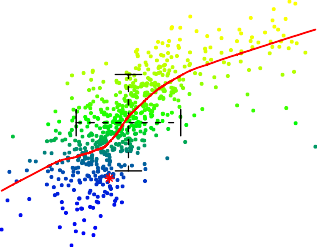

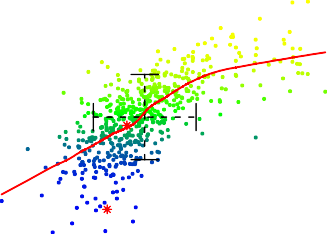


*ρ*=0.7244, *P*<2.20 × 10^-16^

0.8

**Dysbiosis gradient**

**MD−Score**

0.8

1.0

0.8

**MD−Score**

**MD−Score**

0.6

0.6

0.6

0.4

0.4

0.4

0.2

0.2

*ρ*=0.7397, *P*<2.20 × 10^-16^

0.2

0.0

0.0

0.0

−0.4 −0.2 0.0 0.2 0.4

**NMDS1**

−0.4 −0.2 0.0 0.2 0.4 0.6

**NMDS1**

−0.4 −0.2 0.0 0.2 0.4

**NMDS1**

**Figure S9: (A)** Non-metric Multidimensional Scaling (NMDS) of Bray-Curtis distances among baseline samples, **(B)** follow-up 1, and **(C)** follow-up 2, displaying the significant clustering by health conditions and significant correlations of clinical inflammation measures

with community distance (see Table S16, Table S17, Table S23). **(D)** NMDS displaying the gradient of community dysbiosis as expressed by MD-index ^11^, across the three time points, in parallel with significantly correlated clinical measures of inflammation and healthy onset cases highlighted in red (*, develop IBD until the next follow-up). **(G, H, I)** Nutrition (approximated normalized nutrient uptake) was also significantly correlated with community distances. Arrow colors represent different nutrient groups (AA-aminoacids, FA-fatty acids, carbon-carbohydrates, minerals-trace elements, vitamins, makro nutrients-larger nutrient clusters (i.e. proteins, fats, water)). **(J, K, L)** Correlation of MD-index and the first NMDS axis showing a clear gradient of dysbiosis in the community (Spearman rank correlation). Onset cases are distributed in the range of standard deviation around the mean of the community distribution (NMDS1) and the severity of dysbiosis (MD-index).

**A**

Bristol stool score

Physiological parameters

*******

**#**

*****

**B** Pathologies

IBD (CD/UC/IBD/Contr.)


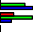

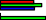


IBD (healthy/diseased) stoma

*******

*******

*******

obstipation (3 m.) *******

CRP

diarrhea (3 m.)

******

*******

*******

Quality of life index

ASCA IgG


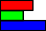
IBD assessment


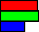
Age


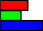
Calprotectin

Fatigue Severity Scale index


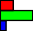
BMI

Stool Hb/Hp
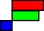
 GP2 IgG
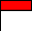
 GP2 IgA
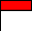
 ASCA IgA
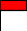


Stool Hb

Baseline Follow-up 1

Follow-up 2

*** #**

*******

*******

*****

*******

*****

*******

******

*******

*****

*******

******

******

*****

*******

*******

*******

*******

stenoses fistulae

diarrhea (3 - 6 m.)

abdominal pain (6 m.) gall stones

anemia

joint pain (6 m.) fatigue (6 m.) back pain (6 m.) arthritis osteoporosis anal fissure kidney disease nausea (6 m.) pancreatitis aphthae (mouth) eye inflammation liver disease induration (6 m.) cancer (last year) skin disease food intoler allerg

weight loss (6 m.) appendicitis psoriasis

depression diagnosis other afflictions (6 m.) lactose intolerance food allergy

COPD ever hypertension cancer

PSC

respiratory disease contact allergy

leg thrombosis

parodontitis measles allergy

*****

*****

*****

*****

*******

*******

*****

******

*****

******

*****

*****

*****

*****

*****

*******

*****

*******

*******

******

*******

******

*******

*******

*******

*******

*******

*********

*****

*** ***

*******

******

***#****

*******

*******

*******

*******

*******

*******

Baseline Follow-up 1

Follow-up 2

**C**

antiinflammatory medication antidiarrhetics

0.000 0.001 0.002 0.003 0.004

adjusted R^2^

Pharamceuticals

**D**

eicosatetraenoic acid (F204) water−insoluble fibers (KBN) fibre (ZB)

vit. B9 (Folate) (VB9)

0.000 0.001 0.002 0.003 0.004 0.005

adjusted R^2^

Nutrition

*******


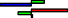

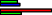


*****

*****

******

antidiarrhetics 12m medication general 5-ASA alternatives Sip tube feeding glucocorticoids biologicals budesonid (12 m.)


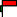

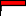

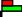

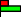

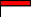

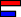


*******

*******

*******

*******

*******

*******

*******

*******

*******

*******

*******

*******

*******

*******

*******

*******

*******

*******

*******

*****

*******

*******

**# *****

*******

*******

*****

*******

*******

*****

*******

******

*******

*******

*******

Baseline Follow-up 1

Follow-up 2

antibiotics (12 m.)

biologicals (12 m.)

mesalazin (12 m.)

cortisone (12 m.) antibiotics antibiotics (6 m.)

sulfasalazin (12 m.) current medication immunosuppressives course steroid azathioprin (12 m.) contraceptives infliximab (12 m.)

methotrexat (12 m.)

medication regularly glucocorticoids

(12 m.)

vit. B9 (free folic acid) (VB9F) alcohol (ZA)

aspartic acid (EASP)

vit. B9 (total folic acid) (VB9G) poly−uronic acid (KBU) cellulose (KBC)

glycine (EGLY) alanine (EALA) cysteine (ECYS) arginine (EARG) histidine (EHIS) lysine (ELYS) fluorine (MF) lignin (KBL)

vit. A (beta−carotene) (VAC) iodide (MJ)

vit. A (retinol) (VAR) methionine (EMET)

vit. B12 (cobalamin) (VB12) protein (ZE)

vit. B5 (pantothenic acid) (VB5) vitamin C (VC)

threonine (ETHR) essential amino acid (EEA) potassium (MK)

non−essential amino acid (ENA) oligosacc. adsorbable (KPOR) vit. B3 (Niacin) (VB3)

vit. B7 (biotin) (VB7) isoleucine (EILE) valine (EVAL) tyrosine (ETYR) phosphorus (MP)

phenylalanine (EPHE) leucine (ELEU) glycogen (KPG) tryptophan (ETRP) minerals (ZM)

serine (ESER)

vit. B2 (riboflavin) (VB2) magnesium (MMG)

consumed food quantity (gram) water (ZW)

******

*****

*****

******

******

******

*****

*****

*****

*****

*****

*****

*****

*****

*****

**** #**

*****

*****

*****

**** ***

******

*****

*****

******

*****

*****

*****

******

*****

*****

*****

******

*****

*****

*****

*****

*****

*****

*****

*****

******

******

immunosuppress (12 m.)

copper (MCU) lactose (KDL)

****** Baseline

***** Follow-up 1

Follow-up 2

0.000 0.001 0.002 0.003 0.004

adjusted R^2^

0.0000 0.0002 0.0004 0.0006 0.0008 0.0010

adjusted R^2^

**Figure S10:** Visualization of the explained variation of significant anthropometric variables as based on serial PERMANOVA of Bray-Curtis distances in all three time points available and focused on **(A)** physiological measures (Table S17), **(B)** different reported pathologies (Table S21), **(C)** use of pharmaceuticals (Table S22), and **(D)** nutrient intake as derived from FFQ data (Table S23). Variables are displayed if they show significant clustering in at least one time point (# *P_FDR_*≤0.1000, * *P_FDR_*≤0.0500, ** *P_FDR_*≤0.0100, *** *P_FDR_*≤0.0010).

Baseline Follow-up 1 Follow-up 2

0.80

0.75

Bray-Curtis (ASV)

**Distance to centroid**

***F*** [ 3 , 1808 ] **=** 46.0315 , ***P=*** 1e−04

*P*=0.1798 *P*=0.0001 *P* =0.0001

0.80

0.75

***F*** [ 3 , 644 ] **=** 26.8879 , ***P=*** 1e−04

*P*=0.8186 *P*=0.0001 *P* =0.0001

0.80

0.75

***F*** [ 3 , 535 ] **=** 22.9253 , ***P=*** 1e−04

*P*=0.9206 *P*=0.0001

*P* =0.0001

0.70

0.70

0.70

0.65

0.65

0.65

0.60

0.60

0.60

0.55

0.50

*P* =0.1393 *P*=0.0678

*P* =0.0678

**Control CD UC uIBD**

0.55

0.50

*P* =0.0631 *P*=0.8527

*P* =0.0439

**Control CD UC uIBD**

0.55

0.50

*P* =0.0782 *P*=0.9424

*P* =0.0625

**Control CD UC uIBD**

**Figure S11:** Violinplots visualize differences in community variability between the main health conditions (Table S18), as expressed by the within group distance to the centroid based on Bray-Curtis distances (via *betadisper* function) ^14^. P-values were derived via permutation test of multivariate homogeneity of group dispersions (10000 permutations). Overall, we can see a significant increase of community variability in CD and UC patients compared to healthy controls.

3.0

2.5

CD UC Control

3.0

2.5

2.0

GP2 IgA (log)

1.5

1.0

0.5

0.0

−0.5

0.0 0.2 0.4 0.6 0.8 1.0 1.2

MD Index

2.0

1.5

GP2 IgG (log)

1.0

0.5

0.0

−0.5

0.0 0.2 0.4 0.6 0.8 1.0 1.2

MD Index

4

3

CRP ( X)

2

4

1

0

0.0 0.2 0.4 0.6 0.8 1.0 1.2

MD Index

5

4

IBD assessment score

3

2

1

0

0.0 0.2 0.4 0.6 0.8 1.0 1.2

MD Index

350

300

250

200

Hb (X^2^)

150

100

50

0

0.0 0.2 0.4 0.6 0.8 1.0 1.2

MD Index

60

50

40

30

BMI

20

10

0

0.0 0.2 0.4 0.6 0.8 1.0 1.2

MD Index

**Figure S12:** Association of immune / physiological parameters with the microbial dysbiosis index ^11^ using LMs including relevant covariates (age, BMI, gender, IBD pathology (including uIBD)) after model selection (see Table S20). Polygons highlight the 95% CI.

**A** 8 **B** 1.0

*****

*****

*P*=6.8818×10^-5^ *P*=0.6524 *P*=0.0125 *P*=0.0004 *P*=0.8359 *P*=0.0032

*P*=0.6272 *P*=0.4627 *P*=0.6236

Cluster-1 Cluster-2 Cluster-3

**UC uIBD Contr.**

|  |  |
| --- | --- |
|  |  |
| ***** |  |
|  |  |
|  |  |
|  |  |

6 0.8

**Polygenic Risk Score**

**Cluster composition**

4 0.6

2 0.4

**CD**

0 0.2

-2

**CD-Risk UC-Risk IBD-Risk**

0.0

**Cluster−1 Cluster−2 Cluster−3**

**Clustering of combined time points**

**D E**

Cluster−1 Cluster−2

future IBD onset

Baseline Follow-up 1

Follow-up 2

**MD-Index**

1.225

1.021

0.817

0.612

0.408

0.204

0.000

Cluster−1 Cluster−2

0.6 0.6

0.4 0.4

0.2 0.2

NMDS2

NMDS2

0.0 0.0

−0.2 −0.2

−0.4 −0.4

−0.4 −0.2 0.0 0.2 0.4

NMDS1

−0.4 −0.2 0.0 0.2 0.4

NMDS1

**Figure S13: (A)** Differences of polygenic risk scores for CD, UC or general IBD between the three microbiome community clusters at baseline ^15^. We can see a clearly increased PRS in the Proteobacteria dominated community cluster-3 (pairwise Wilcoxon test). **(B)** Barplots visualize the distribution of health conditions/pathologies between community clusters and

the distribution of future IBD onset patients (“*****”). **(C)** Community clusters of the microbial community including all three time points, as based on Dirichilet Multinomial Mixture modelling (DMM) and optimal clustering determined via Laplace goodness of fit optimization (future IBD onset patients *****). **(D)** Overlay of the Microbial Dysbiosis index gradient and DMM community clusters (outlines), including healthy onset patients before disease manifestations (indicated by *****). The MD-index was higher in cluster-2 (*F*_1,2997_=1199.905, *P*<2.20×10^-16^; LM) and displayed a larger ratio of IBD cases *vs* healthy individuals (*P*<2.20×10^-16^, OR=5.1375; Fisher-test).

***Clostridiales uncl. ASV−117 Clostridium XVIII ramosum ASV−193***

0.05

0.04

0.03

0.02

0.01

0.008

0.006

0.004

0.002

*P*FDR≤0.05

**Figure S14:** Network importance/centrality measures derived at the baseline time point and

highlighting differential abundance associations at each node as derived from *DESeq2*

Centralities range from the number of connections (degree), the position on the shortest paths

within the network (betweeness,

average neighborhood degree of any given vertex (k-nearest neighbor degree

boxes highlight significant associations in KIN and external cohorts with IBD pathologies or

IBD onset. Significance of centralities is derivedfrom *Z*-test against a large collection of

Node PageRank index

***Clostridium XVIII ramosum ASV−193 Clostridiales uncl. ASV−117***

*P*FDR≤0.05

Node degree

***Oscillibacter uncl. ASV−138***

0.000Contr/CD

Contr/UC

ConCtDr/I/BUDC

Contr/CD

Contr/UC

CD/UC

Contr/IBD

Contr/CD

Contr/UC

CD/UC

Contr/IBD

Contr/CD

Contr/UC

CD/UC

Contr/IBD

Contr/CD

Contr/UC

CD/UC

Contr/IBD

Contr/CD

Contr/UC

CD/UC

Contr/IBD

Conttrr//CUDC

CD/UC

Contr/IBD

***Alistipes putredinis***

***ASV−4***

***Clostridium XlVa clostridioforme ASV−103 Clostridium XlVa clostridioforme ASV−103***

0.00Contr/CD

Contr/UC

CoCD/UC

ntr/IBD

Contr/CD

Contr/UC

CoCD/UC

ntr/IBD

Conttrr//CUDC

CoCD/UC

ntr/IBD

Conttrr//CUDC

CoCD/UC

ntr/IBD

Contr/CD

CoCnDtr//UC

Contr/IBD

Contr/CD

CoCnDtr//UC

Contr/IBD

Contr/CD

Contr/UC

ConCtDr/I/BUDC

***Alistipes putredinis***

***ASV−4***

***Oscillibacter uncl. ASV−138***

***Clostridiales uncl. ASV−914 Clostridiales uncl. ASV−515***

***Flavonifractor plautii Lachnospiraceae uncl.***

***ASV−93 ASV−55***

***Oscillibacter uncl. ASV−403 Clostridiales uncl. ASV−914***

***Flavonifractor plautii***

***ASV−93***

***Lachnospiraceae uncl.***

***ASV−64***

***Clostridium XlVa uncl. ASV−1057 Blautia uncl. ASV−753***

***Lachnospiraceae uncl.***

***ASV−55***

***Lachnospiraceae uncl. ASV−3004***

***Lachnospiraceae uncl. ASV−3004 Blautia uncl. ASV−753***

***Alistipes shahii Faecalibacterium uncl.***

***ASV−31 ASV−94***

***Lachnospiraceae uncl.***

***ASV−64***

***Clostridium XlVa uncl. ASV−171***

***Roseburia inulinivorans ASV−293 Ruminococcaceae uncl. ASV−854***

***Bacteroides uncl. ASV−1***

***Ruminococcaceae uncl. ASV−106***

***Ruminococcaceae uncl. ASV−106 Ruminococcaceae uncl. ASV−854 Granulicatella uncl. ASV−1903 Clostridiales uncl. ASV−1483 Clostridium XlVa uncl. ASV−266 Romboutsia uncl. ASV−576***

***Fusicatenibacter uncl. ASV−407 Oscillibacter uncl. ASV−1723 Veillonella uncl. ASV−222***

***Veillonella uncl. ASV−222 Oscillibacter uncl. ASV−299 Clostridium XlVa uncl. ASV−1057 Granulicatella uncl. ASV−1903***

***Erysipelotrichaceae uncl. ASV−975***

***Clostridium XlVa uncl. ASV−1035 Oscillibacter uncl. ASV−126 Oscillibacter uncl. ASV−581***

***Ruminococcaceae uncl. ASV−757***

***Alistipes shahii ASV−31***

***Oscillibacter uncl. ASV−126 Ruminococcaceae uncl. ASV−757***

***Clostridiales uncl. ASV−816 Eggerthella lenta ASV−829 Clostridiales uncl. ASV−1483***

***Faecalibacterium uncl. ASV−94***

***Oscillibacter uncl. ASV−1723***

***Blautia uncl. ASV−371 Romboutsia uncl. ASV−591 Clostridium XlVa uncl. ASV−1035 Flavonifractor uncl. ASV−687 Oscillibacter uncl. ASV−581 Oscillibacter uncl. ASV−157***

***Clostridiales uncl. ASV−1936 Roseburia uncl. ASV−373 Erysipelotrichaceae uncl. ASV−975***

***Lachnospiraceae uncl. ASV−116 Oscillibacter uncl. ASV−157 Clostridiales uncl. ASV−515 Eisenbergiella uncl. ASV−519 Blautia hansenii ASV−599 Eggerthella lenta ASV−713***

***Pseudoflavonifractor uncl. ASV−773 Clostridium XlVa uncl. ASV−856 Clostridiales uncl. ASV−1936***

***Parasutterella uncl. ASV−24***

***Rothia mucilaginosa ASV−2932***

***Fusicatenibacter uncl. ASV−362 Intestinibacter uncl. ASV−284 Faecalibacterium uncl. ASV−229 Clostridium XlVa uncl. ASV−534 Faecalibacterium uncl. ASV−270***

16),

***Alistipes uncl. ASV−7518 Faecalibacterium uncl. ASV−229 Romboutsia uncl. ASV−591 Paraeggerthella uncl. ASV−1558 Anaerovorax uncl. ASV−1730***

***Lachnospiraceae uncl. ASV−116***

***Subdoligranulum uncl. ASV−20***

***Oscillibacter uncl. ASV−299***

***Propionispira uncl. ASV−428***

***Faecalibacterium uncl. ASV−71***

***Enterococcus uncl. ASV−547***

***Clostridiales uncl. ASV−816 Roseburia uncl. ASV−361 Faecalibacterium uncl. ASV−566 Ruminococcaceae uncl. ASV−3619***

generalized importance (PageRank index and the

***Flavonifractor uncl. ASV−687 Clostridium XlVa symbiosum ASV−689 Romboutsia uncl. ASV−823 Anaerotruncus uncl. ASV−1216***

***Oscillibacter uncl. ASV−83***

***Anaerovorax uncl. ASV−2627***

***Lachnospiraceae uncl. ASV−273 Ruminococcaceae uncl. ASV−3619***

***Parasutterella uncl. ASV−142***

***Faecalibacterium uncl. ASV−89***

***Clostridium XlVa uncl. ASV−171 Lachnospiraceae uncl. ASV−1773 Pseudoflavonifractor uncl. ASV−773***

***Veillonella uncl. ASV−118 Turicibacter uncl. ASV−236 Lachnospiraceae uncl. ASV−352***

***Subdoligranulum uncl. ASV−20 Bacteroides uncl. ASV−132 thia mucilag nosa ASV−2932***

*P*FDR≤0.05

***Ro*** ***i***

***Coprobacillus uncl. ASV−100 Clostridium XVIII uncl. ASV−111***

Contr/UC

CD/UC

Contr/IBD

Contr/CD

Contr/UC

CD/UC

Contr/IBD

Conttrr//CUDC

CD/UC

Contr/IBD

Conttrr//CUDC

CD/UC

Contr/IBD

Contr/CD

CoCnDtr//UC

Contr/IBD

Contr/CD

CoCnDtr//UC

Contr/IBD

Contr/CD

Contr/UC

ConCtDr/I/BUDC

0Contr/CD

Contr/UC

ConCtDr/I/BUDC

Contr/CD

Contr/UC

CoCD/UC

ntr/IBD

Contr/CD

Contr/UC

CoCD/UC

ntr/IBD

Contr/CD

Contr/UC

CoCD/UC

ntr/IBD

Contr/CD

Contr/UC

CoCD/UC

ntr/IBD

Contr/CD

Contr/UC

CoCD/UC

ntr/IBD

Conttrr//CUDC

CoCD/UC

ntr/IBD

Node KNN degree

35

30

25

20

15

10

5

***Roseburia uncl. ASV−361 Oscillibacter uncl. ASV−403***

***Clostridium sensu stricto uncl. ASV−421***

*P*FDR≤0.05

***Clostridium XVIII ramosum ASV−193***

***Blautia uncl. ASV−1501***

Node betweenness

0.20

0.15

0.10

0.05

0.00Contr/CD

***Alistipes onderdonkii ASV−13***

***Alistipes uncl. ASV−7518***

***Alistipes uncl. ASV−143***

***Faecalibacterium uncl. ASV−229***

***Ruminococcaceae uncl. ASV−540 Lachnospiraceae uncl. ASV−468 Oscillibacter uncl. ASV−414***

***Alistipes uncl. ASV−16 Clostridiales uncl. ASV−117 Clostridium XlVa uncl. ASV−171***

***Lachnospiraceae uncl. ASV−381 Clostridium IV uncl. ASV−263***

***Lachnospiraceae uncl. Faecalibacterium uncl.***

***ASV−64 ASV−94***

***Odoribacter uncl. ASV−196 Clostridiales uncl. ASV−320***

***Enterococcus uncl. ASV−547 Eggerthella lenta ASV−829***

***Oscillibacter uncl. ASV−156 Ruminococcaceae uncl. ASV−3717 Firmicutes uncl. ASV−561***

***Klebsiella uncl. Alistipes shahii***

***Subdoligranulum uncl.***

***ASV−52 ASV−31 ASV−20***

***Coprobacter uncl. ASV−231 Oscillibacter uncl. ASV−221***

***Enterobacteriaceae uncl. ASV−184 Fusobacterium nucleatum ASV−213***

***Bacteroides uncl. ASV−29 Anaerotruncus uncl. ASV−2444 Clostridium XlVa uncl. ASV−1348***

***Escherichia/Shigella coli ASV−68***

***Oscillibacter uncl. ASV−138 raeggerthella uncl. ASV−1558***

***Pa***

***Akkermansia muciniphila ASV−49 Lachnospiraceae uncl. ASV−123***

***Oscillibacter uncl. ASV−366 elotrichaceae uncl. ASV−975***

***Erysip***

***Clostridium XlVa scindens ASV−1247 Erysipelotrichaceae uncl. ASV−764***

***Alistipes putredinis ASV−4***

***Oscillibacter uncl. ASV−221 chia/Shigella uncl. ASV−104 Firmicutes uncl. ASV−215 Anaerotruncus uncl. ASV−187 Faecalibacterium uncl. ASV−173***

***Escheri***

***Alistipes uncl. ASV−8***

***Lachnospiraceae uncl. ASV−55***

***Fusobacterium nucleatum ASV−213 Roseburia uncl. ASV−361***

***Pseudoflavonifractor uncl. ASV−4321***

***Alistipes uncl. ASV−135***

***Oscillibacter uncl. ASV−299 Ruminococcaceae uncl. ASV−854 Lactobacillus sakei ASV−2534 Ruminococcaceae uncl. ASV−1693***

***Clostridium XlVa uncl. ASV−856***

***Escherichia/Shigella uncl. ASV−10***

***Lachnospiraceae uncl. ASV−286 Alistipes uncl. ASV−6572***

***Ruminococcus2 uncl. ASV−1860***

***Rothia mucilaginosa ASV−2932 Clostridiales uncl. ASV−4153 Oscillibacter uncl. ASV−581***

***Pseudoflavonifractor uncl. ASV−418***

***Bacteroides uniformis ASV−2***

***Lachnospiraceae uncl. ASV−275 Propionispira uncl. ASV−428***

***Prevotella uncl. ASV−105 Oscillibacter uncl. ASV−126***

***Anaerotruncus uncl. ASV−187***

***Flavonifractor plautii ASV−93***

***Clostridium XlVa uncl. ASV−1489 Clostridium XlVa clostridioforme ASV−103***

***Parabacteroides merdae ASV−12***

***Oscillibacter uncl. ASV−366***

***Lachnospiraceae uncl. ASV−352 Clostridium XlVb uncl. ASV−744***

***Ruminococcaceae uncl. ASV−1078 Eisenbergiella uncl. ASV−519***

***Lachnospiraceae uncl. ASV−962***

***Bacteroides massiliensis ASV−22***

***Bacteroides uniformis ASV−2 Lachnospiraceae uncl. ASV−565 Oscillibacter uncl. ASV−157 Oscillibacter uncl. ASV−313 Oscillibacter uncl. ASV−299 Clostridium IV uncl. ASV−555 Blautia uncl. ASV−1501***

17)

18).

***Egg***

***Ali bacterium longum ASV−2943***

***erthella lenta ASV−829***

Colored

***Escherichia/Shigella uncl. ASV−75 Clostridiales uncl. ASV−914Bifid o stipes indistinctus ASV−140 Clostridiales uncl. ASV−1936 Anaerovorax uncl. ASV−2627 Clostridium XlVa uncl. ASV−630 Clostridium IV uncl. ASV−150***

***Alistipes indistinctus ASV−140***

***Faecalibacterium uncl. ASV−71***

***Ruminococcaceae uncl. ASV−4258 Clostridium IV leptum ASV−586 Ruminococcaceae uncl. ASV−754 Ruminococcaceae uncl. ASV−4501 Anaerovorax uncl. ASV−1730***

***Ruminococcaceae uncl. ASV−1194 Clostridiales uncl. ASV−585 Lachnospiraceae uncl. ASV−2409***

8.

***Ruminococcaceae uncl. ASV−488 Prevotella uncl. ASV−309 Blautia uncl. ASV−2160***

***Clostridium XlVa uncl. ASV−943 Eggerthella lenta ASV−713***

***Ruminococcaceae uncl. ASV−1442 Ruminococcaceae uncl. ASV−3717 Ruminococcaceae uncl. ASV−106***

randomized centralities of the network and ASV names in red highlight significantly higher

than random network importance (FDR corrected, Table S24).

25

Node PageRank index

*P*FDR≤0.05

0.010

0.008

0.006

0.004

0.002

0.000Contr/CD

CoCnDtr//UC

Contr/IBD

Contr/CD

Contr/UC

ConCtDr/I/BUDC

Contr/CD

Contr/UC

ConCtDr/I/BUDC

Contr/CD

Contr/UC

Contr/IBD

Contr/CD

Contr/UC

Contr/IBD

Contr/CD

Contr/UC

Contr/IBD

Contr/CD

Contr/UC

Contr/IBD

*P*FDR≤0.05

**Figure S15:** Network importance

highlighting differential abundance associations at each node as derived from *DESeq2*

Centralities range from the number of connections (degree), the position on the shortest paths

within the network (betweeness,

average neighborhood degree of any given vertex (k-nearest neighbor degree

***Clostridiales uncl. ASV−117***

CD/UC

CD/UC

CD/UC

CD/UC

Node degree

***Clostridiales uncl. ASV−117***

0.04

0.03

0.02

0.01

0.00Contr/CD

CoCnDtr//UC

Contr/IBD

Contr/CD

CoCnDtr//UC

Contr/IBD

Contr/CD

Contr/UC

ConCtDr/I/BUDC

Contr/CD

Contr/UC

ConCtDr/I/BUDC

Contr/CD

Contr/UC

CoCD/UC

ntr/IBD

Contr/CD

Contr/UC

CoCD/UC

ntr/IBD

Contr/CD

Contr/UC

CoCD/UC

ntr/IBD

***Alistipes putredinis ASV−4***

***Clostridium XVIII ramosum ASV−193***

***Flavonifractor plautii ASV−93***

***Alistipes putredinis ASV−4***

***Oscillibacter uncl. ASV−138 Clostridium XlVa clostridioforme ASV−103***

***Lachnospiraceae uncl. Flavonifractor plautii***

***ASV−55 ASV−93***

***Lachnospiraceae uncl. ASV−64 Clostridium XlVa clostridioforme ASV−103***

***Clostridium XVIII ramosum ASV−193 Oscillibacter uncl. ASV−138***

***Lachnospiraceae uncl. ASV−55 Oscillibacter uncl. ASV−403 Oscillibacter uncl. ASV−209***

***Rothia mucilaginosa ASV−2932 Clostridium XlVa uncl. ASV−171 Clostridium XlVa uncl. ASV−856***

***Clostridium XlVa uncl. ASV−266 Ruminococcaceae uncl. ASV−106 Lachnospiraceae uncl. ASV−3004 Clostridiales uncl. ASV−515 Clostridium XlVa uncl. ASV−856***

***Subdoligranulum uncl. ASV−20 Ruminococcaceae uncl. ASV−106 Oscillibacter uncl. ASV−126Pro pio hnospiraceae uncl. ASV−352***

***Clostridiales uncl. ASV−1936***

***Lac nibacterium acnes ASV−332***

***Lachnospiraceae uncl. ASV−64***

***Clostridiales uncl. ASV−914 Clostridiales uncl. ASV−914***

***Bacteroides uncl. ASV−1***

***Granulicatella uncl. ASV−1903***

***Roseburia uncl. ASV−361 Faecalibacterium uncl. ASV−270 Faecalibacterium uncl. ASV−566 Blautia uncl. ASV−753 Clostridiales uncl. ASV−1483 Clostridium XlVa uncl. ASV−1057 Oscillibacter uncl. ASV−774 Firmicutes uncl. ASV−283***

***Clostridium XlVa uncl. ASV−534 Prevotella uncl. ASV−101 Shewanella uncl. ASV−3026***

***Clostridiales uncl. ASV−1936 Oscillibacter uncl. ASV−209 Veillonella uncl. ASV−222 Clostridiales uncl. ASV−515 Eggerthella lenta ASV−829 Gemella sanguinis ASV−5679 Oscillibacter uncl. ASV−403***

***Clostridium XlVa uncl. ASV−1035***

***Clostridiales uncl. ASV−1483 Anaerovorax uncl. ASV−1730 Roseburia uncl. ASV−361***

***Faecalibacterium uncl. ASV−71***

***Clostridium XlVa uncl. ASV−534***

***Roseburia uncl. ASV−1981 Oscillibacter uncl. ASV−581***

***Prevotella uncl. Parasutterella uncl.***

***ASV−73 ASV−60***

***Veillonella uncl. ASV−582 Eggerthella lenta ASV−713***

***Firmicutes uncl. ASV−1083 Subdoligranulum uncl. ASV−1009***

***Blautia uncl. ASV−753 Ruminococcaceae uncl. ASV−757***

***Parasutterella uncl. ASV−24 Bacteroides uncl. ASV−132 Ruminococcus2 uncl. ASV−339 Lachnospiraceae uncl. ASV−273 Granulicatella uncl. ASV−1903***

***Blautia uncl. ASV−1501 nas phoceae ASV−2429 Parasutterella uncl. ASV−142***

***Halomo***

***Ruminococcaceae uncl. ASV−3619***

16),

***Oscillibacter uncl. ASV−1723 Ruminococcaceae uncl. ASV−757 Oscillibacter uncl. ASV−126 Clostridiales uncl. ASV−1705 Veillonella uncl. ASV−222***

measures

***Clostridium XlVa uncl. ASV−1057 Firmicutes uncl. ASV−1083 prococcus uncl. ASV−1190***

***Ruminococcaceae uncl. ASV−1194***

***Co***

***Blautia uncl. ASV−1501Lachn osp***

***Lachnos iraceae uncl. ASV−3004***

***piraceae uncl. ASV−3009 Granulicatella adiacens ASV−3475 Ruminococcaceae uncl. ASV−3619 Veillonella uncl. ASV−118 Firmicutes uncl. ASV−283 Firmicutes uncl. ASV−440***

***Eis***

***enbergiella uncl. ASV−519***

***Blautia hansenii ASV−599***

***Bacteroides uncl. ASV−74***

***Veillonella uncl. ASV−1015***

***Proteobacteria uncl. ASV−226 Faecalibacterium uncl. ASV−302 Bacteroides thetaiotaomicron ASV−161***

generalized importance (PageRank index and the

***Veillonella uncl. ASV−582***

***Ruminococcaceae uncl. ASV−1229 Erysipelotrichaceae uncl. ASV−1684 Clostridiales uncl. ASV−1705***

***Butyricicoccus uncl. ASV−2138***

***Ruminococcaceae uncl. ASV−854 Butyricimonas uncl. ASV−1110 Lachnospiraceae uncl. ASV−459***

***Alistipes shahii Faecalibacterium prausnitzii Faecalibacterium uncl.***

***ASV−31 ASV−85 ASV−94***

***Faecalibacterium uncl.***

***ASV−89***

***Lachnospiraceae uncl. ASV−116***

***Ruminococcaceae uncl. ASV−343 Ruminococcaceae uncl. ASV−2758***

Node KNN degree

25

20

15

10

5

***Streptococcus uncl. ASV−264 Oscillibacter uncl. ASV−533***

Node betweenness

0.25

0.20

0.15

0.10

0.05

0.00Contr/CD

***Odoribacter uncl. ASV−196 Coprobacillus uncl. ASV−100 Roseburia hominis ASV−191***

*P*FDR≤0.05

derived at the first follow-up time point

***Lachnospiraceae uncl. Alistipes putredinis Flavonifractor plautii***

***ASV−55 ASV−4 ASV−93***

Contr/UC

ConCtDr/I/BUDC

Contr/CD

Contr/UC

ConCtDr/I/BUDC

Contr/CD

Contr/UC

CoCD/UC

ntr/IBD

Contr/CD

Contr/UC

CoCD/UC

ntr/IBD

Contr/CD

Contr/UC

CD/UC

Contr/IBD

Contr/CD

Contr/UC

CD/UC

Contr/IBD

Contr/CD

Contr/UC

CD/UC

Contr/IBD

0Contr/CD

CoCnDtr//UC

Contr/IBD

Contr/CD

Contr/UC

ConCtDr/I/BUDC

Contr/CD

Contr/UC

ConCtDr/I/BUDC

Contr/CD

Contr/UC

CoCD/UC

ntr/IBD

Contr/CD

Contr/UC

CoCD/UC

ntr/IBD

Contr/CD

Contr/UC

CoCD/UC

ntr/IBD

Contr/CD

Contr/UC

CoCD/UC

ntr/IBD

***Dorea uncl. ASV−1284 Oxalobacter uncl. ASV−408 Lachnospiraceae uncl. ASV−123 Oscillibacter uncl. ASV−157 Clostridiales uncl. ASV−816***

***Firmicutes uncl. ASV−561***

***Lachnospiraceae uncl. ASV−123 Oscillibacter uncl. ASV−157***

***Ruminococcaceae uncl. ASV−1229 Rothia mucilaginosa ASV−2932 Clostridium IV uncl. ASV−6760***

***Propionibacterium acnes ASV−332***

***Alistipes shahii ASV−31***

***Oscillibacter uncl. ASV−313***

***Lachnospiraceae uncl. ASV−468***

***Pseudoflavonifractor uncl. ASV−773***

***Alistipes onderdonkii ASV−13 Oscillibacter uncl. ASV−299 Oscillibacter uncl. ASV−366***

***Anaerovorax uncl. ASV−1730 Alistipes uncl. ASV−6572 Oscillibacter uncl. ASV−126***

***Clostridiales uncl. ASV−1384***

***Subdoligranulum uncl. ASV−20***

***Ruminococcaceae uncl. ASV−540 Erysipelotrichaceae uncl. ASV−975 Oscillibacter uncl. ASV−414 Oscillibacter uncl. ASV−156***

***Lactobacillus gasseri ASV−714***

***Ruminococcus uncl. ASV−122 Clostridium XlVa uncl. ASV−630 Clostridium XlVb uncl. ASV−744 Ruminococcaceae uncl. ASV−1439***

***Lachnospiraceae uncl. ASV−273 Ruminococcaceae uncl. ASV−4279 Lachnospiraceae uncl. ASV−3009 Collinsella uncl. ASV−2816***

***Lachnospiraceae uncl. ASV−280***

***Ruminococcaceae uncl. ASV−349 Ruminococcaceae uncl. ASV−4245 Firmicutes uncl. ASV−440 Clostridiales uncl. ASV−1483***

***Subdoligranulum uncl. ASV−69***

***Oscillibacter uncl. ASV−366***

***Ruminococcaceae uncl. ASV−340 Clostridium XlVa uncl. ASV−909***

***Alistipes indistinctus ASV−140 Eggerthella lenta ASV−713***

***Alistipes uncl. ASV−8***

***Clostridium XlVa uncl. ASV−1348 Clostridium XlVa uncl. ASV−665 Alistipes uncl. ASV−135 Clostridium XlVa uncl. ASV−1035 Lachnospiraceae uncl. ASV−244***

***Clostridium XlVa symbiosum ASV−689 Clostridium IV uncl. ASV−150***

***Ruminococcaceae uncl. ASV−1194 Lachnospiraceae uncl. ASV−352 Bacteroides uncl. ASV−492 Bacteroides uncl. ASV−385 Clostridium XlVa uncl. ASV−1016 Clostridium XlVa uncl. ASV−630 Eggerthella uncl. ASV−667***

***Clostridium XlVa uncl. ASV−171***

***Eggerthella uncl. ASV−667***

***Alistipes uncl. ASV−8***

***Ruminococcaceae uncl. ASV−2099 Firmicutes uncl. ASV−283 Lachnospiraceae uncl. ASV−439***

*P*FDR≤0.05

***Escherichia/Shigella uncl. ASV−205 Oscillibacter uncl. ASV−533 Oscillibacter uncl. ASV−221***

***Alistipes uncl. ASV−6572 Anaerovorax uncl. ASV−1730***

***Escherichia/Shigella uncl. Acidaminococcus uncl.***

***ASV−27 ASV−33***

***Lachnospiraceae uncl. ASV−381***

***Clostridium IV uncl. ASV−2063 Butyricicoccus uncl. ASV−2138 Eggerthella lenta ASV−829***

17)

18).

***Clostridium XVIII uncl. ASV−427 Flavonifractor uncl. ASV−691 Oxalobacter uncl. ASV−408***

***Clostridiales uncl. ASV−1705***

***Ruminococcaceae uncl. ASV−1194 Ruminococcaceae uncl. ASV−2758 Oscillibacter uncl. ASV−1898***

***Alistipes shahii ASV−31***

***Coriobacteriaceae uncl. ASV−2789 Roseburia uncl. ASV−1063***

***Acidaminococcus uncl. ASV−33***

***Desulfovibrio piger ASV−451***

***Lachnospiraceae uncl. ASV−962 Ruminococcaceae uncl. ASV−965 Propionispira uncl. ASV−428 Clostridium IV leptum ASV−586***

Colored

***Anaerovorax uncl. ASV−2801 Ruminococcus2 uncl. ASV−2297 Faecalibacterium uncl. ASV−235 Barnesiella intestinihominis ASV−125***

***Clostridium XlVa scindens ASV−1247***

***Oscillibacter uncl.***

***ASV−83***

***Clostridiales uncl. ASV−177 Ruminococcus uncl. ASV−1049***

***Flavonifractor plautii***

***ASV−93***

***Clostridium IV uncl. ASV−546***

***Veillonella uncl. ASV−1015 Veillonella uncl. ASV−118***

and

***Bacteroides uniformis ASV−2***

***Roseburia uncl. ASV−361***

***Clostridium XVIII uncl. ASV−427 Oscillibacter uncl. ASV−1625***

8.

***Ruminococcaceae uncl. ASV−3619 Catabacter uncl. ASV−1630***

boxes highlight significant associations in KIN and external cohorts with IBD pathologies or

IBD onset. Significance of centralities is derived from Z-test against a large collection of

26

Node PageRank index

*P*FDR≤0.05

0.008

0.006

0.004

0.002

0.000Contr/CD

Contr/UC

ConCtDr/I/BUDC

Contr/CD

Contr/UC

Contr/IBD

Contr/CD

Contr/UC

Contr/IBD

Contr/CD

Contr/UC

Contr/IBD

Contr/CD

Contr/UC

Contr/IBD

Contr/CD

Contr/UC

Contr/IBD

Conttrr//CUDC

Contr/IBD

*P*FDR≤0.05

**Figure S16:** Network importance measures derived at the second follow-up time point and

highlighting differential abundance associations at each node as derived from *DESeq2*

Centralities range from the number of connections (degree), the position on the shortest paths

***Clostridiales uncl. ASV−117***

CD/UC

CD/UC

CD/UC

CD/UC

CD/UC

CD/UC

Node degree

***Clostridium XVIII ramosum ASV−193***

randomized centralities of the network and ASV names in red highlight significantly higher

than random network importance (FDR corrected Table S24).

0.04

0.03

0.02

0.01

0.00Contr/CD

Contr/UC

CD/UC

Contr/IBD

Contr/CD

Contr/UC

CD/UC

Contr/IBD

Conttrr//CUDC

CD/UC

Contr/IBD

Conttrr//CUDC

CD/UC

Contr/IBD

Contr/CD

CoCnDtr//UC

Contr/IBD

Contr/CD

CoCnDtr//UC

Contr/IBD

Contr/CD

Contr/UC

ConCtDr/I/BUDC

***Clostridium XVIII ramosum ASV−193 Alistipes putredinis ASV−4 Clostridiales uncl. ASV−914 Firmicutes uncl. ASV−283***

***Flavonifractor plautii ASV−93 Clostridium XlVa clostridioforme ASV−103***

***Clostridiales uncl. ASV−117 Clostridiales uncl. ASV−914***

***Clostridiales uncl. ASV−515 Flavonifractor plautii ASV−93***

***Alistipes putredinis ASV−4***

***Clostridium XlVa uncl. ASV−171***

***Blautia uncl. ASV−753***

***Clostridium XlVa clostridioforme ASV−103 Clostridium XlVa uncl. ASV−266 Clostridium XlVa uncl. ASV−534 Oscillibacter uncl. ASV−403***

***Mogibacterium uncl. ASV−3131***

***Oscillibacter uncl. ASV−126 Oscillibacter uncl. ASV−138 Coprococcus uncl. ASV−1347 Firmicutes uncl. ASV−440***

***Bacteroides uncl. ASV−1***

***Firmicutes uncl. ASV−561***

***Firmicutes uncl. ASV−561 Clostridium XlVa uncl. ASV−856 Faecalibacterium uncl. ASV−270***

***Clostridium XlVa uncl. ASV−856 Anaerotruncus uncl. ASV−2444 Firmicutes uncl. ASV−283***

***Haemophilus parainfluenzae ASV−97***

***Clostridiales uncl. ASV−515***

***Firmicutes uncl. ASV−440 Clostridium XlVa uncl. ASV−1057***

***Blautia uncl. ASV−753 Ruminococcaceae uncl. ASV−2758***

***Oscillibacter uncl. ASV−126 Clostridium XlVa uncl. ASV−489 Romboutsia uncl. ASV−576 Oscillibacter uncl. ASV−138***

***Subdoligranulum uncl. ASV−20 Ruminococcaceae uncl. ASV−106 Roseburia uncl. ASV−361 Oscillibacter uncl. ASV−403***

***Lachnospiraceae uncl. ASV−55***

***Faecalibacterium uncl. ASV−302 Roseburia uncl. ASV−1125***

***Rothia mucilaginosa ASV−2932***

***Blautia hansenii ASV−599 Lactobacillus gasseri ASV−714 Clostridium XlVa uncl. ASV−1254 Clostridiales uncl. ASV−1483***

***Lachnospiraceae uncl. ASV−116***

***Oscillibacter uncl. ASV−1625***

***Lachnospiraceae uncl.***

***Lachnospiraceae uncl.***

***ASV−55 ASV−64***

***Butyricimonas uncl. ASV−1147***

***Veillonella uncl. ASV−222***

***Lachnospiraceae uncl. Faecalibacterium uncl.***

***Prevotella uncl. Klebsiella uncl.***

***ASV−64 ASV−94 ASV−73 ASV−40***

***Propionibacterium acnes ASV−332 Clostridium XlVa uncl. ASV−489 Eisenbergiella uncl. ASV−519 Blautia uncl. ASV−677***

***Coprococcus uncl. ASV−1347***

***Roseburia uncl. ASV−361 Ruminococcaceae uncl. ASV−106 Mogibacterium uncl. ASV−3131***

***Clostridium XlVa uncl. ASV−1057***

***Oscillibacter uncl. ASV−1723 Lachnospiraceae uncl. ASV−1773 Butyricicoccus uncl. ASV−2138***

***Escherichia/Shigella coli ASV−181***

***Lachnospiraceae uncl. ASV−116***

***Eisenbergiella uncl. ASV−519 Victivallis uncl. ASV−3285***

***Ruminococcus2 uncl. ASV−803***

***Ruminococcaceae uncl. ASV−592 Clostridium XlVa symbiosum ASV−689 Eggerthella lenta ASV−713***

***Parasutterella uncl. Oscillibacter uncl.***

***ASV−24 ASV−83***

***Eggerthella lenta ASV−829 Ruminococcaceae uncl. ASV−971***

***Butyricimonas uncl. ASV−1551 Ruminococcaceae uncl. ASV−1693***

***Blautia uncl. ASV−1902***

***Anaerotruncus uncl. ASV−1216 Paraeggerthella uncl. ASV−1558 Clostridiales uncl. ASV−1705***

***Lachnospiraceae uncl. ASV−35***

***Diaphorobacter nitroreducens ASV−1941***

***Parasutterella uncl. ASV−142 Clostridium XlVa uncl. ASV−2670***

***Paraeggerthella uncl. ASV−1558 Lachnospiraceae uncl. ASV−273 Clostridiales uncl. ASV−1705***

***Oscillibacter uncl. Faecalibacterium uncl. Faecalibacterium uncl.***

***ASV−82 ASV−89 ASV−94***

***Faecalibacterium uncl. ASV−71***

***Haemophilus parainfluenzae***

***ASV−97***

***Ruminococcus2 uncl. ASV−339 Clostridiales uncl. ASV−1483 Faecalibacterium prausnitzii ASV−325 Ruminococcaceae uncl. ASV−2758 Blautia hansenii ASV−599***

***Ruminococcaceae uncl. ASV−340 Lachnospiraceae uncl. ASV−352 Clostridium XlVa uncl. ASV−534 Clostridiales uncl. ASV−816***

***Ruminococcaceae uncl. ASV−854***

***Parasutterella uncl. ASV−60***

***Erysipelotrichaceae uncl. ASV−975***

***Blautia uncl. ASV−1087***

*P*FDR≤0.05

***Clostridium XVIII uncl. ASV−111 Clostridium IV uncl. ASV−150***

Node KNN degree

25

20

15

10

5

***Erysipelotrichaceae uncl. ASV−987***

***Clostridium XlVa uncl. ASV−171 Clostridium XVIII ramosum ASV−193***

Contr/UC

C CD/UC

ontr/IBD

Contr/CD

Contr/UC

C CD/UC

ontr/IBD

Conttrr//CUDC

C CD/UC

ontr/IBD

Conttrr//CUDC

C CD/UC

ontr/IBD

Contr/CD

CoCnDtr//UC

Contr/IBD

Contr/CD

CoCnDtr//UC

Contr/IBD

Contr/CD

Contr/UC

CoCntDr//IUBCD

*P*FDR≤0.05

30

0Contr/CD

Contr/UC

CoCntDr//IUBCD

Contr/CD

Contr/UC

C CD/UC

ontr/IBD

Contr/CD

Contr/UC

C CD/UC

ontr/IBD

Contr/CD

Contr/UC

C CD/UC

ontr/IBD

Contr/CD

Contr/UC

C CD/UC

ontr/IBD

Contr/CD

Contr/UC

C CD/UC

ontr/IBD

Conttrr//CUDC

C CD/UC

ontr/IBD

Node betweenness

0.15

0.10

0.05

0.00Contr/CD

***Oscillibacter uncl. ASV−221 Bacteroides uncl. ASV−29***

***Subdoligranulum uncl. ASV−20 Oscillibacter uncl. ASV−126***

***Ruminococcaceae uncl. ASV−1439 Parabacteroides merdae ASV−12***

***Lachnospiraceae uncl. ASV−55***

***Roseburia uncl. ASV−361***

***Odoribacter uncl. ASV−196 Erysipelotrichaceae uncl. ASV−975 Ruminococcaceae uncl. ASV−1430***

***Streptophyta uncl. ASV−1114***

***Ruminococcaceae uncl. ASV−540 Clostridium XlVa symbiosum ASV−689 Holdemania uncl. ASV−1596 Clostridiales uncl. ASV−816***

***Oscillibacter uncl. ASV−138 Citrobacter uncl. ASV−167 Lachnospiraceae uncl. ASV−1985***

***Ruminococcaceae uncl. ASV−340 Clostridiales uncl. ASV−914 Blautia hansenii ASV−599 Clostridiales uncl. ASV−816 Odoribacter uncl. ASV−196***

***Oscillibacter uncl. ASV−299***

***Faecalibacterium uncl. ASV−89***

***Clostridium XlVa uncl. ASV−630 Pseudoflavonifractor uncl. ASV−911***

***Alistipes shahii ASV−31***

***Alistipes putredinis ASV−4***

***Bilophila uncl. ASV−1264 Oscillibacter uncl. ASV−313 Clostridium XlVa uncl. ASV−1035 Roseburia uncl. ASV−1063***

***Butyricicoccus uncl. ASV−2138***

***Ruminococcaceae uncl. ASV−211 Ruminococcaceae uncl. ASV−349***

***Lachnospiraceae uncl. ASV−381 Propionibacterium acnes ASV−332 Butyricicoccus uncl. ASV−2138***

***Odoribacter uncl. ASV−1324***

***Anaerotruncus uncl. ASV−2444 Lachnospiraceae uncl. ASV−352 Butyricicoccus uncl. ASV−2174***

***Clostridiales uncl. ASV−259***

***Escherichia/Shigella uncl. ASV−10***

***Ruminococcaceae uncl. ASV−1205 Coprobacillus uncl. ASV−100***

***Lactococcus lactis ASV−1292 Ruminococcaceae uncl. ASV−2773***

***Bacteroides uniformis ASV−2***

***Mogibacterium uncl. ASV−3131***

***Ruminococcaceae uncl. ASV−2252 Blautia hansenii ASV−599 Eggerthella lenta ASV−829 Clostridium XlVa uncl. ASV−534 Blautia uncl. ASV−1501***

***Intestinimonas uncl. ASV−649***

***Clostridium XlVa uncl. ASV−489 Lachnospiraceae uncl. ASV−1477 Blautia uncl. ASV−1715***

***Coprococcus uncl. ASV−1347***

***Ruminococcaceae uncl. ASV−971 Proteobacteria uncl. ASV−2403***

***Akkermansia muciniphila Subdoligranulum uncl.***

***Alistipes uncl.***

***ASV−49 ASV−69 ASV−8***

***Veillonella uncl. ASV−222 Blautia uncl. ASV−1501***

***Clostridium XlVa uncl. ASV−1489***

***Roseburia uncl. ASV−361 Clostridiales uncl. ASV−515***

***Ruminococcaceae uncl. ASV−3370 Clostridiales uncl. ASV−117***

***Clostridium IV uncl. ASV−546 Clostridium XVIII uncl. ASV−427 Lachnospiraceae uncl. ASV−123***

***Lachnospiraceae uncl. ASV−64***

***Ruminococcus2 uncl. ASV−1860 Oscillibacter uncl. ASV−774***

***Lachnospiraceae uncl. ASV−352***

***Flavonifractor plautii ASV−93***

***Eggerthella lenta ASV−713 Clostridium XlVa uncl. ASV−171***

***Clostridium XlVa uncl. ASV−856 Eisenbergiella uncl. ASV−519 Oscillibacter uncl. ASV−157***

***Coprobacillus uncl. ASV−100 Candidatus Saccharibacteria uncl. ASV−3225***

***Veillonella uncl. ASV−118 Lachnospiraceae uncl. ASV−1008 Alistipes uncl. ASV−344***

***Acidaminococcus uncl. Oscillibacter uncl.***

***ASV−33 ASV−83***

***Oscillibacter uncl. ASV−1723 Blautia uncl. ASV−2160***

***Clostridium XlVa uncl. ASV−1254 Clostridium XlVa uncl. ASV−898 Ruminococcaceae uncl. ASV−2116 Clostridiales uncl. ASV−1384 Clostridium XlVb uncl. ASV−744 Anaerotruncus uncl. ASV−2444***

***Lachnospiraceae uncl. ASV−286***

***Blautia uncl. ASV−753 Clostridiales uncl. ASV−1936 Lachnospiraceae uncl. ASV−275***

8.

***Ruminococcaceae uncl. ASV−2099 Clostridium XlVa uncl. ASV−898 Clostridium XlVa uncl. ASV−571 Ruminococcaceae uncl. ASV−1920 Clostridiales uncl. ASV−1384***

***Blautia uncl. ASV−677 Clostridiales uncl. ASV−4153 Clostridiales uncl. ASV−1483***

***Erysipelotrichaceae uncl. ASV−987***

***Firmicutes uncl. ASV−440***

within the network (betweeness,

16),

generalized importance (PageRank index and the

17)

average neighborhood degree of any given vertex (k-nearest neighbor degree

18).

Colored

27

boxes highlight significant associations in KIN and external cohorts with IBD pathologies or IBD onset. Significance of centralities is derived from Z-test against a large collection of randomized centralities of the network and ASV names in red highlight significantly higher than random network importance (FDR corrected Table S24).

*Bacteroidetes Proteobacteria*

*Firmicutes Verrucomicrobia*

*Candidatus Saccharibacteria*

0.50

0.75

1.00

posit nega

Control CD

**A**

**B**

Control UC

**C**

CD UC

*Actinobacteria Fusobacteria*

0.10

0.25

ive tive

*Bacteroidetes Proteobacteria Firmicutes Verrucomicrobia Actinobacteria*

*Fusobacteria Bacteria unclassified*

*Cyanobacteria.Chloroplast Candidatus Saccharibacteria*

0.10

0.25

0.50

0.75

1.00

positive negative

*Bacteroidetes Proteobacteria Firmicutes Verrucomicrobia Actinobacteria*

*Cyanobacteria.Chloroplast Bacteria unclassified Candidatus Saccharibacteria Lentisphaerae*

0.10

0.25

0.50

0.75

1.00

positive negative

**D**

Control IBD

**Baseline Follow-up 1 Follow-up 2**

**Figure S17:** Spiec-Easi networks of baseline samples, follow-up 1, and follow-up 2. Bacterial nodes highlight significant differentially abundant ASVs in the network. **(A)** Bacteria not showing any differential abundance patterns between CD patients and healthy controls are signified via (●), bacteria overabundant in CD via (■) and bacteria more abudnant in controls

are signified via (►). **(B)** Bacteria not showing any differential abundance patterns between

UC patients and healthy controls are signified via (●), bacteria overabundant in UC via (★) and bacteria more abudnant in controls are signified via (►). **(C)** Bacterial nodes highlight significant differentially abundant ASVs in the network. Bacteria not showing any differential abundance patterns between UC patients and healthy controls are signified via (●), bacteria overabundant in UC via (★) and bacteria more abudnant in CD are signified via (■). **(D)** Bacterial nodes highlight significant differentially abundant ASVs in the network. Bacteria not showing any differential abundance patterns between UC patients and healthy controls are signified via (●), bacteria overabundant in healthy controls via (►) and bacteria more abudnant in IBD (CD+UC) are signified via (■).

**A 4-node Graphlet distance**

**Relative edge sharing distance**

1.0

Control CD

UC

IBD (CD / UC / uIBD)

Full cohort

Germany-BL Germany-F1 Germany-F2

Sweden Malta naive

Malta remission

**Stress:** 16.621

Control CD

UC

IBD (CD / UC / uIBD)

Full cohort

Germany-BL Germany-F1 Germany-F2

Sweden Malta naive

Malta remission

**Stress:** 1.868

0.4

0.5

0.2

0.0

**NMDS2**

**NMDS2**

0.0

−0.5

−0.2

−1.0

−0.4

−1.5

**B**

−1.0 −0.5 0.0 0.5 1.0

**NMDS 1**

**Network diameter**

−0.4 −0.2 0.0 0.2 0.4

**NMDS1**

**Network density**

5 0.030

P=0.7505

P=0.0995

4 0.025

0.020

3

0.015

2

0.010

1 0.005

0

**Network assortativity**

0.000

**Network size**

0.4 15000

P=0.2485

P=0.6167

0.3

10000

0.2

5000

0.1

0.0 0

**Network radius**

P=0.0441

8

0.0005

**Natural connectivity**

P=0.0111

0.0004

6

0.0003

4

0.0002

2

0.0001

0 **Full**

**Contr. CD UC IBD**

0.0000

**Full**

**Contr. CD UC IBD**

**time points/ cohort**

**(CD/UC)**

**time points/ cohort**

**(CD/UC)**

**Figure S18: (A)** Network similarity of disease and time point specific sub networks, as well as subnetworks of external cohorts (Malta, Sweden) based on graphlet distance ^19^ and relative

edge sharing distance, displayed via NMDS. Networks show a clear compositional difference between healthy and diseased networks (IBD) as based on graphlet distance (Control *vs.* IBD (incl. IBD networks): *F*_1,18_=2.4144, *P*=0.0412, *R^2^*=0.1183, *adj. R^2^*=0.0693; PERMANOVA)

and relative edge sharing distance (KINDRED only- Control *vs*. IBD: *F*_1,7_=1.0775, *P*=0.0621, *R^2^*=0.1334, *adj. R^2^*=0.0096; all cohorts (Contr., CD, UC)- Control *vs*. IBD: *F*_1,12_=1.1096, *P*=0.0935, *R^2^*=0.0846, *adj. R^2^*=0.0084; all cohorts (Contr., CD, UC, IBD)- Control *vs*. IBD (incl. general IBD networks): *F*_1,18_=1.1201, *P*=0.0853, *R^2^*=0.0586, *adj. R^2^*=0.0063; PERMANOVA). **(B)** Global network characteristics derived from KINDRED, Malta IBD- naive, Malta IBD-remission, and Sweden-SIC cohorts. Significance is based on Wilcoxon tests between healthy control based networks and networks derived from diseased individuals (CD, UC, IBD). Assortativity ^20^, diameter, radius, and size ^21^, density ^22^, natural connectivity

^23^. Full time point specific (KINDRED BL, KINDRED F1, KINDRED F2) and cohort specific (Malta IBD-naive incl. controls, Malta IBD-remission incl. controls, Sweden-SIC cohorts).

*AS V_6−B acter oi desu nif orm i s AS V_84−P ar apre votel la uncl .*

*AS V_90−P ar abacte roi des uncl. AS V_567− Subdol igr anul um uncl .*

**KIN**

**KIN**

**KIN**

**KIN**

**MALTA MALTA**

**Sweden**

*AS V_34−B acte roi des uncl. AS V_67−B acte roi des uncl.*

**BL F1**

**F2 Comb.**

**naive**

**rem.**

*AS V_3−B acter oi desd orei AS V_70−P ar asutt er ell aun cl. AS V_30−B acte roi des uncl.*

*AS V_21−B acte roi des uncl. AS V_143− Ali sti pes uncl.*

*AS V_321− Faecal ibact er ium un cl. AS V_1245 −Rum in ococcaceae uncl. AS V_57−B acte roi des uncl.*

*AS V_5−E scher ichi a/S higel la uncl .*

*AS V_551− Copr obact er uncl . AS V_1844 −Bur khol deri a uncl. AS V_385− Bact eroi des uncl.*

*AS V_174− Subdol igr anul um uncl . AS V_29−B acte roi des uncl.*

*AS V_107− Bact eroi des vulga tus AS V_425− Faecal ibact er ium un cl.*

*AS V_36−B acte roi des thet aio taom icr on AS V_10−E scher ichi a/S higel la uncl .*

*AS V_890− Blau ti auncl .*

*AS V_32−D ial ist er i nvisus AS V_45−P hascol arct obact er ium un cl.*

*AS V_74−B acte roi des uncl. AS V_651− Copr ococcus uncl .*

*AS V_1898 −Osci ll ibact er uncl . AS V_315− Clo str idi um sensu st ri cto uncl .*

*AS V_132− Bact eroi des uncl. AS V_23−P ar abacte roi des dist asoni s*

*AS V_71−F aecali bact eri um uncl . AS V_47−B acte roi des fr agil is*

*AS V_164− Par abact ero ides uncl. AS V_314− Faecal ibact er ium un cl. AS V_153− Par asutt er ell au ncl.*

*AS V_649− Int esti nim onas uncl . AS V_610− Lachnospi ra ceaeu ncl.*

*AS V_829− Egger th ell al enta AS V_267− Pseudof la vonif ra ctor un cl.*

*AS V_126− Osci lli bact er uncl . AS V_9−B acter oi desu ncl.*

*AS V_46−E scher ichi a/S higel la col i*

*AS V_17−D ial ist er i nvisus AS V_1035 −Cl ostr idi um X lVa uncl .*

*AS V_494− Clo str idi um I V uncl. AS V_100− Copr obaci ll usun cl.*

*AS V_49−A kker mansi a muci niph ila AS V_81−A li sti pesf in egoldi i AS V_8−A li sti pesun cl.*

*AS V_217− Faecal ibact er ium un cl. AS V_152− Faecal ibact er ium un cl.*

*AS V_12−P ar abacte roi des mer dae*

*AS V_189− Ali sti pes massi li ensis AS V_98−A naer osti pes hadr us*

*AS V_38−B acte roi des uncl. AS V_671− Clo str idi ales uncl .*

*AS V_22−B acte roi des massi li ensis AS V_417− Subdol igr anul um uncl .*

*AS V_576− Rom bout sia uncl . AS V_54−D ial ist er i nvisus*

*AS V_62−B acte roi des thet aio taom icr on*

*AS V_914− Clo str idi ales uncl . AS V_2−B acter oi desu nif orm i s*

*AS V_104− Escher ichi a/S hige lla uncl . AS V_1476 −Bla uti aun cl.*

*AS V_413− Faecal ibact er ium un cl. AS V_136− Bact eroi des uncl. AS V_79−P ar asutt er ell aun cl.*

*AS V_933− But yri cicoccus uncl . AS V_142− Par asutt er ell au ncl.*

*AS V_1278 −Faecal ibact er ium u ncl.*

*AS V_365− Faecal ibact er ium un cl. AS V_2773 −Rum in ococcaceae uncl. AS V_1−B acter oi desu ncl.*

*AS V_667− Egger th ell aun cl. AS V_312− Rum ino coccaceae uncl. AS V_244− Lachnospi ra ceaeu ncl.*

*AS V_44−B acte roi des cell ulosi lyt icus AS V_69−S ubdol igr anul um uncl .*

*AS V_60−P ar asutt er ell aun cl. AS V_221− Osci lli bact er uncl .*

*AS V_26−S utt er ell aw adsw ort hensi s AS V_382− Faecal ibact er ium un cl.*

*AS V_285− Blau ti auncl . AS V_65−P ar abacte roi des dist asoni s*

*AS V_408− Oxal obact er uncl . AS V_157− Osci lli bact er uncl . AS V_353− Dor ea longi cat ena*

*AS V_28−E scher ichi a/S higel la col i AS V_222− Veil lon ell auncl .*

*AS V_196− Odor ib acter uncl.*

*AS V_736− Anaer ovor ax uncl. AS V_18−B acte roi des xylani solven s*

*AS V_56−P ar abacte roi des uncl. AS V_193− Clo str idi um XV II I r amo sum AS V_214− Col lin sell aae rof acie ns*

*AS V_24−P ar asutt er ell aun cl. AS V_7−B acter oi desd orei AS V_840− Fir m icut esun cl.*

*AS V_41−P ar abacte roi des uncl. AS V_713− Egger th ell al enta*

*AS V_15−D ial ist er i nvisus AS V_66−B acte roi des uncl.*

*AS V_202− Ali sti pes uncl. AS V_83−O scil li bact er uncl .*

*AS V_284− Int esti nib acter uncl. AS V_268− Rosebu ri af aeci s*

*AS V_33−A cidam i nococcus uncl. AS V_43−A li sti pesf in egoldi i*

*AS V_293− Rosebu ri ai nuli nivo rans AS V_753− Blau ti auncl .*

*AS V_13−A li sti peso nder donkii AS V_161− Bact eroi des thet ai otaom i cron AS V_119− Rum ino coccaceae uncl.*

*AS V_835− Rum ino coccaceae uncl. AS V_479− Osci lli bact er uncl .*

*AS V_523− Faecal ibact er ium un cl. AS V_427− Clo str idi um XV II I uncl .*

*AS V_366− Osci lli bact er uncl . AS V_403− Osci lli bact er uncl .*

*AS V_394− Col lin sell aae rof acie ns AS V_519− Eisen ber giel la uncl.*

*AS V_35−L achnospi race aeun cl. AS V_275− Lachnospi ra ceaeu ncl. AS V_1700 −Cl ostr idi um I V uncl.*

*AS V_497− Lachnospi ra ceaeu ncl. AS V_458− Rum ino coccaceae uncl.*

*AS V_170− Rosebu ri ai nte stin ali s AS V_160− Escher ichi a/S hige lla uncl .*

*AS V_39−B acte roi des uncl.*

*AS V_159− Bil ophi la wadsw or thi a AS V_302− Faecal ibact er ium un cl. AS V_300− Lachnospi ra ceaeu ncl.*

*AS V_873− Clo str idi um sensu st ri cto uncl . AS V_438− Pseudof la vonif ra ctor un cl.*

*AS V_282− Bil ophi la uncl. AS V_292− Rum ino coccaceae uncl. AS V_808− Blau ti auncl .*

*AS V_233− Rum ino coccusun cl. AS V_187− Anaer ot run cusuncl .*

*AS V_89−F aecali bact eri um uncl . AS V_173− Faecal ibact er ium un cl. AS V_16−A li sti pesu ncl.*

*AS V_803− Rum ino coccus2u ncl. AS V_869− Er ysipel otr ich aceae uncl.*

*AS V_219− Rum ino coccaceae uncl. AS V_102− Odor ib acter uncl. AS V_958− Osci lli bact er uncl .*

*AS V_53−B acte roi des ovatus AS V_55−L achnospi race aeun cl.*

*AS V_240− Faecal ibact er ium un cl. AS V_117− Clo str idi ales uncl .*

*AS V_620− Clo str idi um Xl Va uncl . AS V_606− Blau ti auncl .*

*AS V_106− Rum ino coccaceae uncl. AS V_255− Haem ophi lus par ainf lu enzae*

*AS V_692− Faecal ibact er ium un cl. AS V_171− Clo str idi um Xl Va uncl .*

*AS V_82−O scil li bact er uncl . AS V_689− Clo str idi um Xl Va sym biosum*

*AS V_25−B acte roi des uncl. AS V_412− Faecal ibact er ium un cl.*

*AS V_4−A li sti pespu tr edin is AS V_37−B ar nesiel la uncl .*

*AS V_64−L achnospi race aeun cl. AS V_27−E scher ichi a/S higel la uncl . AS V_1087 −Bla uti aun cl.*

*AS V_14−B acte roi des vulgat us AS V_350− Odor ib acter splanch nicus*

*AS V_395− Blau ti auncl . AS V_239− Faecal ibact er ium un cl. AS V_1008 −Lachnospi r aceae uncl.*

*AS V_103− Clo str idi um Xl Va clost r idi ofor m e AS V_907− Clo str idi ales I ncert ae Sedi sX II un cl.*

*AS V_778− Lachnospi ra ceaeu ncl. AS V_59−B acte roi des fr agil is*

*AS V_435− Lachnospi ra ceaeu ncl. AS V_854− Rum ino coccaceae uncl.*

*AS V_85−F aecali bact eri um pr ausni tzi i*

*AS V_11−D ial ist er i nvisus AS V_845− Dor ea uncl.*

*AS V_48−L achnospi race aeun cl. AS V_108− Bact eroi des ovatu s*

*AS V_1076 −Rum in ococcaceae uncl. AS V_72−B acte roi des cell ulosi lyt icus*

*AS V_979− Rum ino coccaceae uncl. AS V_368− Str ept ococcus sali var ius*

*AS V_392− Col lin sell aae rof acie ns AS V_31−A li sti pessh ahii*

*AS V_344− Ali sti pes uncl. AS V_113− Escher ichi a/S hige lla col i*

*AS V_150− Clo str idi um I V uncl. AS V_97−H aem ophi lus par ainf lue nzae*

*AS V_1469 −Lachnospi r aceae uncl. AS V_237− Bact eroi des thet ai otaom i cron*

*AS V_2063 −Cl ostr idi um I V uncl. AS V_93−F lavoni fr act or pl auti i*

*AS V_332− Pr opion ibact er ium acn es AS V_422− Fir m icut esun cl.*

*AS V_192− Bil ophi la uncl. AS V_262− Par abact ero ides uncl. AS V_286− Lachnospi ra ceaeu ncl. AS V_50−B acte roi des uncl.*

*AS V_328− Lachnospi ra ceaeu ncl. AS V_1046 −Lachnospi r aceae uncl. AS V_752− Bact eroi des uncl.*

*AS V_947− But yri cicoccus uncl . AS V_1114 −St rept ophyt a uncl.*

*AS V_856− Clo str idi um Xl Va uncl .*

*AS V_774− Osci lli bact er uncl . AS V_1180 −Osci ll ibact er uncl . AS V_474− Fir m icut esun cl.*

*AS V_943− Clo str idi um Xl Va uncl . AS V_448− Fusicat eni bacte r uncl.*

*AS V_495− Blau ti auncl . AS V_1118 −Osci ll ibact er uncl .*

*AS V_669− Rum ino coccaceae uncl. AS V_140− Ali sti pes indi sti nctus*

*AS V_1597 −Rum in ococcaceae uncl. AS V_191− Rosebu ri aho mi nis*

*AS V_163− Ali sti pes uncl. AS V_468− Lachnospi ra ceaeu ncl.*

*AS V_116− Lachnospi ra ceaeu ncl. AS V_75−E scher ichi a/S higel la uncl .*

*AS V_185− Anaer ost ipes hadr us AS V_215− Fir m icut esun cl.*

*AS V_339− Rum ino coccus2u ncl. AS V_96−S tr ept ococcus sali vari us*

*AS V_270− Faecal ibact er ium un cl. AS V_352− Lachnospi ra ceaeu ncl. AS V_145− Aest uari ispi r auncl .*

*AS V_1026 −But yri cicoccus uncl . AS V_118− Veil lon ell auncl .*

*AS V_721− Int esti nim onas but yri cipr oduce ns AS V_987− Er ysipel otr ich aceae uncl.*

*AS V_390− Clo str idi um Xl Va uncl . AS V_20−S ubdol igr anul um uncl .*

*AS V_981− Lachnospi ra ceaeu ncl.*

*AS V_754− Rum ino coccaceae uncl. AS V_503− Anaer ost ipes uncl .*

*AS V_261− Blau ti auncl . AS V_978− Er ysipel otr ich aceae uncl. AS V_2397 −Cl ostr idi ale suncl .*

*AS V_2138 −But yri cicoccus uncl . AS V_1936 −Cl ostr idi ale suncl . AS V_1730 −Anaer ovor ax uncl.*

*AS V_1630 −Cat abact er uncl . AS V_1430 −Rum in ococcaceae uncl.*

*AS V_1405 −Bla uti aun cl. AS V_1365 −Rum in ococcaceae uncl.*

*AS V_1264 −Bil ophi la uncl . AS V_1229 −Rum in ococcaceae uncl.*

*AS V_1216 −Anaer ot ru ncusun cl. AS V_1194 −Rum in ococcaceae uncl. AS V_1157 −Osci ll ibact er uncl .*

*AS V_1125 −Roseb uri au ncl. AS V_975− Er ysipel otr ich aceae uncl. AS V_959− Lachnospi ra ceaeu ncl.*

*AS V_927− Osci lli bact er uncl . AS V_781− Dor ea uncl.*

*AS V_780− Rum ino coccaceae uncl. AS V_744− Clo str idi um Xl Vb uncl .*

*AS V_743− Cor iob acter ia ceaeu ncl. AS V_740− Anaer ost ipes uncl .*

*AS V_695− Blau ti auncl . AS V_691− Flavon if ract or uncl . AS V_687− Flavon if ract or uncl .*

*AS V_670− Rum ino coccaceae uncl. AS V_645− Lachnospi ra ceaeu ncl. AS V_586− Clo str idi um I V lept um*

*AS V_571− Clo str idi um Xl Va uncl . AS V_569− Copr ococcus uncl .*

*AS V_566− Faecal ibact er ium un cl. AS V_565− Lachnospi ra ceaeu ncl. AS V_559− Rum ino coccus2u ncl.*

*AS V_537− Lachnospi ra ceaeu ncl. AS V_535− Blau ti auncl .*

*AS V_515− Clo str idi ales uncl . AS V_513− Lachnospi ra ceaeu ncl. AS V_493− Faecal ibact er ium un cl. AS V_491− Lachnospi ra ceaeu ncl.*

*AS V_490− Dor ea longi cat ena AS V_459− Lachnospi ra ceaeu ncl. AS V_456− Faecal ibact er ium un cl. AS V_450− Lachnospi ra ceaeu ncl.*

*AS V_447− Rum ino coccaceae uncl. AS V_443− Int esti nib acter uncl.*

*AS V_441− Clo str idi um Xl Vb uncl . AS V_421− Clo str idi um sensu st ri cto uncl .*

*AS V_418− Pseudof la vonif ra ctor un cl. AS V_407− Fusicat eni bacte r uncl. AS V_398− Rum ino coccus2u ncl.*

*AS V_381− Lachnospi ra ceaeu ncl. AS V_373− Rosebu ri aun cl. AS V_371− Blau ti auncl .*

*AS V_362− Fusicat eni bacte r uncl. AS V_361− Rosebu ri aun cl.*

*AS V_358− Rosebu ri aun cl. AS V_349− Rum ino coccaceae uncl.*

*AS V_345− Clo str idi um Xl Va uncl . AS V_325− Faecal ibact er ium pr ausn itzi i*

*AS V_318− Copr ococcus uncl . AS V_317− Dor ea uncl.*

*AS V_313− Osci lli bact er uncl . AS V_311− Faecal ibact er ium un cl. AS V_303− Blau ti auncl .*

*AS V_299− Osci lli bact er uncl .*

**top 25 %**

incl. covariates excl. covariates Proteob acteria Firmicutes Bacteroidetes Actinobacteria

Cyanobacteria.Chloroplast Ve rru comicrobia

*ASV_6−Bacteroides uniformis ASV_84−Paraprevotella uncl. ASV_90−Parabacteroides uncl. ASV_567−Subdoligranulum uncl. ASV_34−Bacteroides uncl.*

*ASV_67−Bacteroides uncl. ASV_3−Bacteroides dorei ASV_70−Parasutterella uncl. ASV_30−Bacteroides uncl. ASV_21−Bacteroides uncl. ASV_143−Alistipes uncl.*

*ASV_321−Faecalibacterium uncl.*

*ASV_1245−Ruminococcaceae uncl. ASV_57−Bacteroides uncl. ASV_5−Escherichia/Shigella uncl. ASV_551−Coprobacter uncl.*

*ASV_1844−Burkholderia uncl. ASV_385−Bacteroides uncl. ASV_174−Subdoligranulum uncl. ASV_29−Bacteroides uncl.*

*ASV_107−Bacteroides vulgatus*

*ASV_425−Faecalibacterium uncl. ASV_36−Bacteroides thetaiotaomicron ASV_10−Escherichia/ Shigella uncl.*

*ASV_890−Blautia uncl.*

*ASV_32−Dialister invisus ASV_45−Phascolarctobacterium uncl. ASV_74−Bacteroides uncl.*

*ASV_651−Coprococcus uncl.*

*ASV_1898−Oscillibacter uncl. ASV_315−Clostridium sensu stricto uncl.*

*ASV_132−Bacteroides uncl.*

*ASV_23−Parabacteroides distasonis ASV_71−Faecalibacterium uncl. ASV_47−Bacteroides f ragilis ASV_164−Parabacteroides uncl. ASV_314−Faecalibacterium uncl. ASV_153−Parasutterella uncl.*

*ASV_649−Intestinimonas uncl. ASV_610−Lachnospiraceae uncl. ASV_829−Eggerthella lenta ASV_267−Pseudoflavonifractor uncl. ASV_126−Oscillibacter uncl.*

*ASV_9−Bacteroides uncl. ASV_46−Escherichia/ Shigella coli ASV_17−Dialister invisus ASV_1035−Clostridium XlVa uncl. ASV_494−Clostridium IV uncl.*

*ASV_100−Coprobacillus uncl.*

*ASV_49−Akkermansia muciniphila ASV_81−Alistipes finegoldii ASV_8−Alistipes uncl.*

*ASV_217−Faecalibacterium uncl. ASV_152−Faecalibacterium uncl.*

*ASV_12−Parabacteroides merdae ASV_189−Alistipes massiliensis ASV_98−Anaerostipes hadrus ASV_38−Bacteroides uncl.*

*ASV_671−Clostridiales uncl. ASV_22−Bacteroides massiliensis ASV_417−Subdoligranulum uncl. ASV_576−Romboutsia uncl.*

*ASV_54−Dialister invisus ASV_62−Bacteroides thetaiotaomicron ASV_914−Clostridiales uncl.*

*ASV_2−Bacteroides uniformis ASV_104−Escherichia/Shigella uncl.*

*ASV_1476−Blautia uncl. ASV_413−Faecalibacterium uncl. ASV_136−Bacteroides uncl.*

*ASV_79−Parasutterella uncl.*

*ASV_933−Butyricicoccus uncl. ASV_142−Parasutterella uncl. ASV_1278−Faecalibacterium uncl. ASV_365−Faecalibacterium uncl. ASV_2773−Ruminococcaceae uncl. ASV_1−Bacteroides uncl.*

*ASV_667−Eggerthella uncl.*

*ASV_312−Ruminococcaceae uncl. ASV_244−Lachnospiraceae uncl.*

*AS V_298− Lachnospi ra ceaeu ncl. AS V_294− Faecal ibact er ium un cl. AS V_287− Lachnospi ra ceaeu ncl. AS V_283− Fir m icut esun cl.*

*AS V_280− Lachnospi ra ceaeu ncl. AS V_278− Faecal ibact er ium un cl. AS V_277− Lachnospi ra ceaeu ncl. AS V_273− Lachnospi ra ceaeu ncl.*

*AS V_266− Clo str idi um Xl Va uncl . AS V_264− Str ept ococcus uncl.*

*AS V_243− Bil ophi la uncl. AS V_236− Turi ciba cter u ncl.*

*AS V_235− Faecal ibact er ium un cl. AS V_231− Copr obact er uncl .*

*AS V_229− Faecal ibact er ium un cl. AS V_226− Pr oteob acter ia uncl .*

*AS V_207− Rum ino coccaceae uncl. AS V_206− Blau ti auncl .*

*AS V_194− Rom bout sia uncl .*

*AS V_184− Ent erob acter ia ceae uncl. AS V_183− Faecal ibact er ium un cl.*

*AS V_178− Haem ophi lus uncl . AS V_172− Rum ino coccus2u ncl.*

*AS V_156− Osci lli bact er uncl . AS V_138− Osci lli bact er uncl . AS V_135− Ali sti pes uncl.*

*AS V_133− Col lin sell aae rof acie ns AS V_128− Haem ophi lus uncl .*

*AS V_123− Lachnospi ra ceaeu ncl.*

*AS V_122− Rum ino coccusun cl. AS V_120− Lachnospi ra ceaeu ncl. AS V_114− Ali sti pes uncl.*

*AS V_111− Clo str idi um XV II I uncl . AS V_95−P ar abacte roi des uncl.*

*AS V_94−F aecali bact eri um uncl . AS V_80−E scher ichi a/S higel la uncl . AS V_78−R um inoco ccaceae uncl.*

*AS V_58−S utt er ell au ncl. AS V_51−C it r obacte r uncl.*

*AS V_42−E nte roba cter iace aeu ncl. AS V_19−E scher ichi a/S higel la uncl .*

*ASV_44−Bacteroides cellulosilyticus ASV_69−Subdoligranulum uncl. ASV_60−Parasutterella uncl.*

*ASV_221−Oscillibacter uncl.*

*ASV_26−Sutterella wadsworthensis ASV_382−Faecalibacterium uncl.*

*ASV_285−Blautia uncl. ASV_65−Parabacteroides distasonis*

0.0 0.1 0.2 0.3 0.4 0.5

**Taxon assoc:**

Contr/CD

Contr/UC

C CD/UC

ontr/IBD Contr/CD

Contr/UC

C CD/UC

ontr/IBD Contr/CD

Contr/UC

CD/UC

Contr/IBD

Contr/CD

Contr/UC

CD/UC

Contr/IBD

Contr/CD

Contr/UC

CD/UC

Contr/IBD

Contr/CD

Contr/UC

C CD/UC

ontr/IBD

Contr/CD

Contr/UC

C CD/UC

ontr/IBD

0.0 0.1 0.2 0.3 0.4 0.5 0.6

**Heritability estimate**

**Heritability estimate**

Control CD UC IBD

**Figure S19: (A)** Heritability estimates derived from the likelihood based method *lme4qtl* ^24^ using either only kinship information with or without additional environmental and anthropometric covariates. **(B)** The upper 25% percentile of taxa are highlighted (based on *h^2^* estimate including environmental covariate). Additional information like differential abundance in IBD accross cohorts (Table S25).

**Figure S20:** Process of study participant enrollment. Abbreviations: IBD, inflammatory bowel disease; IC, informed consent.

## Assessment phase

1040 IBD patients

| 1246 healthy family members | |
| --- | --- |
|  | *2 years* |

Baseline

122 IBD patients

42 IBD patients

Follow-up 1

| 55 healthy family members | |
| --- | --- |
|  | *2 years* |

Follow-up 2

| 181 healthy family members | |
| --- | --- |
|  | *2 years* |

Follow-up 3

325 healthy family members

299 IBD patients

**Figure S21:** Current number of study participants (IBD patients and healthy family members) by assessment phase in the prospective Kiel IBD Family Cohort. Abbreviations: IBD, inflammatory bowel disease.

*3 reminders*

Immediate dispatch to the newly diagnosed IBD patient:

- biomaterial collection tubes
- participant questionnaires
- physician questionnaires

Report/notice of a new IBD-onset case

*no*

Return of study material

*incomplete*

Reminder for study material

Wait for follow-up

**Figure S22:** Procedure following the report of a new IBD onset case during study follow-up.

37

## Supplemental tables:

**Table S1:** Overview of items included in the different questionnaires of the Kiel IBD Family Cohort BL=baseline assessment; F1=since first follow-up assessment; F2=since second follow-up assessment.

Abbreviations: CDAI, Crohn’s Disease Activity Index; FSS, Fatigue Severity Scale; HBI, Harvey-Bradshaw-Index; IBD, inflammatory bowel disease.)

**Table S2:** Overview of biomaterial sample collection, processing, and storage time between processing and storage.

**Table S3:** Distribution of IBD types within the IBD patients (n=1321) of the Kiel IBD Family Cohort. Abbreviations: CD, Crohn’s disease; IBD, inflammatory bowel disease; UC, Ulcerative colitis; uIBD, unclassified inflammatory bowel disease.

**Table S4:** Baseline characteristics of unaffected (healthy) relatives of IBD patients (n=1072) in the Kiel IBD Family Cohort, stratified by age group. Values are median (IQR) or absolute and relative frequencies. Abbreviations: IBD, inflammatory bowel disease.

**Table S5:** Analysis of major physiological characteristics across time points using linear models. Models are optimized by variable selection minimizing AIC (BL, F1, F2).

**Table S6:** Linear model analyses of clinical inflammation markers with respect to *LDpred2* Polygenic Risk Scores (CD, UC and general IBD), IBD pathology, and other covariates. Analyses were performed on baseline samples (BL) ^7^.

**Table S7:** Correlation of alpha diversity with the MD-index across baseline ^11^, follow-up 1, and follow-up 2 using either a linear or polynomial (quadratic) fit, as based on minimal AIC. Models were either adjusted for covariates or tested without considering covariates.

**Table S8:** Analyses of the relationship between alpha diversity measures (Chao1 species richness, Shannon Diversity (effective number)) and clinical markers of inflammation (ASCA IgA/IgG, GP2 IgA/IgG, calprotectin, Bristol stool score, Hb, CRP, IBD severity (clinician)). Analyses were performed on the residuals of the respective markers, after fitting a linear model including age, BMI, and sex.

**Table S9:** Differential abundance analyses of baseline samples (BL) at the ASV level (RPD16 based classification included) via negative binomial models (*DESeq2*, Wald test) [9], between control individuals, CD and UC patients (excluding uIBD), and between control individuals and IBD patients (CD, UC, uIBD) ^8^. Models were adjusted by age, BMI and sex as covariates. All P values were adjusted via FDR. Additional support from external independent cohorts is included based on DA following the same methodology (Malta treatment naive, Malta remission, Sweden-SIC).

**Table S10:** Differential abundance analyses of samples from the first follow-up (F1) at the ASV level (RPD16 based classification included) via negative binomial models (*DESeq2*, Wald test) ^8^, between control individuals, CD and UC patients (excluding uIBD), and between control individuals and IBD patients (CD, UC, uIBD). Models were adjusted by age, BMI and sex as covariates. All P values were adjusted via FDR. Additional support from external independent cohorts is included based on DA following the same methodology (Malta treatment naive, Malta remission, Sweden-SIC).

**Table S11:** Differential abundance analyses of samples from the second follow-up (F2) at the ASV level (RPD16 based classification included) via negative binomial models (*DESeq2*, Wald test) ^8^, between control individuals, CD and UC patients (excluding uIBD), and between control individuals and IBD patients (CD, UC, uIBD). Models were adjusted by age, BMI and sex as covariates. All P values were adjusted via FDR. Additional support from external

independent cohorts is included based on DA following the same methodology (Malta treatment naive, Malta remission, Sweden-SIC).

**Table S12:** Differential abundance analyses of combined samples (BL, F1, F2) at the ASV level (RPD16 based classification included) via negative binomial models (*DESeq2*, Wald test) ^8^, between control individuals, CD and UC patients (excluding uIBD), and between control individuals and IBD patients (CD, UC, uIBD). Models were adjusted by age, BMI, sex, and time point as covariates. All P values were adjusted via FDR. Additional support from external independent cohorts is included based on DA following the same methodology (Malta treatment naive, Malta remission, Sweden-SIC).

**Table S13:** Partial correlation of CLR transformed taxon abundances^25^ with physiological/clinical measures and *LDpred2* derived polygenic risk scores (PRS) ^7^ for CD, UC, and IBD via *ppcor* ^9^. P-values were derived from combining the *P* values of Spearman-, Kendall-, and Pearson correlations via Brown’s method and corrected via FDR ^10^. Correlations were adjusted for age, gender and BMI. The table includes additional information of overlapping and significant differential abundance patterns in the KINDRED cohort, Maltese-, and Swedish SIC cohort.

**Table S14:** Linear model analyses of alpha diversity (Chao1 species richness, Shannon H (number equivalent)) and MD-index in relation to IBD condition, including differences in average diversity changes as well as disease specific correlations between alpha diversity and MD-index ^11^.

**Table S15:** Betadiversity analyses of Bray-Curtis dissimilarity based on ASV abundances. Analyses were done using PERMANOVA ^26^ either with or without conditioning for potential covariates (age, BMI, sex). Analyses were performed globally, pairwise, as well dysbiosis scores in the different sampling time points (Baseline, Follow-up 1, Follow-up 2, excluding

uIBD). Pairwise comparisons were corrected for multiple testing via FDR.

**Table S16:** Betadiversity analyses of Bray-Curtis dissimilarity based on ASVs focusing on physiological/clinical- and anthropometric measures at different sampling time points (Baseline, Follow-up 1, Follow-up 2).

**Table S17:** Community variability analysis of Bray-Curtis dissimilarity via PERMANOVA, as based on ASV abundances focusing on IBD pathologies across sampling time points (Baseline, Follow-up 1, Follow-up 2) ^14^.

**Table S18:** Pairwise PERMANOVA analyses of ASV based Bray-Curtis dissimilarities of combined KINDRED, Maltese, and Swedish cohorts, based on the main pathologies.

**Table S19:** Linear model analyses of dysbiosis scores with respect to IBD pathology, and other covariates (excluding uIBD). Analyses were performed on the residuals of the respective markers, after fitting a linear model including age, BMI, and sex and then selected by minimizing AIC.

**Table S20:** Betadiversity analysis of Bray-Curtis dissimilarity based on ASV abundances focusing on pathologies and morbidities reported by the subjects across sampling time points (Baseline, Follow-up 1, Follow-up 2).Naive and conditioned PERMANOVA results, corrected for age, sex, BMI and IBD.

**Table S21:** Betadiversity analyses of Bray-Curtis dissimilarity based on ASV abundances focusing on medical/pharmaceutical treatments reported by the subjects across sampling time points (Baseline, Follow-up 1, Follow-up 2). Naive and conditioned PERMANOVA results, corrected for age, sex, BMI and IBD.

**Table S22:** Betadiversity analyses of Bray-Curtis dissimilarity based on ASV abundances focusing on normalized nutrient intake derived from 2 week food frequency questionnaires, across sampling time points (Baseline, Follow-up 1, Follow-up 2). Naive and PERMANOVA results, corrected for age, sex, BMI.

**Table S23:** Network importance/centrality measures derived at the different time points (BL, F1, F2) of the KINDRED cohort, as well as subsets by helath condition (CD, UC, Controls) within each time point. Results of differential abundance associations, as well as potential role in disease onset or remission, for each significant node/taxon are included. Centralities range from the number of connections (degree), the position on the shortest paths within the network (betweeness, ^16^, generalized importance (PageRank index ^17^ and the average neighborhood degree of any given vertex (k-nearest neighbor degree ^18^. Significance of centralities is derived from Z-test against a collection of randomized network centralities (FDR corrected). Taxa which are significantly more central than expected by chance, and consistently over-abundant in healthy individuals are highlighted in lightblue, taxa consistently over-abundant in IBD cases (CD/UC) are highlighted in orange.

**Table S24:** Taxon heritability as derived from linear mixed models including kinship matrices. Heritability (*h^2^*) was estimated with and without environmental variables via *lme4qtl*

^24^. The table includes differential abundance patterns in KINDRED and external cohorts (incl. their summary). Values indicating a better fit solely by pedigree information (yellow) or better fit when covariates (sex, age, BMI, IBD pathology) are included in addition to pedigree information.

## Supplemental references:

1. EuroQol Group. EuroQol--a new facility for the measurement of health-related quality of life. Health Policy 1990; 16:199–208.
2. Ludwig K, Graf von der Schulenburg J-M, Greiner W. German Value Set for the EQ- 5D-5L. Pharmacoeconomics 2018; 36:663–74.
3. Nöthlings U, Hoffmann K, Bergmann MM, Boeing H. Fitting portion sizes in a self- administered food frequency questionnaire. J Nutr 2007; 137:2781–6.
4. Haftenberger M, Schuit AJ, Tormo MJ, Boeing H, Wareham N, Bueno-de-Mesquita HB, Kumle M, Hjartåker A, Chirlaque MD, Ardanaz E, et al. Physical activity of subjects

aged 50-64 years involved in the European Prospective Investigation into Cancer and Nutrition (EPIC). Public Health Nutr 2002; 5:1163–76.

1. Wilkinson MD, Dumontier M, Aalbersberg IJJ, Appleton G, Axton M, Baak A, Blomberg N, Boiten J-W, da Silva Santos LB, Bourne PE, et al. The FAIR Guiding Principles for scientific data management and stewardship. Sci Data 2016; 3:160018.
2. Mirzayi C, Renson A, Furlanello C, Sansone SA, Zohra F, Elsafoury S, Geistlinger L, Kasselman LJ, Eckenrode K, van de Wijgert J, et al. Reporting guidelines for human microbiome research: the STORMS checklist. Nature Medicine 2021; 27:1885–92.
3. Privé F, Arbel J, Vilhjálmsson BJ. LDpred2: better, faster, stronger. Bioinformatics 2021; 36:5424–31.
4. Love MI, Huber W, Anders S. Moderated estimation of fold change and dispersion for RNA-seq data with DESeq2. Genome Biology 2014; 15:550.
5. Kim S. ppcor: An R Package for a Fast Calculation to Semi-partial Correlation Coefficients. Communications for Statistical Applications and Methods 2015; 22:665– 74.
6. Brown MB. 400: A Method for Combining Non-Independent, One-Sided Tests of Significance. Biometrics 1975; 31:987–92.
7. Gevers D, Kugathasan S, Denson LA, Vázquez-Baeza Y, Van Treuren W, Ren B, Schwager E, Knights D, Song SJ, Yassour M, et al. The treatment-naive microbiome in new-onset Crohn’s disease. Cell Host and Microbe 2014; 15:382–92.
8. Chao A. Estimating the Population Size for Capture-Recapture Data with Unequal Catchability. Biometrics 1987; 43:783.
9. Jost L. Entropy and diversity. Oikos 2006; 113:363–75.
10. Anderson MJ. Distance-based tests for homogeneity of multivariate dispersions. Biometrics 2006; 62:245–53.
11. Ding T, Schloss PD. Dynamics and associations of microbial community types across the human body. Nature 2014; 509:357–60.
12. Freeman LC. Centrality in social networks conceptual clarification. Social Networks 1978; 1:215–39.
13. Page L, Brin S. The anatomy of a large-scale hypertextual Web search engine. Computer Networks 1998; 30:107–17.
14. Barrat A, Barthélemy M, Pastor-Satorras R, Vespignani A. The architecture of complex weighted networks. Proceedings of the National Academy of Sciences of the United States of America 2004; 101:3747–52.
15. Yaveroğlu ÖN, Malod-Dognin N, Davis D, Levnajic Z, Janjic V, Karapandza R, Stojmirovic A, Pržulj N. Revealing the Hidden Language of Complex Networks. Sci Rep 2014; 4:4547.
16. Newman MEJ. Assortative Mixing in Networks. Physical Review Letters 2002; 89:208701.
17. West DB. Introduction to Graph Theory. Prentice Hall; 2001.
18. Wasserman S, Faust K. Social Network Analysis: Methods and Applications. Cambridge University Press; 1994.
19. Jun W, Barahona M, Yue-Jin T, Hong-Zhong D. Natural Connectivity of Complex Networks. Chinese Phys Lett 2010; 27:078902.
20. Ziyatdinov A, Vázquez-Santiago M, Brunel H, Martinez-Perez A, Aschard H, Soria JM. lme4qtl: linear mixed models with flexible covariance structure for genetic studies of related individuals. BMC Bioinformatics 2018; 19:1–5.
21. van den Boogaart KG, Tolosana-Delgado R, Bren M. compositions: Compositional Data Analysis [Internet]. 2023; Available from:

https://CRAN.R-project.org/package=compositions <http://www.stat.boogaart.de/compositions/>

1. Anderson MJ. A new method for non-parametric multivariate analysis of variance. Austral Ecology 2001; 26:32–46.
2. EuroQol Group. EuroQol--a new facility for the measurement of health-related quality of life. Health Policy 1990; 16:199–208.
3. Ludwig K, Graf von der Schulenburg J-M, Greiner W. German Value Set for the EQ- 5D-5L. Pharmacoeconomics 2018; 36:663–74.
4. Nöthlings U, Hoffmann K, Bergmann MM, Boeing H. Fitting portion sizes in a self- administered food frequency questionnaire. J Nutr 2007; 137:2781–6.
5. Haftenberger M, Schuit AJ, Tormo MJ, Boeing H, Wareham N, Bueno-de-Mesquita HB, Kumle M, Hjartåker A, Chirlaque MD, Ardanaz E, et al. Physical activity of subjects aged 50-64 years involved in the European Prospective Investigation into Cancer and Nutrition (EPIC). Public Health Nutr 2002; 5:1163–76.
6. Wilkinson MD, Dumontier M, Aalbersberg IJJ, Appleton G, Axton M, Baak A, Blomberg N, Boiten J-W, da Silva Santos LB, Bourne PE, et al. The FAIR Guiding Principles for scientific data management and stewardship. Sci Data 2016; 3:160018.
7. Mirzayi C, Renson A, Furlanello C, Sansone SA, Zohra F, Elsafoury S, Geistlinger L, Kasselman LJ, Eckenrode K, van de Wijgert J, et al. Reporting guidelines for human microbiome research: the STORMS checklist. Nature Medicine 2021; 27:1885–92.
8. Privé F, Arbel J, Vilhjálmsson BJ. LDpred2: better, faster, stronger. Bioinformatics 2021; 36:5424–31.
9. Love MI, Huber W, Anders S. Moderated estimation of fold change and dispersion for RNA-seq data with DESeq2. Genome Biology 2014; 15:550.
10. Kim S. ppcor: An R Package for a Fast Calculation to Semi-partial Correlation Coefficients. Communications for Statistical Applications and Methods 2015; 22:665– 74.
11. Brown MB. 400: A Method for Combining Non-Independent, One-Sided Tests of Significance. Biometrics 1975; 31:987–92.
12. Gevers D, Kugathasan S, Denson LA, Vázquez-Baeza Y, Van Treuren W, Ren B, Schwager E, Knights D, Song SJ, Yassour M, et al. The treatment-naive microbiome in new-onset Crohn’s disease. Cell Host and Microbe 2014; 15:382–92.
13. Chao A. Estimating the Population Size for Capture-Recapture Data with Unequal Catchability. Biometrics 1987; 43:783.
14. Jost L. Entropy and diversity. Oikos 2006; 113:363–75.
15. Anderson MJ. Distance-based tests for homogeneity of multivariate dispersions. Biometrics 2006; 62:245–53.
16. Ding T, Schloss PD. Dynamics and associations of microbial community types across the human body. Nature 2014; 509:357–60.
17. Freeman LC. Centrality in social networks conceptual clarification. Social Networks 1978; 1:215–39.
18. Page L, Brin S. The anatomy of a large-scale hypertextual Web search engine. Computer Networks 1998; 30:107–17.
19. Barrat A, Barthélemy M, Pastor-Satorras R, Vespignani A. The architecture of complex weighted networks. Proceedings of the National Academy of Sciences of the United States of America 2004; 101:3747–52.
20. Yaveroğlu ÖN, Malod-Dognin N, Davis D, Levnajic Z, Janjic V, Karapandza R, Stojmirovic A, Pržulj N. Revealing the Hidden Language of Complex Networks. Sci Rep 2014; 4:4547.
21. Newman MEJ. Assortative Mixing in Networks. Physical Review Letters 2002; 89:208701.
22. West DB. Introduction to Graph Theory. Prentice Hall; 2001.
23. Wasserman S, Faust K. Social Network Analysis: Methods and Applications. Cambridge University Press; 1994.
24. Jun W, Barahona M, Yue-Jin T, Hong-Zhong D. Natural Connectivity of Complex Networks. Chinese Phys Lett 2010; 27:078902.
25. Ziyatdinov A, Vázquez-Santiago M, Brunel H, Martinez-Perez A, Aschard H, Soria JM. lme4qtl: linear mixed models with flexible covariance structure for genetic studies of related individuals. BMC Bioinformatics 2018; 19:1–5.
26. van den Boogaart KG, Tolosana-Delgado R, Bren M. compositions: Compositional Data Analysis [Internet]. 2023; Available from:

https://CRAN.R-project.org/package=compositions <http://www.stat.boogaart.de/compositions/>

1. Anderson MJ. A new method for non-parametric multivariate analysis of variance. Austral Ecology 2001; 26:32–46.
